# Three-dimensional structure of entire hydrated murine hearts at histological resolution

**DOI:** 10.1038/s41598-024-83853-y

**Published:** 2025-01-22

**Authors:** Jasper Frohn, Frederik Böddeker, Marius Reichardt, Hendrik Bruns, Titus Czajka, Amara Khan, Ludovic Broche, Michael Krisch, Alberto Bravin, Frauke Alves, Jana Zschüntzsch, Tim Salditt

**Affiliations:** 1 Institute for X-ray Physics, Georg-August University Göttingen, Friedrich-Hund-Platz 1, 37077 Göttingen, Germany; 2 Georg-August University Göttingen, University Medical Center Göttingen, 37075 Göttingen, Germany; 3 European Synchrotron Radiation Facility, Grenoble, France; 4 Multiscale Bioimaging: From Molecular Machines to Networks of Excitable Cells (MBExC), Grenoble, France

**Keywords:** Virtual 3d histology, Cardiomyocyte network, X-ray phase-contrast, X-ray tomography, Organ imaging, Cardiology, Imaging techniques

## Abstract

Imaging the entire cardiomyocyte network in entire small animal hearts at single cell resolution is a formidable challenge. Optical microscopy provides sufficient contrast and resolution in 2d, however fails to deliver non-destructive 3d reconstructions with isotropic resolution. It requires several invasive preparation steps, which introduce structural artefacts, namely dehydration, physical slicing and staining, or for the case of light sheet microscopy also clearing of the tissue. Our goal is to provide 3d reconstructions of the cardiomyocyte network in entire hydrated murine hearts, and to develop a methodology for quantitative analysis of heart pathologies based on X-ray phase contrast computed tomography (XPCT). We have used XPCT at two beamlines of the extremely brilliant source (EBS) at the European Synchrotron Radiation Facility (ESRF) to scan wild-type murine hearts at high resolution, as well as a series of murine hearts of different pathological models, at reduced resolution and higher throughput. All hearts were obtained from the small animal facility of the university medical center in Göttingen. The hearts were fixed in formalin, stored and measured non-destructively in phosphate buffer solution. The high resolution dataset allows to discern individual cardiomyocytes in the tissue. All datasets have been analyzed using semi-automated image segmentation of the ventricles, rotation into a common coordinate system, classification into different anatomical compartments, and finally the structure tensor approach. A 3d streamline representation of the cardiomyocyte orientation vector field is provided. The different cardiovascular disease models are analysed based on metrics derived from the 3d structure tensor. An entire hydrated murine heart has been covered at an isotropic voxel size of 1.6$\upmu$m (distributed over several volumes). A binned and fused dataset of this heart is available at 3.2$\upmu$m, and has been analyzed by the structure tensor approach to yield the ventricular cardiomyocyte network or mesh, i.e. the aggregation of the cardiomyocyte chains in particular in the ventricular wall. Semi-automatic determination of structural metrics is already achieved and the corresponding tools and resulting data are made publically available. XPCT using extremely brilliant undulator radiation is close to achieve single cell reconstruction in an entire small animal organ.

## Introduction

Cardiac imaging of small animal models is indispensable for cardiovascular research. While magnetic resonance imaging (MRI) and ultrasound are used to image heart contraction dynamically, they cannot resolve the cytoarchitecture down to the level of a single cardiomyocyte^1–5^. Contrarily, with modern micro-computed tomography (CT), entire small animal hearts can now be scanned non-destructively after post mortem organ removal and fixation. With enhanced contrast for unstained cardiac tissue and voxel sizes in the range of a few micrometers, using synchrotron radiation (SR)^6–13^, as well as laboratory X-rays^14^, it has now become possible to characterize the three-dimensional (3d) structure, linking anatomical and histological scales. This meets a need in cardiovascular research, since the invasive nature of sectioning largely impedes a detailed 3d reconstruction of the muscle cell and myofibril arrangement in the heart^15,16^. While there is consensus in considering the heart as a complex 3d mesh of aggregated cardiomyocytes with a supporting fibrous matrix^17,18^, different models of cardiac architecture are still under debate^18,19^. More generally, imaging the intricate multi-scale structure of the heart remains a formidable challenge^20^: from the molecular scales of myosin motors and the actomyosin assembly, to the formation of sarcomeric units and myofibrils in cardiomyocytes, to the arrangement of myocyte chains embedded in connective tissue, and then the aggregation of cardiomyocyte chains finally forming the complex cardiac mesh up to the entire organ^17,21^. While conventional CT is based on absorption and shows little contrast for heart tissue without heavy metal staining^22–24^, X-ray phase contrast computed tomography (XPCT) is sensitive to small electron density variations within unstained tissue, since electron density $\rho _e$ is proportional to the real-valued decrement $\delta$ of the X-ray refractive index $n= 1- \delta + i\beta$. Depending on tissue composition and wavelength $\lambda$, $\delta$ is several orders of magnitude larger than the imaginary part $\beta$ which accounts for absorption. Phase-contrast X-ray imaging can hence visualize even small features down to cellular and sub-cellular scale for heart tissue biopsies^25^, showing myofibris and as well as the network and degree of myocyte alignment, orientation and ordering. In contrast to X-ray diffraction, by which molecular structural parameters of acto-myosin complexes can be assessed^26,27^, XPCT is a full field technique compatible with 3d imaging of the whole heart, without physical sectioning. However, preservation of structure and suitable embedding media are a major concern. Formalin fixed paraffin embedded (FFPE) heart tissue offers strong contrast even for laboratory XPCT^14^but at the expense of pronounced tissue fissures. Ethanol embedding offers higher quality and preservation, but requires improved illumination and beam coherence, and suffers from dehydration artefacts such as shrinkage^7,12^. Heart imaging in solution, notably in phosphate saline buffer (PBS) is preferable, but is significantly more challenging for XPCT in terms of resolution, contrast and overall image quality^28^.

**Fig. 1:**
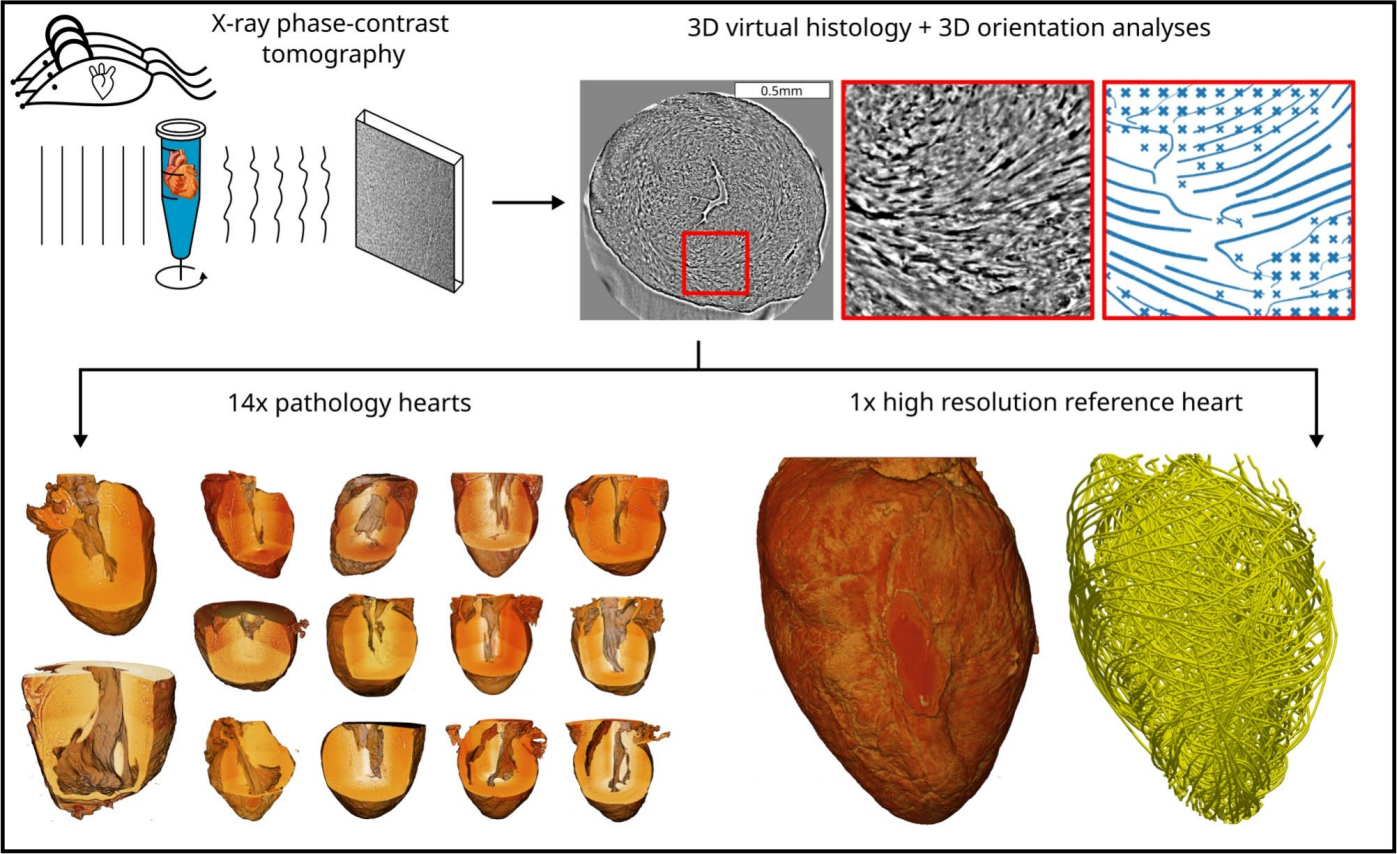
Illustration of the XPCT experiment and analysis. Entire murine hearts fixed in formalin and stored in phosphate buffer solution are measured by propagation-based X-ray phase-contrast tomography. The image quality in terms of contrast and resolution is sufficient to track single cardiomyocytes and the 3d orientation vector field of cardiomyocyte chains within the entire heart. A 3d orientation analysis is performed on 14 hearts of different pathological models. A control murine heart is measured at a higher resolution.

In this work, we present XPCT using 4th generation synchrotron radiation at beamline ID19/ESRF-EBS to reconstruct an entire murine heart in PBS at a pixel size of 3.2$\upmu$m (2x 1.6$\upmu$m binned) and high image quality, allowing to reconstruct the orientation vector field of myocyte aggregates with minimal artefacts, see the overview in Fig 1. Apart from the goal to create a reference dataset for a hydrated murine heart, the second motivation for the present study is to explore to which extent models for different cardiovascular diseases including in particular hypertrophy and myocardial infarction can be investigated and analysed semi-automatically, based on metrics derived from the structure tensor. In order to preserve the anatomical context, the 3d histological metrics are evaluated based on the location of the tissue within the organ, using a segment model proposed by the American heart association^29^. To this end, we have devised an automated segmentation and masking process for each heart. A suitable coordinate system based on second moments is then chosen automatically. Thus, we can meet the challenge that each heart has a somewhat different position in the measurement container.

Earlier work by Planinc et al. has already shown that XPCT can be used advantageously to study pathological cytoarchitecture and anatomy in small animal disease models^28^. Here we extend the 3d morphological analysis of pathological conditions to three hypertrophy models, including (1) a permanent constriction placed around the transverse aorta (TAC), (2) an arteriovenous fistula (AVF) resulting in an aortocaval shunt, and (3) pressure overload and recovery heart model based on TAC and subsequent debanding of the aorta. Further, we include (4) a myocardial infarction (MI) model, and (5) a Duchenne muscular dystrophy (DMD) model. Rather than aiming at a preclinical study with statistically sufficient number of cases *N* for each condition, we here content ourselves with a total of $N_{tot}=14$ hearts, exploring the possible range of effects and variations, and the suitabiliy of different morphometric parameters, linking the anatomical and histological level. In particular, we aim at a further specification of the conditions which automated imaging and analysis workflows have to meet. Scans for the pathological heart models and controls are carried out at the medical beamline ID17/ESRF.

The manuscript is organized as follows: After this introduction, the Methods & Materials section presents sample preparation and disease models, the 3d imaging, and the analysis workflows. The method section then begins with the presentation of the physiological reference dataset obtained at beamline ID19, before the series of 14 hearts scanned at ID17 is presented. The manuscript closes with the discussion and conclusion section. Further details on phase retrieval, segmentation, coordinate systems and stitching of reconstruction volumes are given in the appendix. All datasets and tools are made publicly available for further use.

## Methods

### XPCT and 3d virtual histology

**Fig. 2:**
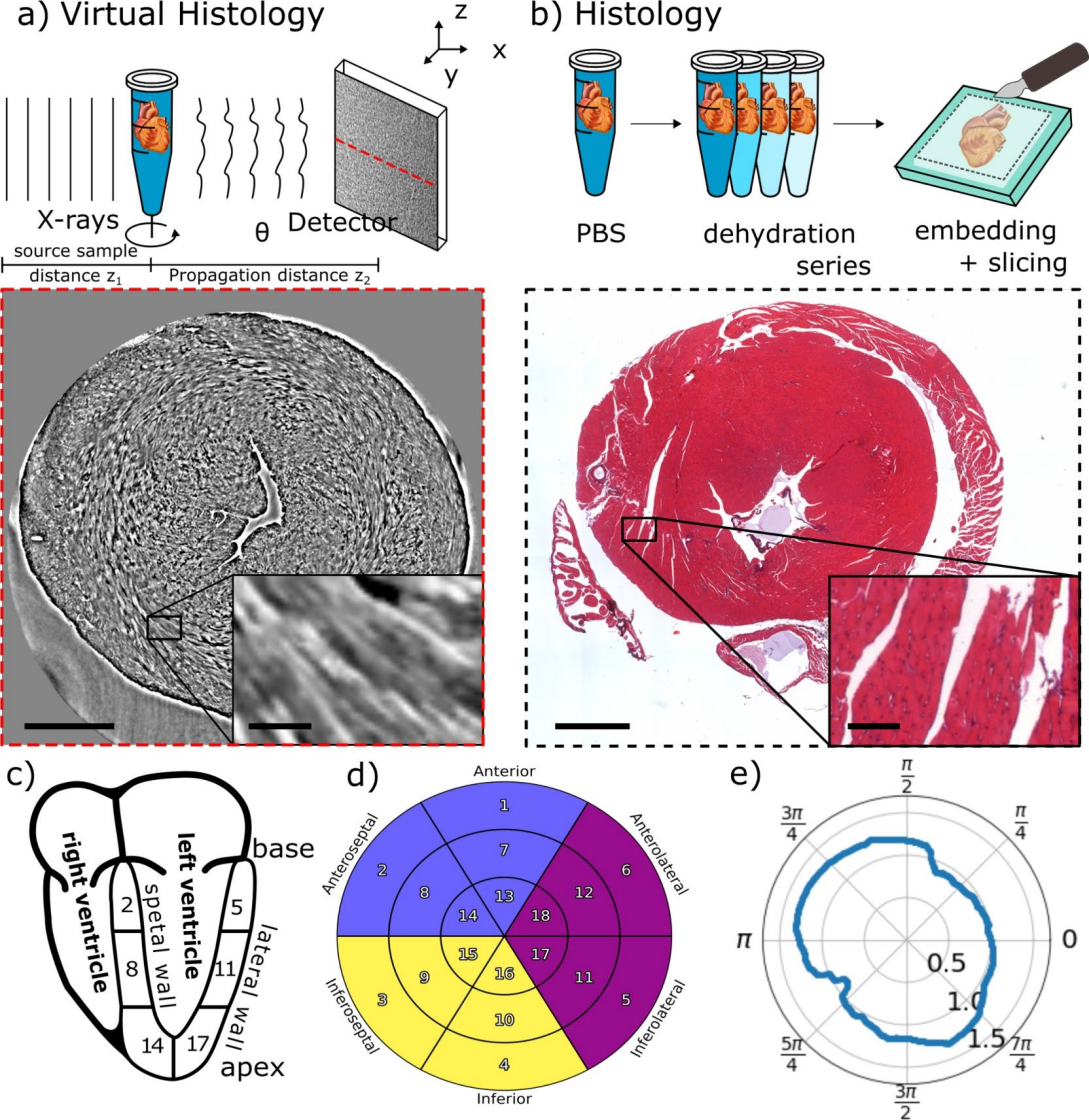
Comparison of the sample preparation and image quality of virtual 3d histology and conventional histology, based on *H* &*E* stained sections. (**a**) Virtual histology: The murine heart is placed in an Eppendorf tube filled with phosphate buffered saline (PBS). The Eppendorf tube is mounted on a rotational stage to acquire the X-ray phase-contrast projections in a tomographic scan. After the tomographic reconstruction of the 3d volume, slices of arbitrary orientation within the volume can be inspected virtually. Note the intact tissue structure of the murine heart in the virtual slice shown. (**b**) Conventional *H* &*E* histology: The mouse heart in PBS is first dehydrated and then embedded in a paraffin block. Slices of the paraffin block are physically sectioned from the top. The tissue slice is stained e.g. with H&E and investigated under an optical microscope. An exemplary slice of murine heart tissue shows a ruptured tissue structure e.g. highlighted in the inset. Scale bars: 1 mm, insets 100$\upmu$m. (**c**) A heart cross-section along the long axis is shown with the numbered segments, the ventricles and the heart walls. (**d**) 18 segment bullseye plot, representing different numbered segments of the heart, as proposed by the American Heart Association (AHA)^29^. The segments names refer to the position of the segment relative to the centre of the left ventricle and the position between the base and the apex. (**e**) The heart wall thickness *d* as a radial plot, shown for a traverse plane. The origin is at the centre of the left ventricle. The displayed data is from the ID19 acquisition (reference heart).

 X-ray phase-contrast computed tomography (XPCT) can connect the micro-anatomical scale with the histological scale, i.e. the scale at which cells, and sub-cellular features are resolved. XPCT is non-destructive, compatible with hydrated organs, and enables 3d virtual histology with isotropic resolution. This includes in particular the possibility to virtually slice the reconstructed 3d sample volume in any desired plane, and to implement metrics which operate in 3d. The experimental setup is sketched in Fig. 2(a). The unstained murine heart is stored in an Eppendorf tube (1.5mL) filled with PBS. The tube is carefully degassed in a vacuum chamber to reduce the creation of air bubbles during illumination with X-ray. Partially coherent X-rays are generated by a wiggler (ID17) or undulator (ID19) and propagate through the object. The phase-contrast images were recorded on an Optique peter imaging system with sCMOS PCO edge 5.5 (ID17) and a FRELON camera (ID19). The phase shifts imposed by the tissue are reconstructed from the measured intensity images (phase retrieval). From the phase-contrast images corresponding to a full series of projection angles $\theta$, a 3d reconstruction is obtained by computed tomography. The orientation of the exemplary virtual slice of the reconstructed volume is indicated by the red line on the detector in Fig 2(a). The shown virtual slice corresponds to a cross section through a murine control heart with intact tissue structure. The image contrast represents the electron density differences within the tissue. The left ventricle is visible in the center of the slice as well as the muscle fibers, as highlighted in the inset. In comparison, conventional histology requires additional preparation steps, as shown in Fig. 2(b). First, the chemically fixated sample needs to be dehydrated e.g. by an ascending ethanol series. Afterwards, the sample is embedded in paraffin. In order to obtain an microscopic image of the sample, it is physically sliced into thin sections followed by a staining step to enhance image contrast e.g. by an *H* &*E* staining. By way of example, a microscopic image of a histological section of a murine heart is shown, exhibiting high image contrast and single cell resolution. However, artefacts resulting from the additional sample preparation steps occur, e.g. shrinking of the tissue or tissue ruptures of the muscle fibre occur, also illustrated in the inset.

### Sample preparation and pathological models

Five different pathological models of heart diseases are investigated in this study, as summarized in Tab.1. The pathologies were either genetic or induced in eight-week-old C57BI/6 mice, at the laboratory small animal facility of the Göttingen university medical center. Hypertrophy 1: A pressure overload heart model was created in the left ventricle (LV) by permanent constriction placed around the transverse aorta (TAC), limiting left ventricular (LV) outflow. TAC can produce profound adverse cardiac remodelling such as hypertrophy and fibrosis, as well as systolic and diastolic cardiac dysfunction and long-term progression to heart failure^30^and enlarged ventricular mass. The mice were euthanized 10 weeks post the TAC surgery and whole hearts were excised. Sham animals underwent the same procedure except the banding of the aorta. Hypertrophy 2: A volume overload heart model was created in the left ventricular (LV) by an arteriovenous fistula (AVF) between the infrarenal descending aorta and the inferior vena cava producing an aortocaval shunt. Aortocaval shunt is characterized by left and right ventricular hypertrophy and cardiac dilatation, as well as myocardial remodeling. Shunt mice were also sacrificed 10 weeks post surgery, and hearts were explanted. Sham animals underwent the same procedure except for the puncture of the vessels. Hypertrophy 3: A pressure overload and recovery heart model was created by a applying a TAC for 4 weeks followed by the debanding of the aorta. The mice were euthanized 2 weeks post the debanding surgery and whole hearts were excised. Sham animals underwent the same procedure except the banding of the aorta. Myocardial infarction: A myocardial infarction (MI) model was created by a permanent ligation of the left anterior descending coronary artery (LAD). Adult myocardial tissue lacks regenerative capacity, representing a permanent injury, leading to replacement fibrosis and a permanent remodeling of the heart, such as a thinning of the infarcted area in the LV wall. The mice were euthanized 10 weeks post the MI surgery and whole hearts were excised. Sham animals underwent same procedure except the ligation of LAD. Duchenne muscular dystrophy (DMD): As a classical biochemical and genetic mouse model of DMD, the X chromosome-linked muscular dystrophy (mdx) mouse was used^31,32^. The mdx mutation occurred spontaneously due to a premature stop codon resulting in a termination in exon 23 of the dystrophin gene^33^. The mdx mice (C57BL/ 10ScSn mdx) used for breeding were kindly provided by Ralf Herrmann (University of Essen, Germany). Male mice were heterozygous and female mice homozygous for the mdx gene. The mdx mice have except of some revertant fibers a lack of the dystrophin protein, which results in a disrupted dystrophin-glycoprotein-complex. Subsequently, a sarcolemmal instability, an increased vulnerability to mechanical stress, changes in calcium homeostasis, and the replacement of muscle tissue by adipose and connective tissue can be detected^34,35^and are responsible for the skeletal muscle and cardiopulmonal phenotype^36–38^. All heart samples were chemically fixed (4% PFA) and stored in PBS after excision. An overview of all samples is given in Tab.1.

**Table 1:** Sample number, name and pathology of the hearts. The healthy heart denoted as Control_ID19 was scanned at ID19; all other hearts were scanned at ID17.

Sample number	Heart name	Pathology
S7236	Control_ID19	Healthy
S1833	DMD1	Dystrophy
S6571	TAC1	Hypertrophy I
S7073	Control1	Healthy
S7242	MI1	Myocardial infarction
S7243	MI2	Myocardial infarction
S7246	Control2	Healthy
S7249	TAC2	Hypertrophy I
S7250	TAC3	Hypertrophy I
S7252	TAC4	Hypertrophy I
S7254	de-TAC1	Hypertrophy III
S7255	de-TAC2	Hypertrophy III
S7260	Shunt1	Hypertrophy II
S7261	Shunt2	Hypertrophy II
S7262	Shunt3	Hypertrophy II

### Experimental setup and imaging parameters

XPCT experiments were performed at two synchrotron micro-tomography instruments. A healthy murine heart was scanned at the the ID19 undulator beamline^39^, in two configurations (zoom levels), denoted as high and low resolution configuration. The gap of the u17-6c undulator was set to 28 mm to generate X-rays of 19keV energy. An X-rays microscope system (optique peter) was used with a 10$\upmu$m thick GGG scintillator and a pco.edge 5.5 camera. The camera was coupled with a 4x and 10x magnifying objective, resulting in a pixel size of 1.608$\upmu$m, and 0.649$\upmu$m, for the low and high resolution configurations, respectively. The propagation distance between object and detector was $z_{12}=\,\textrm{mm}$ and $z_{12}={170}\,\textrm{mm}$, the low and high resolution configurations, respectively. 1500 projection were acquired over angular range of 180$^{\circ }$. In the low resolution configuration, 5 tomograms were acquired per height level for 4 neighboring height levels resulting in 20 tomograms in total for the entire object. After reconstruction of the individual volumes and subsequent stitching, artefacts of stitching were removed by highpass filtering with an 8 pixel Gaussian filter, to minimize the remaining low spatial frequency ring artefacts of the different tomograms. All imaging parameters are tabulated in the appendix Tab.2. The ID19 scans do not show any shrinking and rupture artifacts, nor any bubbles which are often encountered in synchrotron micro-tomography experiments. The resulting dataset can serve as a 3d atlas of murine heart, in particular in view of the superior image quality. The entire series of murine hearts with the pathological models was scanned at the ID17 wiggler beamline^40^, using a single configuration, tailored to higher throughput of samples. The murine hearts were stored in buffer solution (1.5 ml Eppendorf tubes), degased and shipped to ESRF, where they were scaned in in hydrated conditions. The peak photon energy of the wiggler was set to 50 keV (wiggler gap 94 mm). The spectrum of the x-ray energy distribution was further adjusted by different metal attenuators (1.15mm C ,1.41mm Al and 1.24mm Al). Depending on the size of the sample a stack of 4 to 6 overview scans of the hearts was recorded at an effective pixelsize of 3.58 $\mu$m. For each position, 4001 projections were recorded with 35 $\mu$s exposure time over 360 degree in continuous scan mode. Further, high resolution scans of region of interest were scanned at 0.71 $\mu$m pixel size. At least one high resolution plane ( 3x3 tomograms) through the center of the heart as well as one tomogram near the apex was acquired. In total more than 1000 tomograms were recorded.

All imaging parameters are tabulated in Tab.2. The murine hearts were stored in a Eppendorf tube filled with PBS and imaged in tomographic acquisition schemes. The acquired intensity images are empty-beam corrected, and phase retrieval was performed on each empty-beam corrected intensity using the holotomotoolbox^41^. The 3d sample volume was then reconstructed from the retrieved phase distributions via the filtered backprojection implementation of the ASTRA toolbox^42^. Ring artefacts in the tomographic reconstruction were corrected by the wavelet-based ring removal algorithm^43^. The acquisition and reconstruction parameter are listed in Tab.2. To cover the volume of the whole heart several neighboring tomograms were acquired. These single tomograms were stitched using the NRStitcher algorithm by^44^, after binning to 3.2$\upmu$m. The stitched volume was highpass filtered to remove remaining low spatial frequency ring artefacts from the different tomograms. The steps of the workflow were in the following order: ring artifact removal, binning, stitching, and finally high-pass filtering. This is now explicitly stated in the revised methods section of the MS. Overlap between scans varied for stitching, depending on configuration and size of the heart, but scans were designed such that the overlap was sufficient for stitching. For the Id19 dataset, for example the overlap was larger than 5% for the radial direction (covered by an hexagonal arrangement of tiles) and about 20% in the vertical direction between the layers. The image quality in the overlap region after stitching after the filtering scheme was verified by visual inspection.

### Segmentation and anatomical measures

The anatomical measures of the heart wall thickness *d*, the nematic order parameter *s* and the anisotropy parameter $\Omega$ were calculated for each heart to quantify the 3d structure. To this end, a workflow was developed that segments the 3d heart shape within each reconstructed volume. To orient each segmented heart in an identical coordinate frame, a standardized alignment of the hearts was developed and performed.

### Bullseye plot

Bullseye plots are an abstract spatial representation of the heart volume in a projected cylindrical coordinate system^29^. The investigated observable can then be averaged for each segment and displayed graphically. The heart is divided into 18 sections by dividing the long axis into three layers, which are then sub-divided into six azimuthal sections as visible in Fig. 2(c) & (d). The inner ring represents the apex, the middle ring the center of the ventricle and the outer ring the basal volume. The six azimuthal sections represent an anatomical direction, notably front (anterior), back (inferior), the wall dividing the ventricles (septal), and the direction pointing away from the body axis (lateral). The bullseye plots were created using the package *SmoothAHAplot*^45,46^

### Segmentation

**Fig. 3:**
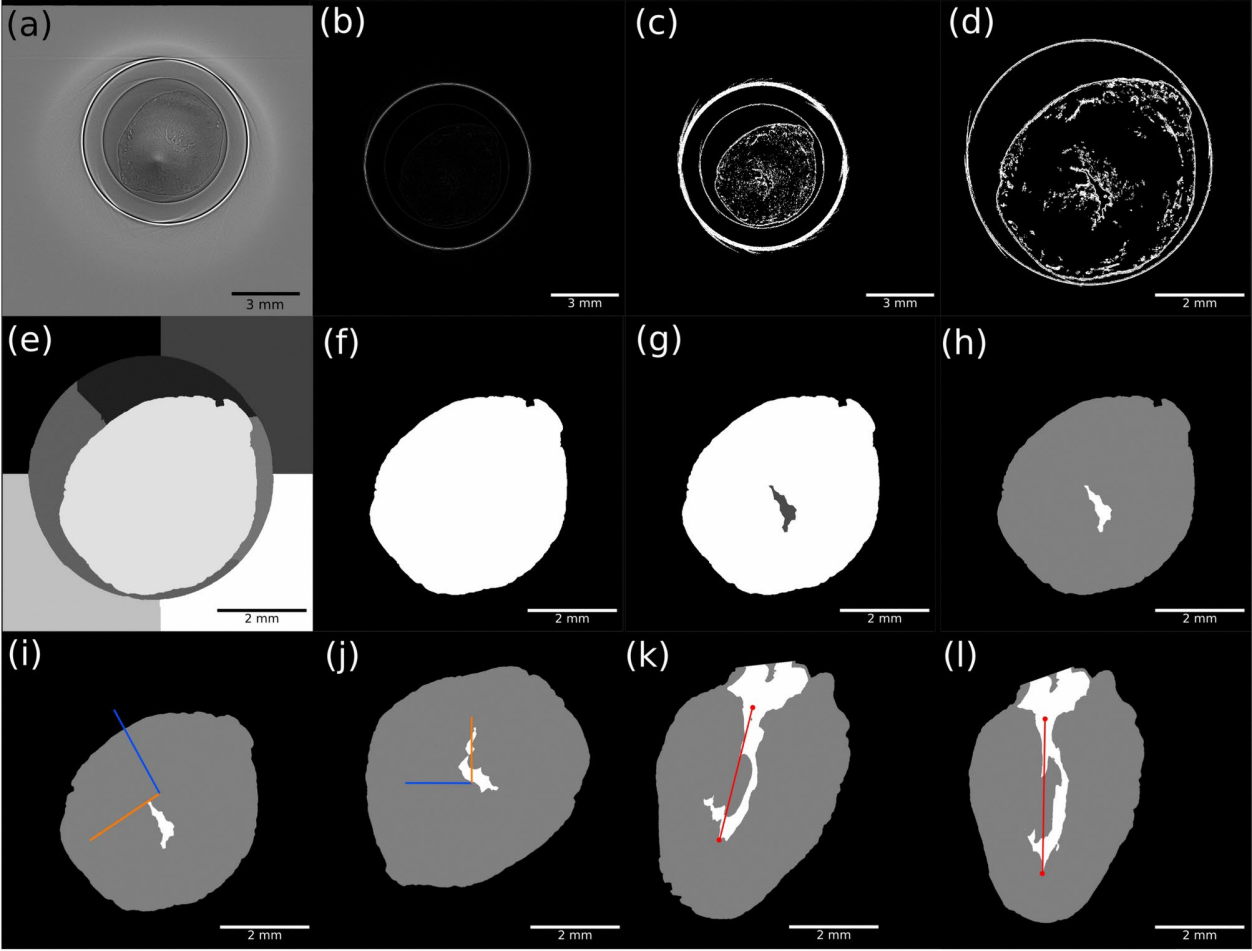
Segmenation Workflow (**a**)-(**h**) and the Rotation workflow (**i**)-(**l**). (**a**) The experimental data from the tomography as the electron density. (**b**) 3D sobel filter used on the experimental data. (**c**) a data specific threshold is used on the 3D sobel data, so that the edges of the heart are highlighted but noise is excluded and the hearts edge can have holes which will be filled later. (**d**) the image size is reduced by differentiating between not connected components in the image and taking the component with the heart and then the image borders are reduced to the edges of the chosen component. (**e**) watershed algorithm results from data created from d and blurred with a gaussian filter. (**f**) heart tissue mask from e where the tissue and the ventricles are in the mask. (**g**) the mask from f was used on the data of b and the following steps are done to get the watershed results of the heart and the ventricles. (**h**) mask from the g results where tissue has value 1 and the ventricle 2, rest 0. Rotation workflow now (**i**) The inertia tensor is caclulated and the Eigenvalues and eigenvectors are determined. The mask is shown from h with the two eigenvectors. (j) the euler angles are determined from the eigenvectors and the mask is rotated according to the Euler angles. the rotated mask is shown with the rotated eigenvectors. (**k**) side view of the rotated mask. (**l**) again rotated mask so that the tip of the ventricle is directly below the centre of mass of the ventricle.

 The 3d shape of the heart from the reconstructed 3d volume $V_{raw}$ is recovered by first applying a Sobel edge filter $\mathcal {H}_S$^47,48^, which highlights the edges within the volume $V_{sobel} = \mathcal {H}_S * V_{raw}$. Weak edges below a certain threshold $t_{sobel}$ are removed from the volume $V_{sobel}$ to obtain the edges of the heart and the surrounding Eppendorf tube. The threshold parameter was determined iteratively by eye until small residual edges due to noise disappeared. Next, different features within the volume are marked by counting all connected voxels to one component. To this end, the component corresponding to the edge of the Eppendorf tube is removed by visual inspection. The edges of the heart are now the predominant component in the volume $V_{sobel,corr}$. The interior of the heart is segmented by applying a watershed algorithm^49–52^ to the corrected volume resulting in $V_{heart} = \mathcal {T} \left[ V_{sobel,corr}\right]$. The workflow of the segmentation is summarized in Fig. 3, and detailed in the supplementary information, see Fig. S.1 -S.10 and corresponding text.

### Alignment

Each heart has to be orientated identically in order to calculate the anatomical and histological measures for the same heart regions. To this end, the heart orientation is determined by the orientation of the left ventricle. In that case, the long axis of the left ventricle corresponds to the *z*-direction of the heart coordinate system, while the short axis of the left ventricle, which points from the right ventricle the left ventricle, corresponds to the *x*-direction of the heart coordinate system. Each heart volume is then rotated in 3d so that heart coordinate system and Cartesian coordinate system are the same. To this end, the inertia tensor $\Theta$ is determined for each segmented heart (or ventricle) $V_{heart}$ and diagonalized. By solving the eigenvalue problem of the tensor, the eigenvector pointing in the direction of the ventricles long axis is determined. The Eulerian angles $\varphi _z, \varphi _y, \varphi _x$ are calculated and the volumes $V_{heart}$ are subsequently rotated $V_{align} = R_x(R_y(R_z(V_{heart}, \varphi _z), \varphi _y), \varphi _x)$.

### Wall thickness

 The heart wall thickness *d* is calculated for each transverse plane at the height *h* of the aligned heart volume $V_{align}$. To this end, the tissue cross section of the left ventricles long axis is measured in the radial direction $\gamma .$ The heart wall thickness for an exemplary slice is shown in Fig. 2(e). The bullseye plots represent the mean within the volume of the segment.

### Structure tensor

The gradient-based structure tensor *ST* is calculated for each voxel. It represents the partial derivatives of image grey values and the corresponding symmetries and principal direction within the volume. To this end, the aligned heart volume is first smoothed with a Gaussian filter $V_{\sigma } = V_{align} *\kappa _\sigma$ of standard derivation $\sigma$ in a first step to avoid the amplification of noise while taking partial derivatives. The structure tensor is then defined as:

1 $$\begin{aligned} ST = \kappa _\rho *\left( \begin{array}{ccc} \left( \partial _xV_{\sigma }\right) ^2 & \left( \partial _xV_{\sigma }\right) \left( \partial _yV_{\sigma }\right) & \left( \partial _xV_{\sigma }\right) \left( \partial _zV_{\sigma }\right) \\ \left( \partial _yV_{\sigma }\right) \left( \partial _xV_{\sigma }\right) & \left( \partial _yV_{\sigma }\right) ^2 & \left( \partial _yV_{\sigma }\right) \left( \partial _zV_{\sigma }\right) \\ \left( \partial _zV_{\sigma }\right) \left( \partial _xV_{\sigma }\right) & \left( \partial _zV_{\sigma }\right) \left( \partial _yV_{\sigma }\right) & \left( \partial _zV_{\sigma }\right) ^2 \\ \end{array}\right) ~, \end{aligned}$$

where $\partial _{x,y,z}$ denotes the partial derivatives in x-,y-,z-direction, and the convolution with a Gaussian with a standard deviation $\rho$ is carried out for each matrix coefficient. Here $\sigma =3$ voxel (or equivalently $\sigma =6$ voxel in the unbinned dataset) was used, corresponding to $9.6\mu$m. In the next step, the structure tensor *ST* is diagonalised and the eigenvalues and eigenvectors are determined. The eigenvalues are ordered such that $\lambda _1 \le \lambda _2 \le \lambda _3$. Since by construction, *ST* is symmetric and positive definite, $\lambda \in \mathbb {R}_{\ge 0}$, and the eigenvectors form an orthogonal basis, such that *S* can also be represented by an ellipsoid. The eigenvector of the smallest eigenvalue or principal axis of the structure $\vec {e}_{pa}$corresponds to the direction of the smallest gradients and therefore points in the directions of fiber-like structures. It is used to reconstruct the 3d orientation vector field of the heart muscle^7,25^.

### Nematic order parameter

 The nematic order parameter *s*can be defined as a measure to quantify, how consistent the orientation direction is within a certain ROI, as described in^53,54^. Each voxel has a principal axis $\vec {e}_{pa}$, which is the eigenvector with the smallest eigenvalue of its structure tensor *ST*. The nematic order parameter *s* is defined as the largest eigenvalue of the second rank tensor

2 $$\begin{aligned} Q_{ij} = \frac{1}{2N_{ROI}}\sum \limits _{x,y,z\vert _{ROI}}\left[ e_{pa,i}\cdot e_{pa,j} - \delta _{ij}\right] (i,j = x,y,z), \end{aligned}$$

where $N_{ROI}$ is the number of voxel within the ROI, $\sum$ denotes the sum over the ROI, ${e}_{pa,i}$ denotes the components of the principal axis $\vec {e}_{pa}$ and $\delta _{ij}$ is the Kronecker delta. In this work a ROI of 7x7x7 voxel $\times$ were used, corresponding to $11.3\mu$m.

### Anisotropy

 From the eigenvalues of *ST*, the degree of anisotropy $\Omega$is obtained according to^14^

3 $$\begin{aligned} \Omega = \sqrt{\frac{\left( \frac{1}{\lambda _1} - \frac{1}{\lambda _2}\right) ^2 + \left( \frac{1}{\lambda _2} - \frac{1}{\lambda _3}\right) ^2 + \left( \frac{1}{\lambda _3} - \frac{1}{\lambda _1}\right) ^2}{2\left( \frac{1}{\lambda _1}^2 + \frac{1}{\lambda _2}^2 + \frac{1}{\lambda _3}^2\right) }} ~. \end{aligned}$$

The quantitative 3d image analysis is detailed in the supplementary information, see Fig. S.11-S.26, and corresponding text.

## Results

### High resolution reference heart

**Fig. 4:**
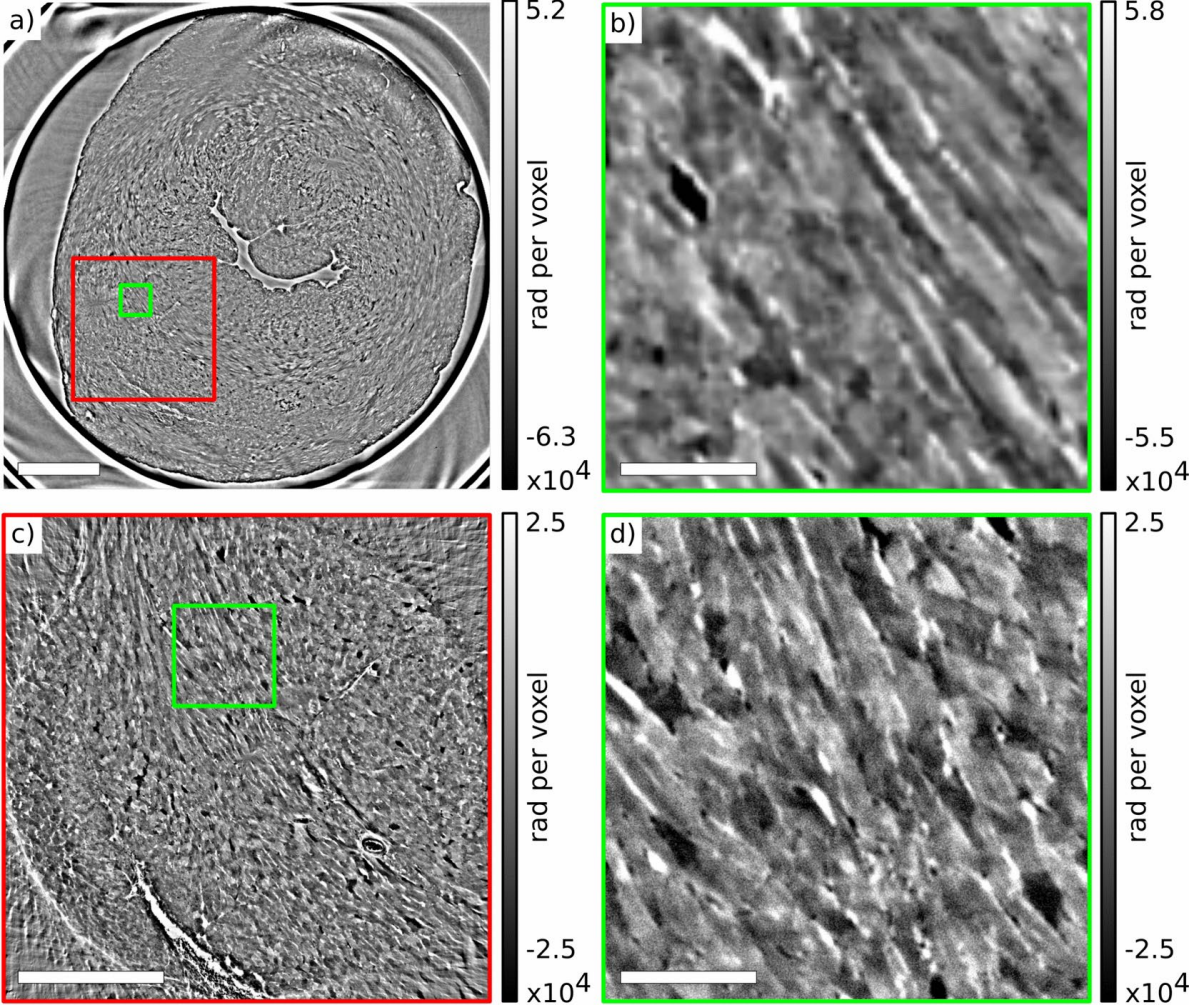
Virtual slices of the reconstructed 3d volume of a healthy murine heart in PBS, acquired at beamline ID19. (**a**) 3d reconstruction of the whole heart obtained by stitching five adjacent tomograms at a pixelsize of 6.4$\upmu$m (4x 1.6$\upmu$m binned). (**b**) Magnified view of the region marked in (**a**) by the green rectangle, revealing the orientation and structure of in-plane oriented cardiomyocytes. (**c**) Virtual slice of the tomographic reconstruction of a single tomogram taken in the highest resolution configuration at 0.65$\upmu$m pixel size. The location of the reconstructed slice within the entire heart is marked by the red rectangle in (**a**). (**d**) The green rectangle marks the same area as the green rectangle, now shown at higher resolution. Note, that the orientation is different because the sample was moved between the two measurements. Scale bars: (**a**) 1 mm, (**b**) 100$\upmu$m, (**c**) 500$\upmu$m, and (**d**) 100$\upmu$m.

The 3d volume of a control murine heart was reconstructed in two configurations at the ID19 beamline, see Tab. 2. Fig. 4 illustrates the image quality of both ID19 configurations for an exemplary virtual slice. Fig. 4(a) shows a slice through the heart which was scanned and reconstructed in its entirety in 3d by stitching adjacent tomograms at a pixel size of 1.6$\upmu$m. Image contrast and resolution are sufficient to identify single cardiomyocytes and their orientation, see Fig. 4(b). Next, adjacent tomograms were acquired in the second configuration at a pixel size of 0.65$\upmu$m. The reconstruction volume is marked by the red rectangle. The virtual transverse section shown in Fig. 4(d) illustrates the improved resolution. Single cells between the cardiomyocytes become visible. Due to the numerical challenges, all 3d analysis on the entire heart, however, was performed only on the low resolution data sets (4x binned $\approx$ 11GB).

**Fig. 5:**
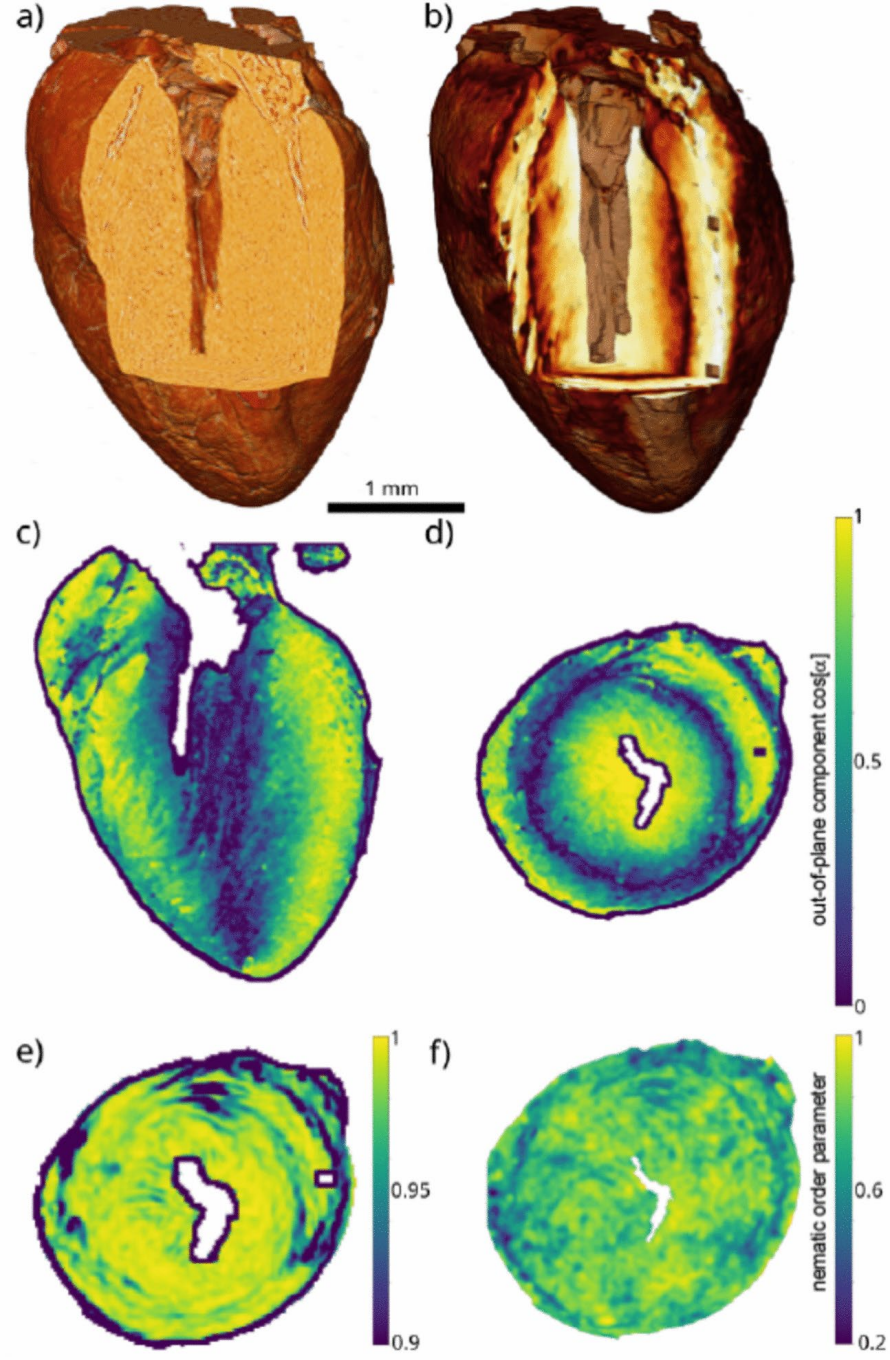
(**a**) 3d rendering of the ID19 dataset with color shading representing electron density. Values are shown for all voxels within the segmentation mask, excluding the left ventricle, but including the right ventricle which typically collapses in the excised hearts. (**b**) The corresponding 3D rendering of the structure tensor. The *z*-component of the eigenvector with the smallest eigenvalue is used for the visualisation. Lighter values represent a larger *z*-component, i.e. at a higher helical angle. The dark spots are remnants of the original image (isolated tomographic artefacts) which then appear larger after convolution with the local average ($N_{ROI}$) used in the eigenvalues analysis. Here, $N_{ROI}=$+/-6 voxel was used for the analysis of the orientational vector field. In other words, the local average over which the orientation field is determined corresponds to a cube of 13 pixel side length. (**c**,**d**) False-color representation of the quasi-vector field representing the orientation of the myocyte chains in (**c**) a long axis, and (**d**) a transverse orientation of the 2d sections. The plots show the (**c**) *y*-component, and (**d**) *z*-component of the normalized structure tensor eigenvector. In (**d**) a ring-shaped layer is observed in the middle of the myocardium with the predominant myofiber orientation in plane. (**e**) The nematic order parameter *s*, and (**f**) the anisotropy $\Omega$, shown in a representative transverse plane. Note that since the computation of *s* requires a local average, the boundary of the slice shrinks by the cross-section of this region, since values for *s* are only assigned to as points in the interior.

Fig. 5 presents a 3d rendering of the ID19 results and examples of the the structure analysis, notably visualisations of (a) electron density and (b) a component of the principal axis in the entire organ, as well as (c) & (d) the resulting orientation vector field selected planes, both long axis and transverse view. Finally, (e) nematic order parameters *s*, and (f) the anisotropy $\Omega$, are also shown by way of example in a representative transverse plane in Fig. 5. A ring-like layer can be identified, where myocyte chains are mainly oriented in the transverse plane. This layer is contained in the myocardium, while the myocardium oriented towards the endocardium and epicardium are characterized by a larger helical angle, with correspondingly larger out-of-plane component of the orientation vector.

**Fig. 6:**
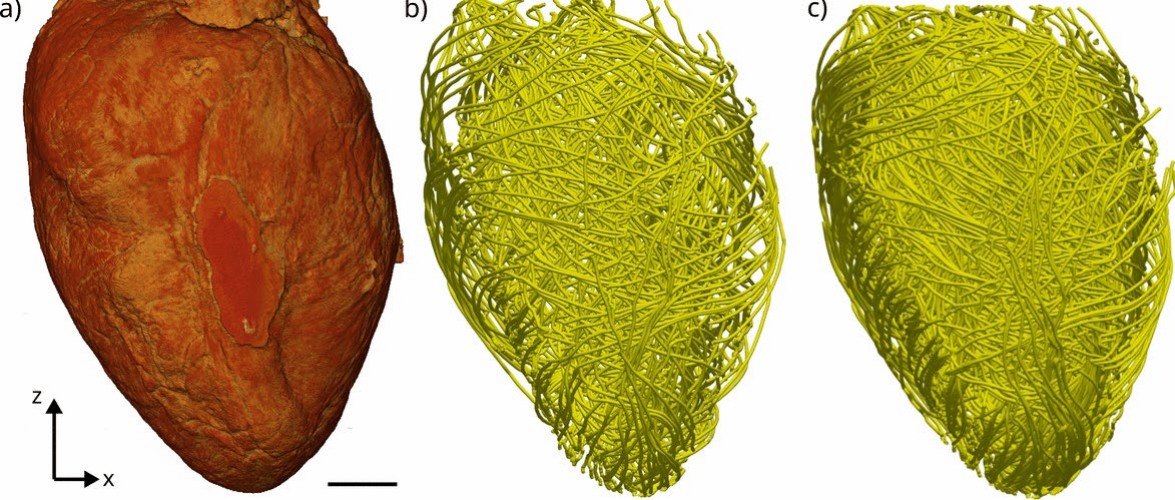
Visualisation of the reconstruction of the murine heart 3d structure obtained by X-ray phase-contrast tomography. (**a**) Surface rendering of the 3d reconstruction. Note that the flattened tissue in the center front region is slightly pressed by the wall of the plastic tube in which the heart placed during the scan. The tube has been removed by segmentation). (**b**) The orientation of the myofibers derived from the structure tensor, visualised as 3d streamlines (streamplot function of matplotlib) based on the 3D vector field of the structural orientation obtained by the structure tensor. The streamlines were generated by 5000 seedpoints in (**b**) and 1000 seedpoints in (**c**), respectively. Seedpoints were placed randomly in the volume. Scale bar: 1 mm.

Fig. 6 illustrates the 3d reconstruction, by (a) a surface rendering of the density, and (b) 3d streamline plot of the orientation vector field. Strictly speaking, this is a quasi vector field, since the sign along the orientation is unspecified. Importantly, inspection of the reconstructed density, both from the surface as from the interior confirms the intactness of the heart tissue without noticeable fissures or shrinkage artifacts, due to its embedding in phosphate buffer solution.

## Characterization of cardiac disease

**Fig. 7:**
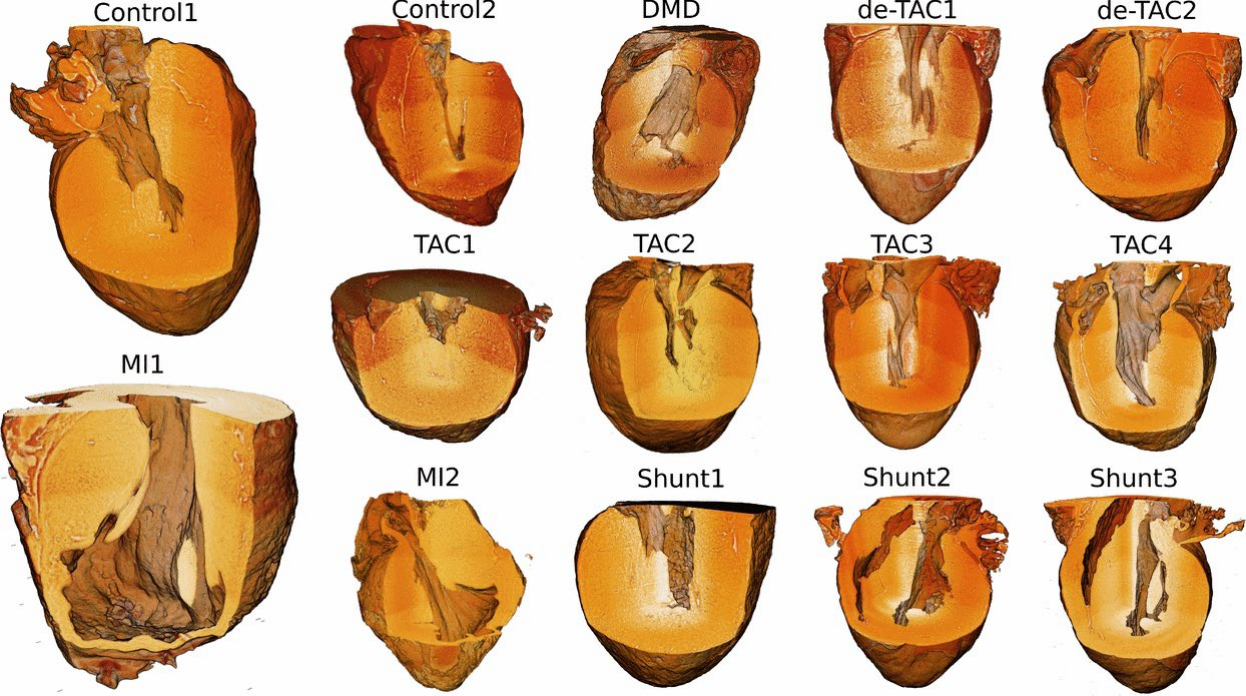
3d segmentation of the pathological series (ID17 datasets), grouped according to the respective pathology, as indicated by the corresponding labels. Two hearts (control, MI) are presented in an enlarged view. The same tabular arrangement is kept for the three results figures below. Density values for voxels within the segmentation mask are coded in false color. A cuboid is used to carve out the upper front part, in order to enable visual inspection of the interior. Figures have been created by Avizo 9.2.0 Lite, Thermo Fisher Scientific, Germany.

**Fig. 8:**
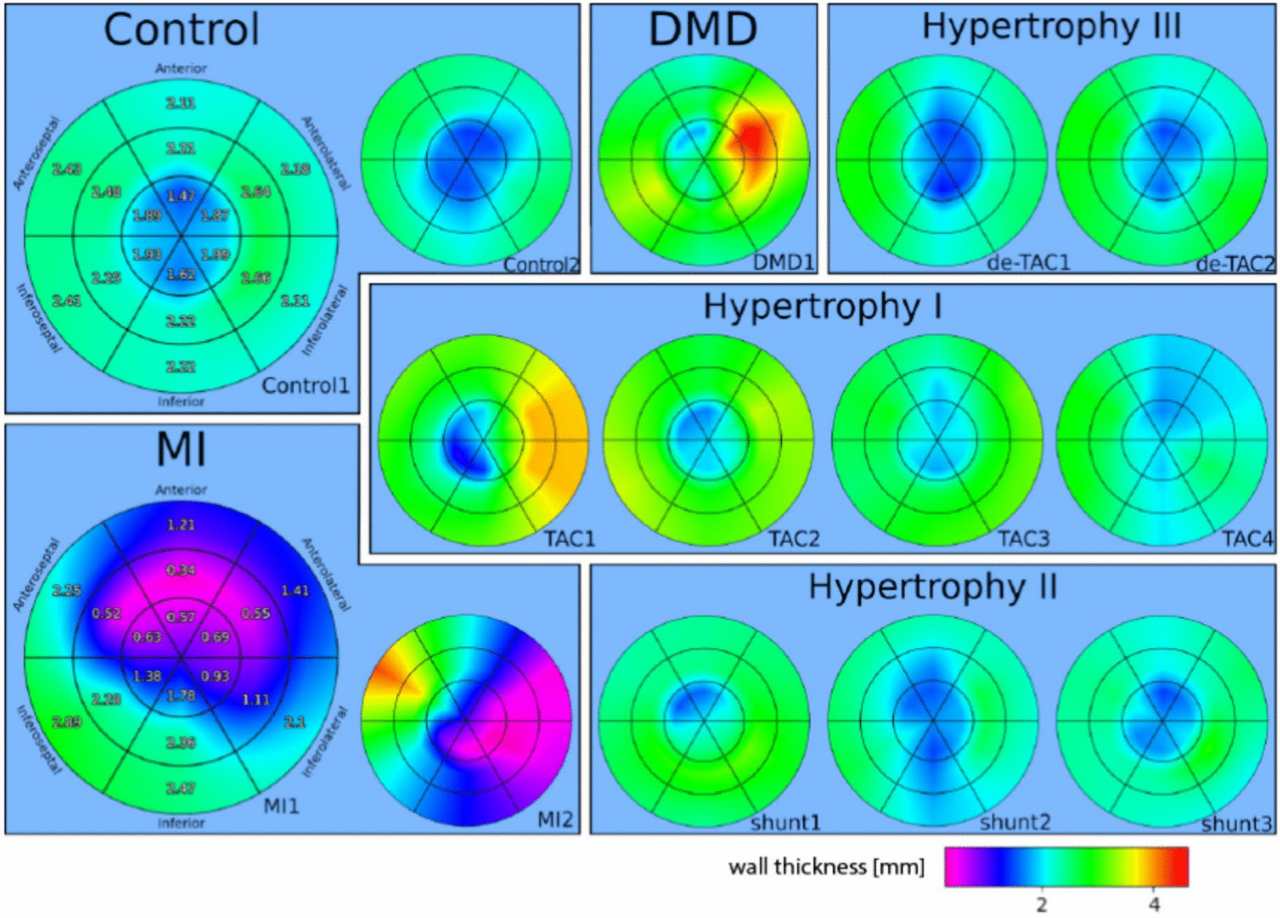
The wall thickness of all analysed hearts are shown in a bullseye plot. Bullseye plots are an abstract spatial representation of the heart volume for an observable. In a bullseye plot, the heart is divided into 18 sections by creating thirds along the long axis and each third is divided into 6 sections. Then for each sector the observable i.e. the wall thickness, is determined. The inner ring of the bullseye plot is describing the apex and then going up, so the middle ring is the mid-cavity and the outer ring represents the basal volume. Each of the sections in a third represents a spatial volume/direction in the heart. It is divided by the front (anterior), the back (inferior), the wall dividing the ventricles (septal) and the direction away from the body middle axis (lateral). The two control hearts exhibit an even wall thickness distribution in the mid and basal volume and a reduced wall thickness in the apical volume. The MI location can be determined for the two MI hearts with massively decreased wall thicknesses. The TAC hearts have an overall increased wall thickness. The bullseye plots have been created using the function bullseye of the package *SmoothAHAplot*^46^.

**Fig. 9:**
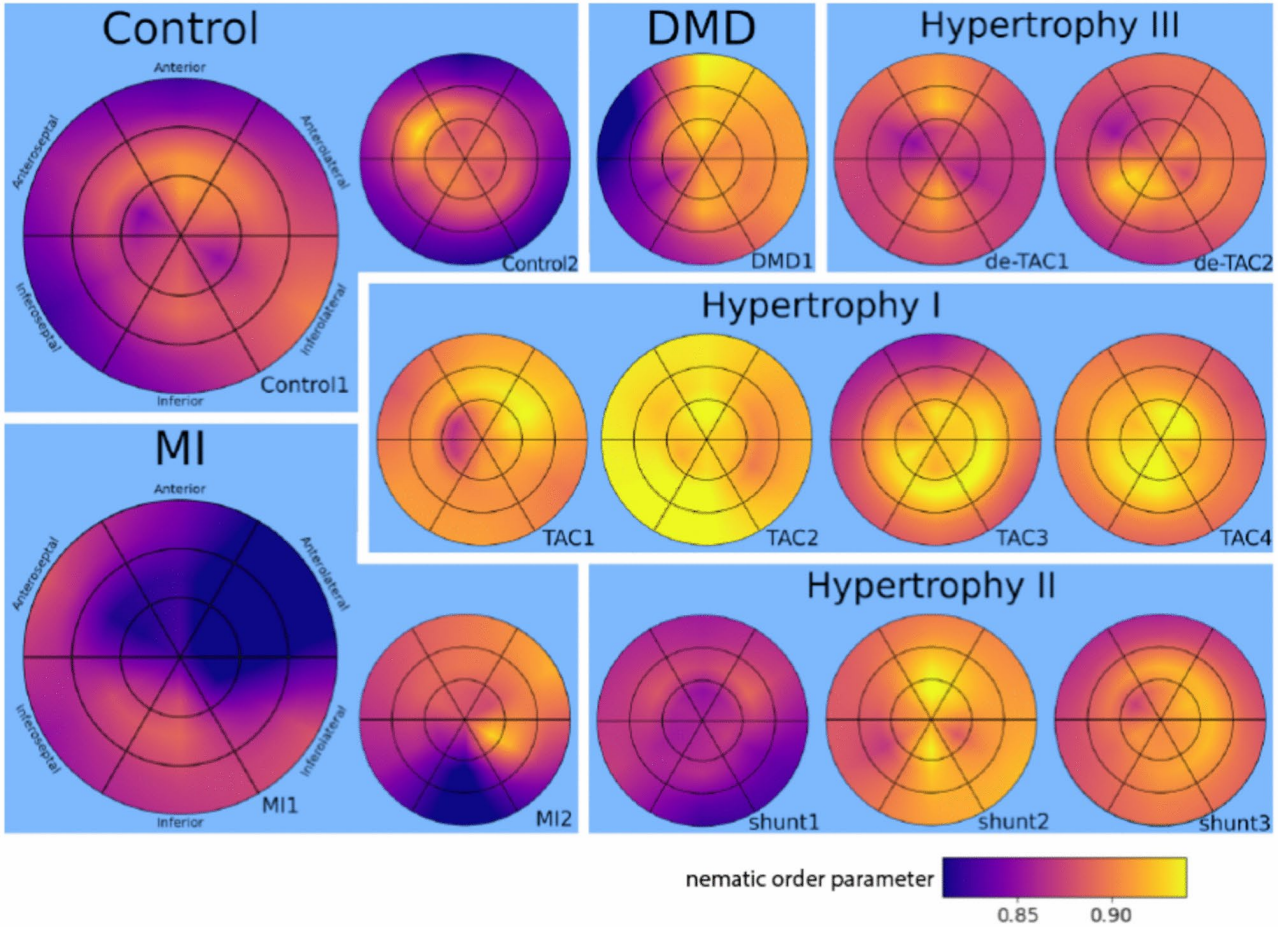
The nematic order parameter *s*, shown for all hearts in bullseye representation. The values for *s* has been computed from the structure factor with parameters $\sigma , \rho$. The bullseye plots have been created using the function bullseye of the package *SmoothAHAplot*^46^.

The pathological series contains two control samples, two hearts with MI, four hearts with TAC, three hearts with shunt, two hearts with de-TAC, and one heart with DMD. All reconstructions were segmented and rotated to obtain identical orientation, as described in Sec. 2. The segmentation allows to single out all voxels representing the heart tissue. Using this logical mask, greyscale values of the density are first visualized in 3d (Fig. 7), then evaluated in view of the ventricular wall thickness (Fig. 8) and nematic order parameter *s* (Fig. 9). While *d* is clearly an anatomical parameter, *s* and $\Omega$ can be regarded as histological morphometric parameters. As described above, they are derived from the local structure tensor, which accounts for the symmetry and values of first partial derivatives on length scales given by the voxel size of 3.6$\upmu$m. To preserve the anatomical context *d*,*s*, and $\Omega$ are evaluated for each of the 18 heart segments separately, and displayed accordingly.

Fig. 7 presents renderings for all hearts of the entire series, arranged in a table. This scheme of presentation is kept for the two figures below, reporting the morphometric parameters, wall thickness *d* and nematic order parameter *s*. The reconstructed volumes are shown in false color reflecting density values. Only voxels which lie within the segmentation mask are included. The segmentation mask excludes all of the left ventricles and also the right ventricles of some hearts if these were not too tightly collapsed. A cuboid is carved out in order to enable visual inspection also of the interior. The significantly enlarged ventricular walls associated with hypertrophic hearts are readily noticed for TAC1, TAC2, de-TAC2, and Shunt1, even without quantification. Similarily, the thinned walls around the regions of the myocardial infarction are obvious in MI1 and MI2, see lower right region of the apex. Next, we present a selection of the most important results, while all quantified parameters are presented in the supplementary information.

Fig. 8 presents the wall thickness *d* for all analysed hearts as bullseye plots in the tabular scheme introduced above. The two control hearts exhibit an even distribution in the mid and basal volume, and a reduced wall thickness in the apical region. The MI location can be determined from the massively decreased wall thickness for the two MI hearts. The hypertrophic models, in particular the TAC hearts, show an overall increased wall thickness.

Fig. 9 shows the nematic order parameter *s*, quantifying the local consistency of the orientation vector. The same bullseye plot and schematic as in Fig. 8 is used. The hypertrophy models, in particular the TAC hearts exhibit increased nematic ordering, reflecting a remodelling of the cytoarchitecture. The MI hearts exhibit decreased order in the region of the infarction. Further result graphics are included in the supplementary information, see Fig. S.27-S.42, and corresponding text.

## Discussion

The 3d reconstruction of an entire murine heart presented here (ID19 beamline), for an hydrated organ at a pixel size of 3.2$\upmu$m (2x 1.6$\upmu$m binned) represents a high quality reference dataset with contrast and image quality. No indications for radiation damage, bubble formation or object movement are observed. Contrast variation within the tissue makes it possible to outline aggregates of myocytes, at the single cell level. The difference in electron density sometimes observed between adjacent cardiac cells in their hydrated state is somewhat unexpected. It should be further investigated whether these can be partly explained by different levels of calcium or from contrast variations given by the high internal structure (myofibrills, sarcomeres) of adult cardiomyocytes. This high image quality results from a number of recent advances: first, an unprecedented brilliance and lateral coherence available after the EBS-upgrade of the ESRF even above 20keV, improvements in the beamline optics and instrumentation, as well as optimized phase retrieval and image processing. A ring-like layer in the myocardium could be clearly identified, where myofibrils are mainly oriented in the transverse plane, in contrast to the endocardium and epicardium, where the orientation vector of the myofibers has much larger out-of-plane component (higher helical angle). A complete reconstruction of the orientation (quasi-) vector field has been attempted, but it seems likely that expert groups in data science and 3d data processing will be able to improve the fiber-tracking and extract more information. Initiatives such as the Helmholtz imaging platform^55^ are ideally suited to address such tasks collaboratively. To this end, all data reconstructed and analyzed here (as a starting point) are made publicly available.

**Table 2:** Parameters of the tomographic acquisition, phase retrieval, ring removal (post processing), and stitching, listed for the two ID19 and the ID17 configurations. The X-rays are generated by an undulator (U) at ID19 and by a wiggler at ID17 (W). The phase retrieval was performed via the contrast-transfer-function (CTF) approach or the generalized Paganin method (GPM)^56^.

	ID19 conf.1	ID19 conf.2	ID17
Acquistion parameter			
Photon energy E [keV]	19 (U)	19 (U)	50 (W)
Monochromaticity$\frac{\Delta E}{E}$	10$^{-2}$	10$^{-2}$	Broad spectrum
Detector FOV [$mm^{2}$]	4.1 x 3.2	1.7 x 1.4	9.2x 2.3 (beam height)
Pixelsize [$\mu m$]	1.6078	0.6493	3.58
Source sample distance$z_1$[m]	145	145	150
Propagation distance$z_2$[mm]	270	170	1000
Fresnel number *F*	0.15	old / new 0.038	0.5169
Exposure time [ms]	100	50	35
Projections	1500	1500	4001
Angular range [$^{\circ }$]	0–180	0–180	0–360
Phase retrieval			
Method	CTF	CTF old	GPM
$\alpha _{1}$	$0.04404*10^{-4}$	$0.00069*10^{-4}$	-
$\alpha _{2}$	0.288	0.288	-
$\frac{\delta }{\beta }$	1400	1400	130
Ring removal			
Method	Wavelet	Wavelet	Wavelet
Wavelet name	sym16	sym16	sym8
DecNum	7	7	7
Sigma	4	4	3
Stitching			
Number of tomograms	20	120	4–6
Highpass filter kernel [px]	8	16	300

Using adapted XPCT workflows at SR facilities, scan times are compatible with series of hearts, scanned under identical conditions. Here, we have demonstrated the automated analysis of a small pathological series of murine heart models, namely induced myocardial infarction, and three models for heart overload and hypertrophy, as well as a model for Duchenne muscle dystrophy. Changes in the myocardial wall thickness, in local order of the myofiber orientation was observed, but has to be further corroborated by extending the number of cases. The main goal here was to create the required methodology. To this end, a segmentation pathway was presented, and a method to associate a coordinate frame with each segmented heart, based on the main axis of the inertia tensor. After rotating the reconstructions accordingly, the 18 segment based anatomical classification could be performed in order to link histological readouts to the respective anatomical region. The results were plotted in bullseye representation. While the two control hearts exhibited an even distribution in the mid and basal volume, and a reduced wall thickness in the apical region, the MI location could be clearly determined for the two MI hearts, based on reduced wall thickness along with reduced myofiber orientation. The hypertrophic models, in particular the TAC hearts, showed an overall increase in wall thickness and increased orientational order *s*.

The main limitation of the present work concerning the high resolution dataset on entire murine heart is the fact, that we could not fully exploit and process the information contained in this dataset. Due to numerical challenges, only the low resolution dataset was fully analyzed. While the imaging technique certainly surpasses classical histology, bulkiness of the data is still a major challenge. Issues such as tile reconstruction, stitching artifacts, and filtering artifacts, all reduce the throughput, as long as automated imaging protocols are not implemented. To handle the high resolution data, further improvements of the structure tensor implementation with regards to speed and accuracy are necessary. A main question is the most suitable choice associated convolutional kernels, and the corresponding dependence of the parameter histograms. One could extend the analysis to the degree of anisotropy $\Omega$ and the ST derived shape measures^25^. To this end, real-space and reciprocal space approaches could be compared^12^. Further, there is not yet enough reference data available to underpin any interpretation of the ST results, so that these remain rather descriptive, albeit in a quantitative sense. Future exploitation of the data, including segmentation of individual cardiac cells is likely, and the approach presented here can be expected to deliver datasets in which all cells of an entire heart could be segmented.

Several challenges have to be met in order to open up a clinical perspective for whole organ imaging at histological resolution. The first obvious challenge is to upscale from mouse to man. With the recently commissioned three-pole wiggler source BM18/ESRF and the advent of HIP-CT, the required beam size and photon energy can be reached also for highly coherent synchrotron radiation (SR), along with suitable imaging protocols. Yet, hydrated conditions and few micron voxel sizes still call for further improvement. This provided, once could address human heart pathologies in post-mortem entire hearts. This could serve two clinical purposes: Firstly, creating atlases of heart pathologies as reference data, and secondly, improving heart radiology by correlating clinical cardiac imaging with high resolution post-mortem reconstructions. Finally, XPCT can be translated from SR to laboratory $\mu$-CT, based on recent advances in instrumentation and image processing. Again SR results can serve as ground truth and to train convolutional neuronal networks (CNN) or other forms of artificial intelligence (AI), which are then used to process $\mu$-CT or even clinical radiology data. In this way, high resolution imaging could benefit both clinical radiology and pathology, in the long term. Note, however, that presently XPCT is based on electron density distribution and still lacks the capability of marking specific biological structures of interest. Development of specific X-ray contrast agents compatible with tissue and whole organ protocols therefore remains an important goal for future work.

## Supplementary Information

Supplementary Information.

## Data Availability

The reconstructed data and the analysis scripts are publically available at [https://zenodo.org/records/14501369]. The ESRF raw data is available under DOI 10.15151/ESRF-ES-956648295.

## Declarations

### Use of experimental animal

Research has been carried out according to the ARRIVE guidelines, where applicable.
