# Supplementary material for: Three-dimensional structure of entire hydrated murine hearts at histological resolution
Source: Sci Rep. 2025 Jan 22;15:2766. doi: 10.1038/s41598-024-83853-y (PMC11754897; doi:10.1038/s41598-024-83853-y)
Supplement: Supplementary file 1 — Supplementary Information. [file 41598_2024_83853_MOESM1_ESM.pdf]

Supplementary Information

Image processing for: "Three-dimensional structure of entire hydrated murine hearts at histological resolution", Frohn et al.

Supplement authored by

Frederik Böddeker

based on a master thesis at the Institute for X-ray Physics, University of Göttingen

compiled as SM: 15th December 2024

supervised by: Marius Reichardt, Tim Salditt

Contents

S. Image segmentation of murine hearts and structure analysis 1

S.1. Tests of conventional segmentation methods 1

S.1.1. Segmentation of the heart tissue 7

S.1.2. Segmentation of the ventricles 8

S.1.3. Final segmentation masks 12

S.1.4. Preparation of the 18 segment model 17

S.1.5. Wall thickness 21

S.1.6. Volumes 28

S.1.7. Shape measures 33

S.1.8. Nematic order parameter 44

S. Image segmentation of murine hearts and structure analysis

In this chapter, first, the results from the different approaches to develop a workflow which were investigated in this thesis are presented. Then, the results of the applied segmentation workflow and the statistical analysis are shown.

S.1. Tests of conventional segmentation methods

The investigated rhesus macaque heart was used to create the preliminary workflows due to reduced noise and fragments in the recorded data compared to the murine hearts. It was measured and reconstructed in the in-house experimental setup *easy-TOM*. The single rhesus macaque heart image was used to test the first applied segmentation methods. While the rhesus macaque heart was used to implement the here developed workflow, the mice hearts were used for the analysis procedures performed in the course of this thesis.

ilastik The first attempt using *ilastik* was also the a first try to use segmentation methods on the data. While the data appeared to be suitable for machine learning basesd classification, the software had to be iterated and with each iteration more complex filters had to be applied.

In figure S.1, the results of the first attempt at segmenting with *ilastik* is presented, with the initial image in figure S.1(a) and the segmented image in figure S.1(b). Parts of the image were labelled as either heart tissue or background. The outline of the heart and the ventricles are captured, but the segmentation overestimates the size of the ventricle in the middle of the image and the edge of the heart to the background is not accurately segmented. Especially the areas around the dark fragments, which is most likely fat, in the heart, in figure S.1(a), is not classified as heart tissue. Furthermore, background noise, that is misclassified as cardiac tissue,

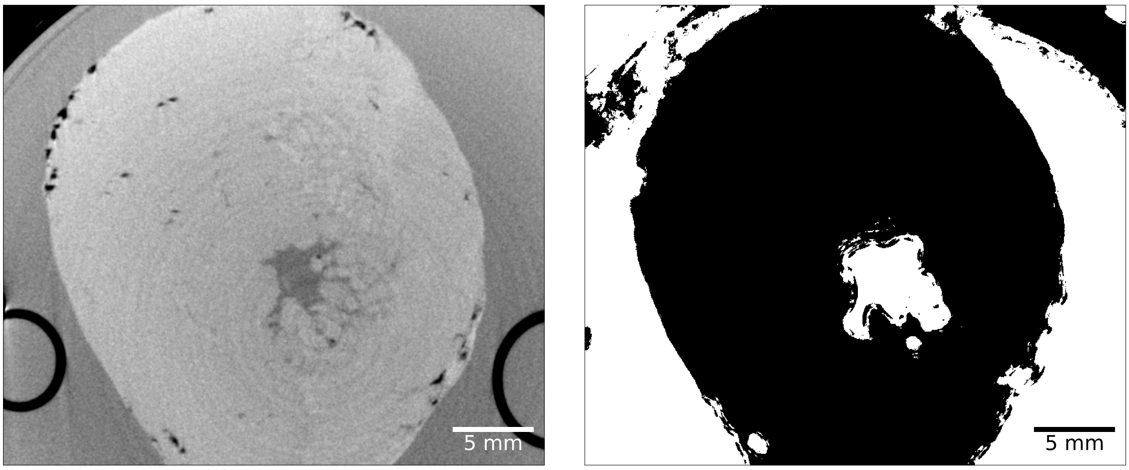

(a) Croppped image of the heart.

(b) Binary mask of the heart, segmented with *ilastik*, with the heart tissue in black and other areas in white.

Figure S.1.: An early attempt of applying *ilastik*. There are only a few slices of the heart labelled into heart tissue and other voxels, as well as there are no filters applied. (b): The general outline of the heart is visible, but the edge of the heart is not precise and the size of the left ventricle is overestimated.

is connected to the heart's basal volume. More precise labelling of the images and the introduction of filters is necessary to enhance the machine learning.

In figure S.2, the results of a more advanced attempt are shown with the initial image in figure S.2(a) and the segmented image in figure S.2(b). The heart's edge to the background is more pronounced and the segmented edge aligns better than in the first attempt shown in figure S.1. The segmented ventricle, in figure S.2(b), is still larger than the ventricle in the initial image shown in figure S.2(a). Furthermore, complications when using the software occurred. With each iteration, the computational time increased due to more complex filters, filter optimisations or a larger sample size of training data.

The results from ilastik are not bad, on the one hand, better segmentation would lead to more precise results later and on the other hand post-processing after the segmentation is required. The software is easy to use, but the results were not to the required precision. However, the results from *ilastik* could be used as reference to evaluate segmentations. In the following, other segmentation methods are explored which are either faster or provide better results.

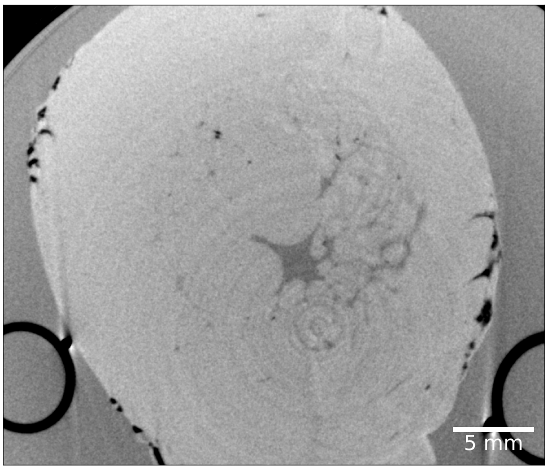

(a) Reconstructed and croppped image of the rhesus macaque heart.

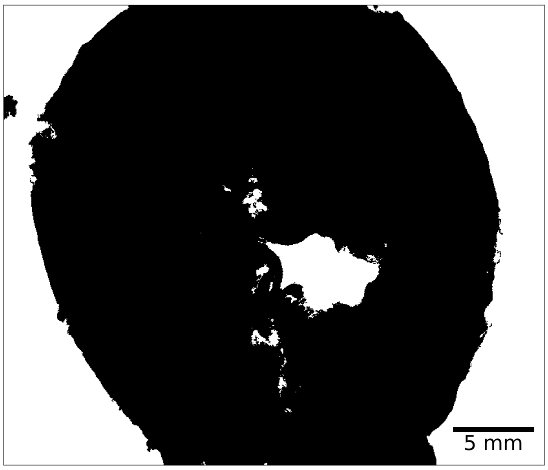

(b) Binary mask of the heart, segmented with ilastik, with the heart tissue in black and other areas in white.

Figure S.2.: Final attempt at using *ilastik* to segment the hearts. (b): The outline of the heart is more pronounced than in the early attempt, displayed in figure S.1, but the edge is misclassified around the dark fragments at the edge of the heart. Additionally, the left ventricle is still overestimated.

Basic thresholding For the basic thresholding, the Otsu threshold was calculated and applied. Figure S.3 shows the results. In figure S.3(a), an image slice of the heart is shown with a ventricle and two supports. The Otsu threshold $q_{\mathrm{Otsu}} = 76$ is calculated and the result is presented in figure S.3(b). As the black background dominates the image, the calculated Otsu threshold just divides between the black background and the heart in the sample container. Therefore, a second Otsu threshold $q_{\mathrm{Otsu}} = 148$ was calculated from the image which is masked by the binary image of the first Otsu threshold, in figure S.3(b). The result of the second thresholding is shown in figure S.3(c). While, in figure S.3(c), the general outline of the heart is visible, the heart is not isolated from the thresholded voxel around the edge of the sample container. More importantly, the used method just gives the general outline of the heart. The heart's edge is not precisely segmented, as the thresholded voxels do not create a consisting edge, and the ventricle is not segmented at all. Due to the decrease of voxel value towards the centre of the heart, a beam hardening artefact, the voxels' values are below the Otsu threshold. A more precise method to determine the heart's edges and to detect the ventricles is needed that is not only a function of the grey scale value of each voxel.

The first two attempts, using *ilastik* and thresholding, failed due to the data be-

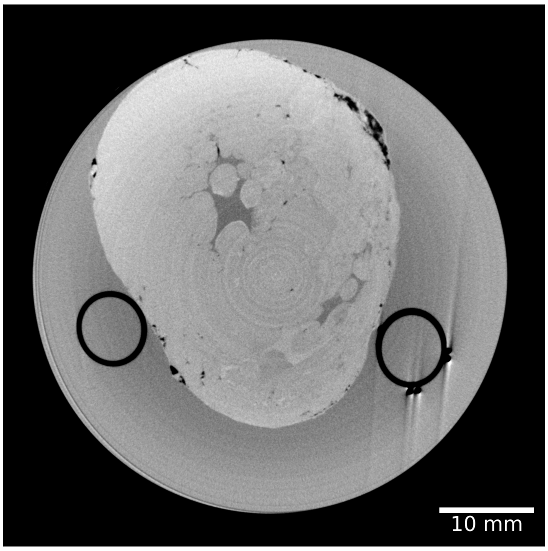

(a) Reconstructed image of the rhesus macaque heart

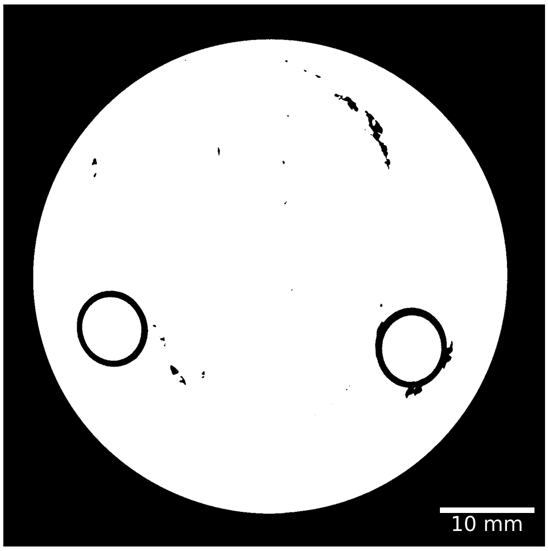

(b) Binary mask after applying the Otsu threshold onto the heart image. The Otsu threshold $q_{\text{Otsu}} = 76$.

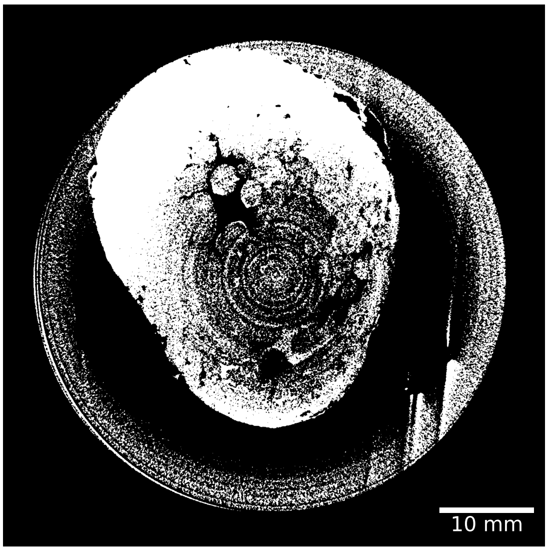

(c) Binary mask after applying the Otsu threshold onto the Otsu threshold heart image. The Otsu threshold $q_{\text{Otsu}} = 76$.

Figure S.3.: (a): The Otsu thresholded was applied onto the rhesus macaque's heart image. (b): The result led to a mask differentiating between the black background and the recorded image in grey to white. (c): A second Otsu threshold, applied onto the image data in the binary mask (white), roughly divides between the heart and the background, but there is no distinct edge of the heart and the heart overlaps with misclassified areas of the edge of the recorded volume.

ing noisy, low contrast between the heart and the background as well as artefacts as beam hardening, which have an influence on the voxel values. The two applied attempts mainly focused on the grey scale value of the voxel. *ilastik* uses filters, but the grey scale images are still an input for the software. An approach directed towards the heart's edges, which are the high contrast areas, revealed to be necessary. Successful segmentation of the edges could function as a dividing border between the heart and the background. Likewise, the murine heart data is Gaussian filtered multiple times during the reconstruction. Therefore, the murine hearts' image grey scale is equalised except for local areas where the contrast between voxels is high. Therefore, gray scale value-based methods would also not be sufficient with the already explored methods.

Canny edge detection The canny algorithm was used as a first application of an edge detector, because it singles out the edges of objects. It is less deteriorated by noise in the image due to the threshold hysteresis. It is a combination of the already described Sobel edge filter and hysteresis thresholding. An advantage of the canny algorithm is that it is already pre-implemented [**?**, skimage.feature.canny]. However, the implementation is limited to 2D images. Image slices for each dimension of the image are shown in figures S.4(a) and S.4(c). The results of a stack of 2D segmented image slices are shown in the figure S.4(b) and S.4(d) with the stack along the z-axis. It provides great results for 2D image slices as shown in figure S.4(d). The segmented edges are satisfying for one direction, but in the other dimensions, the edges do not align and better solutions are needed as shown in figure S.4(b), where the edges are not aligned. An attempt at extending the canny algorithm to 3D images failed due to an exponentially increased computational time and the results from the 2D solution could not be recreated.
However, the results indicate that a gradient-based segmentation can be a reasonable solution to segment the murine hearts.

Segmentation of murine hearts

The results of the heart segmentation workflow will be shown on the basis of a murine heart, sample MI2, and usually the same slice will be displayed to enhance the comprehensibility. The shown murine heart has had a myocardial infarction. All given voxel values are in the range of [0, 255].

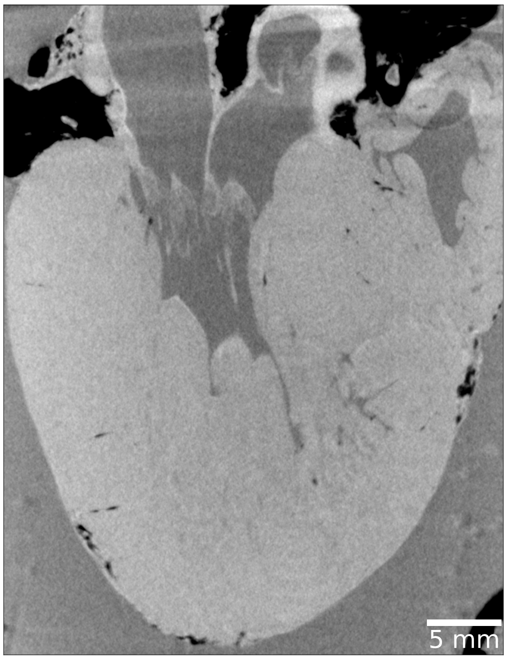

(a) View along the x-axis of the cropped heart image.

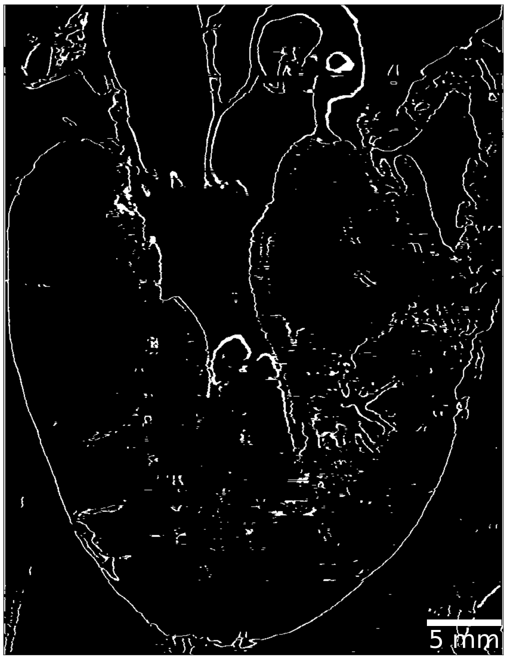

(b) Results of the 2D stacked Canny algorithm (x-axis view).

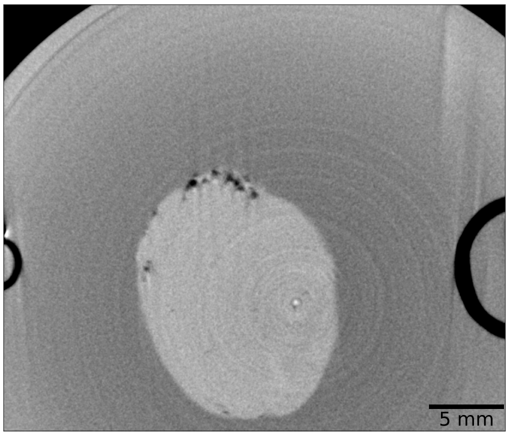

(c) View along the z-axis of the cropped heart image.

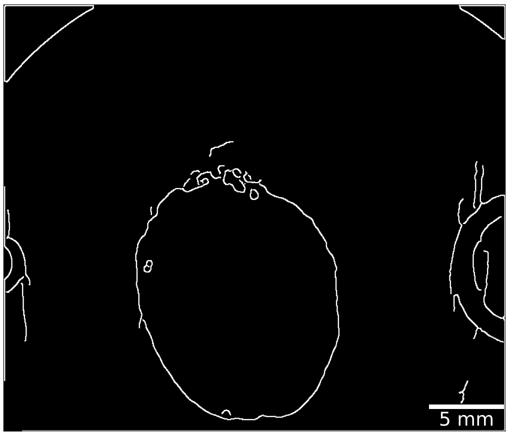

(d) Results of the 2D stacked Canny algorithm (z-axis view).

Figure S.4.: The Canny algorithm was applied on the heart image. (a,c): Images of the heart gray value reconstructions of example slices, along the x and z axis, reciprocely. The algorithm was used on each image slice independently, and the results were then stacked. The results show that the edges between different slices do not align and a 3D extension of the Canny algorithm would be required.

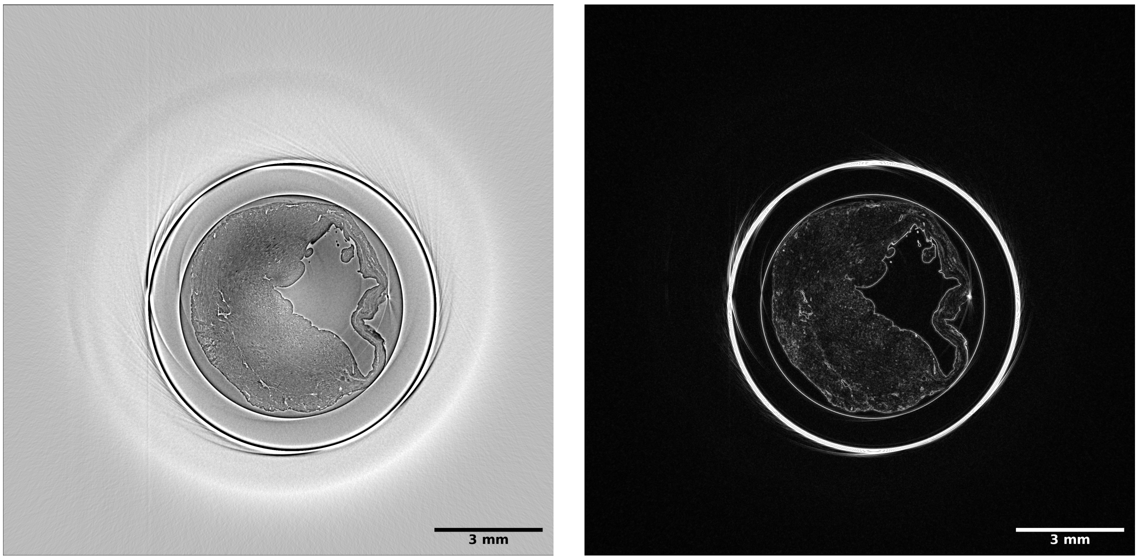

(a) Reconstructed image I_{heart} of a murine heart.

(b) Sobel edge filtered image E_I of the murine heart.

Figure S.5.: (a) The reconstructed image I_{heart} and (b) the Sobel edge filtered image E_I of a murine heart are shown. The workflow is shown by the means of a single murine heart's slice.

S.1.1. Segmentation of the heart tissue

Edge detection The self-implemented 3D Sobel edge filtering was performed without complications on the heart images I_{heart}, shown in figure S.5(a). The resulting magnitude E_I is shown in figure S.5(b). The edges of the heart are clearly visible, but the container has, as expected, a more prominent edge. As long as the container and other artefacts are not interfering with the heart's edge, the artefacts can be ignored or they are omitted during the nearest-neighbour labelling. If the artefacts are connected to the heart's edge, they will be targeted during the thresholding and the application of the watershed algorithm through the choice of appropriate parameters.

Processing The basic thresholding $T(E_I, q)$ led to a reduction in background noise, and it equalised different parts of the heart's edge. The chosen threshold of $q = 2.5$ is lower than the magnitude E_I's Otsu threshold $q_{\mathrm{Otsu}} = 37.35$, as the Otsu threshold q_{Otsu} tends to segment the sample container. The grey scale ranges from 0 to 255. The result of the thresholding $T(E_I, q)$ is shown in figure S.6(a). The heart tissue is visible while the buffer solution in the ventricles and around

the heart is almost non-visible. The threshold q was chosen so low, in order to not only highlight the edges, but also the heart tissue surrounding the edges to close gaps in the edge and widen the edge to create better results during the watershed algorithm. Additional voxels around the edges help the watershed algorithm to fill out the heart. Further, the lower threshold value allows the closing of the edges of low contrast areas. After the simplification of the magnitude E_I with thresholding $T(E_I, q)$, nearest-neighbour labelling is applied with the described connectivity condition CC_{nn}, section **??**, to remove the container and other artefacts as shown in figure S.6(b). The image $I_{\text{bin,label}}$'s size is reduced to the most outlying borders of the ROI, which is shown in figure S.6(c)

Segmentation The heart's edge is enlarged through dilation $f_{\text{di}}(I_{\text{bin,label}}, k_{\text{ball}})$ with a ball-shaped kernel k_{ball} with a radius r_k between usually $2-5$ voxels. Further, the image is prepared for the watershed algorithm with the Gaussian filter I_{G} to create a smooth surface on which the watershed algorithm can work. The results of the watershed algorithm are shown in figure S.7(a). The different areas are manually combined into binary masks of background (value - 0) and the heart (value - 1). The resulting binary mask I_{seg} is shown in figure S.7(b). The used segmentation parameters for each heart are given in table S.1.

S.1.2. Segmentation of the ventricles

The ventricles are segmented accordingly, if the basic heart segmentation did not provide the ventricles. Either the same threshold parameters q are taken again or the threshold parameter q_{ven} is lowered to emphasise the edges around the ventricles. Furthermore, the amount of information is reduced by multiplying the acquired mask I_{seg} with the Sobel edge filtered images E_I. This reduces the image size and eliminates noise, where the watershed algorithm could only provide unsatisfying results. Hence, the same processing steps as during the basic heart segmentation are followed. The image is nearest-neighbour labelled and image size reduced. Afterwards, in the course of the segmentation step, the results are dilated and Gaussian filtered before the watershed algorithm is applied. The watershed algorithm provides segmented images, as shown in figure S.8(a), and the ventricles are added to the mask I_{seg} as further values (left ventricle - 2 and right ventricle - 3). The larger ventricle in the heart and in the heart images I_{heart} is the left ventricle. All investigated hearts have their left ventricles segmented and if possible the right ventricle.

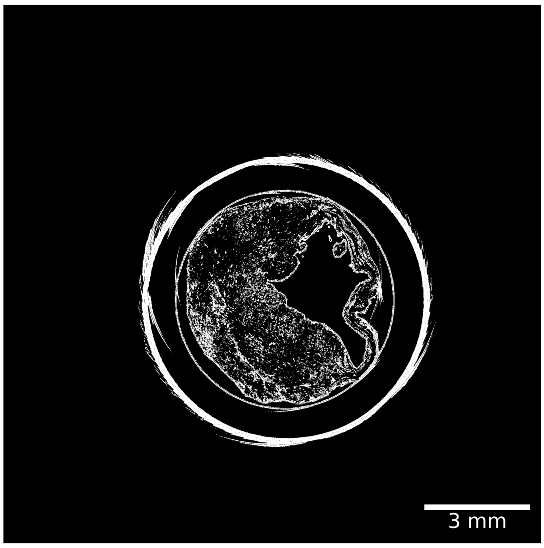

(a) Thresholded 3D Sobel edge filtered image I_{bin} of the murine heart.

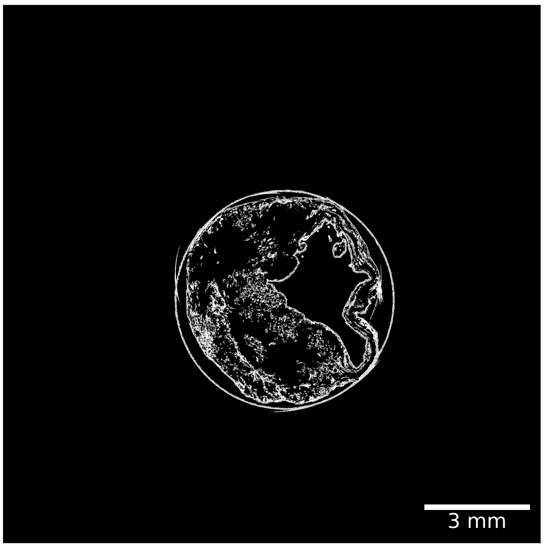

(b) Nearest-neighbour labelling image of the thresholded heart imageI_{bin}.

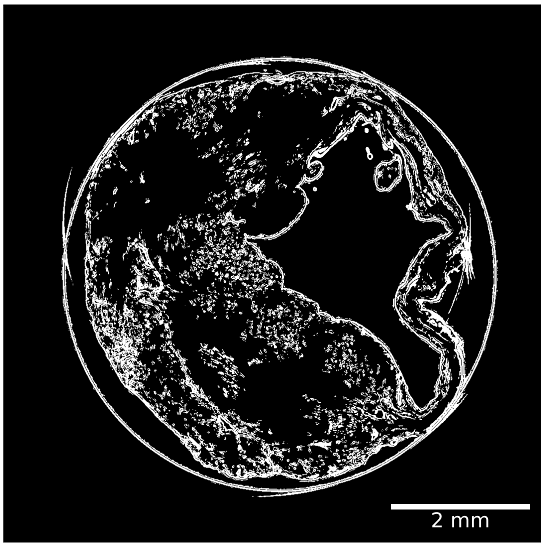

(c) Image size reduction of the binary image $I_{\text{bin,label}}$.

Figure S.6.: The processsing steps to prepare the heart image for the segmentation step. (a) Thresholded 3D Sobel edge filtered image I_{bin} of the murine heart. The chosen threshold $q = 2.5$ allows highlighting the edge of the heart towards the background and the ventricles. Further, it emphasise the heart tissue. (b) Nearest-neighbour labelling and (c) image size reduction of the thresholded heart image I_{bin}, figure S.6(a).

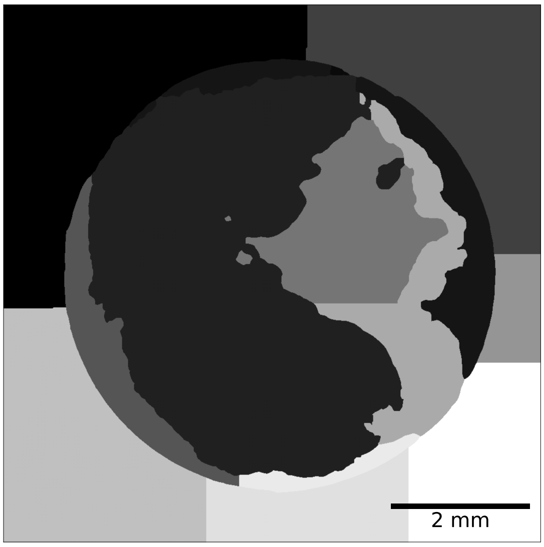

(a) Results of the watershed algorithm with the segmented heart in grey and the over-segmentation of the background in colours between black and white.

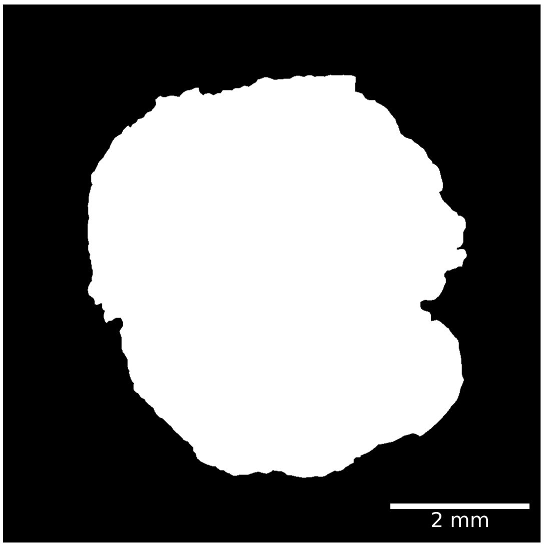

(b) Binary mask I_{seg} of the segmented murine heart with the heart and the ventricles in white and the background in black.

Figure S.7.: The results of the (a) watershed algorithm and the (b) resulting binary mask are shown. (a): The over-segmentation of the heart image is eliminated by fusing the background.

Table S.1.: The parameters used for the heart tissue segmentation. The grey scale of the data was 0-255.

sample	threshold q	image size	kernel radius r_k [voxel]	standard deviation σ [voxel]	# of markers
DMD1	1.5	$(400, 1614)_x$ $(446, 1655)_y$ $(0, 1326)_z$	3	3	40
TAC1	2	$(197, 1604)_x$ $(164, 1549)_y$ $(0, 1370)_z$	3	3	40
Control1	2	$(508, 1518)_x$ $(449, 1454)_y$ $(0, 1122)_z$	3	3	20
MI1	0.5	$(348, 1596)_x$ $(257, 1499)_y$ $(0, 1053)_z$	3	3	80
MI2	2.5	$(629, 1709)_x$ $(564, 1643)_y$ $(0, 1149)_z$	3	3	20
Control2	3	$(515, 1501)_x$ $(377, 1347)_y$ $(0, 1117)_z$	3	3	30
TAC2	1	$(317, 1818)_x$ $(344, 1802)_y$ $(0, 1455)_z$	3	3	20
TAC3	0.8	$(361, 1447)_x$ $(457, 1539)_y$ $(0, 1260)_z$	3	3	40
TAC4	0.8	$(468, 1583)_x$ $(467, 1582)_y$ $(0, 1450)_z$	3	3	40
de-TAC1	2	$(297, 1439)_x$ $(343, 1528)_y$ $(0, 1015)_z$	3	3	40
de-TAC2	4	$(421, 1368)_x$ $(398, 1349)_y$ $(0, 1156)_z$	3	3	20
Shunt1	0.8	$(364, 1493)_x$ $(379, 1508)_y$ $(0, 1455)_z$	3	3	20
Shunt2	3	$(290, 1817)_x$ $(154, 1639)_y$ $(0, 1488)_z$	3	3	40
Shunt3	2	$(359, 1839)_x$ $(160, 1660)_y$ $(0, 1526)_z$	3	3	30
Unknown1	3	$(186, 1514)_x$ $(174, 1471)_y$ $(0, 1266)_z$	3	3	30

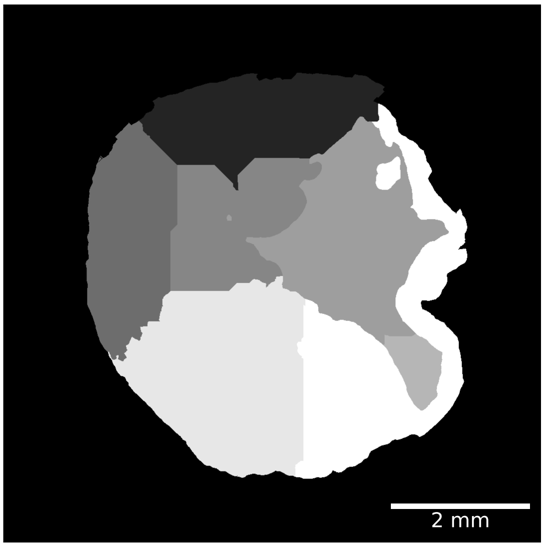

(a) The results of the watershed algorithm with the heart tissue, the left ventricle and the background.

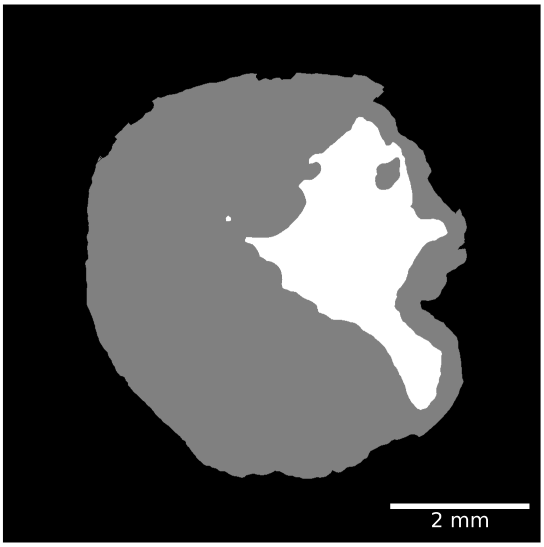

(b) The mask which is created from the results of the watershed algorithm.

Figure S.8.: The results of the watershed algorithm (a) and the resulting mask (b). In the mask, the background is shown in black, the heart tissue in grey and the left ventricle in white.

The used parameters to initialise the ventricle segmentation are given in table S.2. The given parameters are sometimes only a guideline, because the segmented ventricles are a result of multiple segmentations which were multiplied. The resulting masks I_{mask} are shown in figure S.8(b).

S.1.3. Final segmentation masks

The masks are applied to the experimental data to evaluate the segmentation. A selection of the hearts are displayed from the outside and with one section cut out of the heart wall as a 3D volume rendering to highlight comparisons and differences between the different investigated diseases. The heart rendering images are shown in figure S.9. Additionally, a slice of each heart mask I_{mask} are displayed in figure S.10. The MI sample has a reduced wall thickness, especially around the infarct region in the bottom right corner of the figure. The heart wall shrank compared to the control sample Control2 and the heart wall of the hypertrophy sample TAC2 is enlarged compared to the control sample Control2. In both pathological examples,

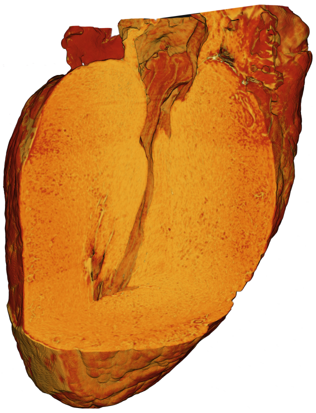

(a) 3D Volume rendering of the control sample Control2 with a cut out to show the ventricle.

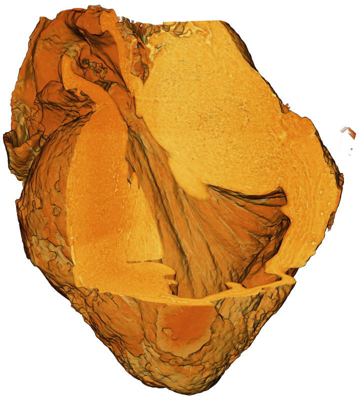

(b) 3D Volume rendering of the sample MI2 with an MI and a cut-out to show the ventricle and the position of the MI.

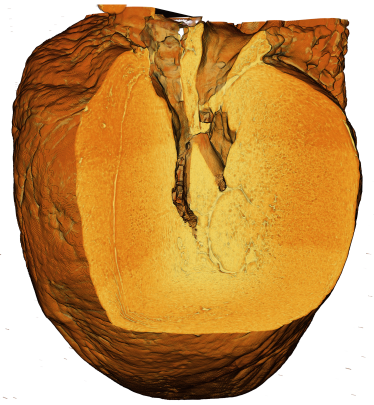

(c) 3D Volume rendering of the sample TAC2 with hypertrophy and a cut-out to show the ventricle and the enlargement of the muscle tissue.

Figure S.9.: The 3D volume rendering images of (a) the control sample Control2, (b) the MI sample MI2 and the hypertrophy sample TAC2. There is a cut-out in each image to show the ventricles and the changes to the muscle tissue for the different pathologies.

Table S.2.: The parameters used for the ventricle segmentation. The grey scale of the data was 0-255.

sample	threshold q	kernel radius r_k [voxel]	standard deviation σ [voxel]	# of markers
Control1	1.9	2	2	40
MI2	3.5	2	2	20
Control2	3.5	2	3	20
TAC2	0.7	2	2	70
TAC3	0.8	2	2	20
de-TAC1	2.1	2	3	60
de-TAC2	2.2	3	1	120
Shunt2	2.5	3	4	70
Unknown1	3.5	2	3	20

the size of the ventricle itself changes accordingly. The ventricle has a higher volume in the infarct region, and it is compressed in the case of the hypertrophy sample.

The two MI samples MI1 and MI2 can be distinguished from the other heart by eye. Their left ventricles are enlarged and one region of the cardiac wall is thinner than the cardiac walls of the other samples. The sample of the three hypertrophy models cant be differentiated through their enlarged cardiac walls. Possible *in depth* differences will be discussed during the statistical analysis.

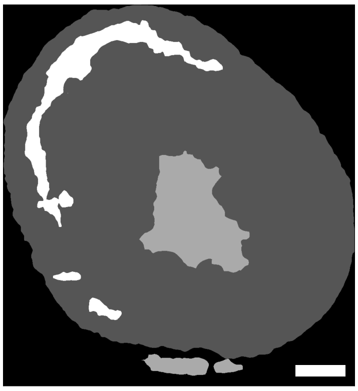

(a) DMD1

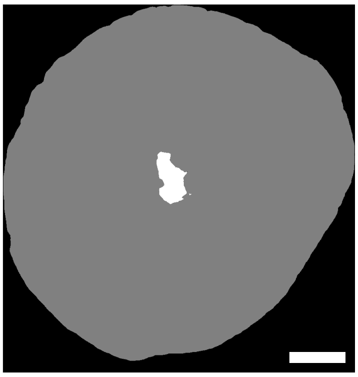

(b) TAC1

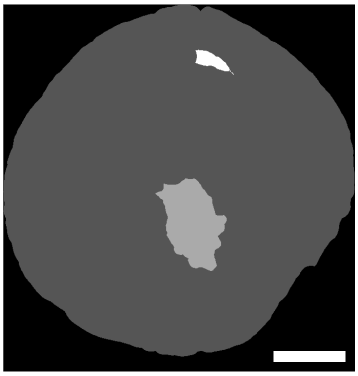

(c) Control1

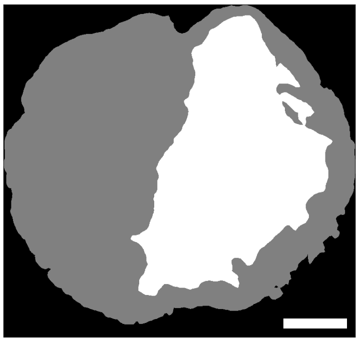

(d) MI1

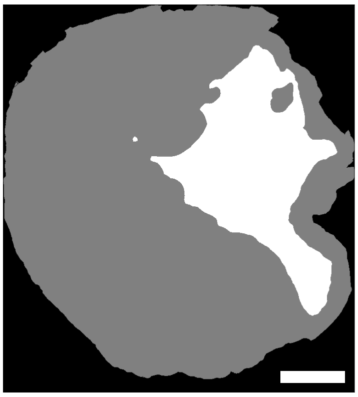

(e) MI2

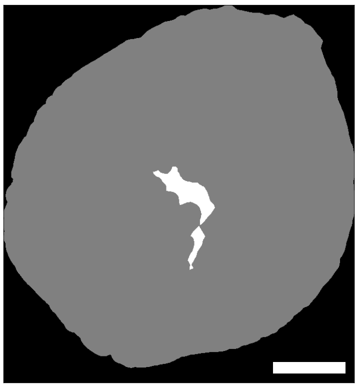

(f) Control2

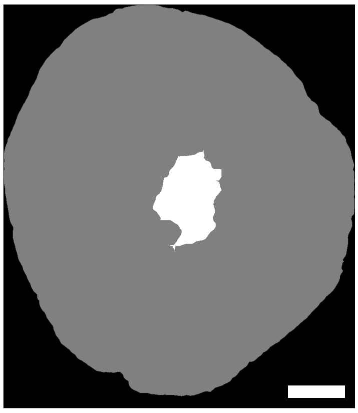

(g) TAC2

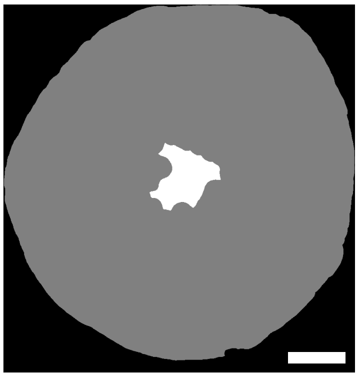

(h) TAC3

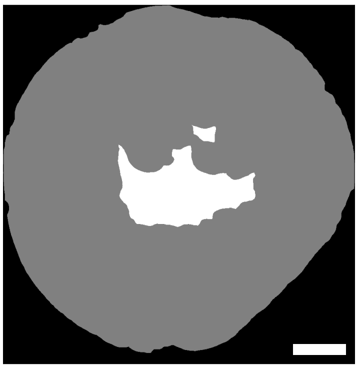

(i) TAC4

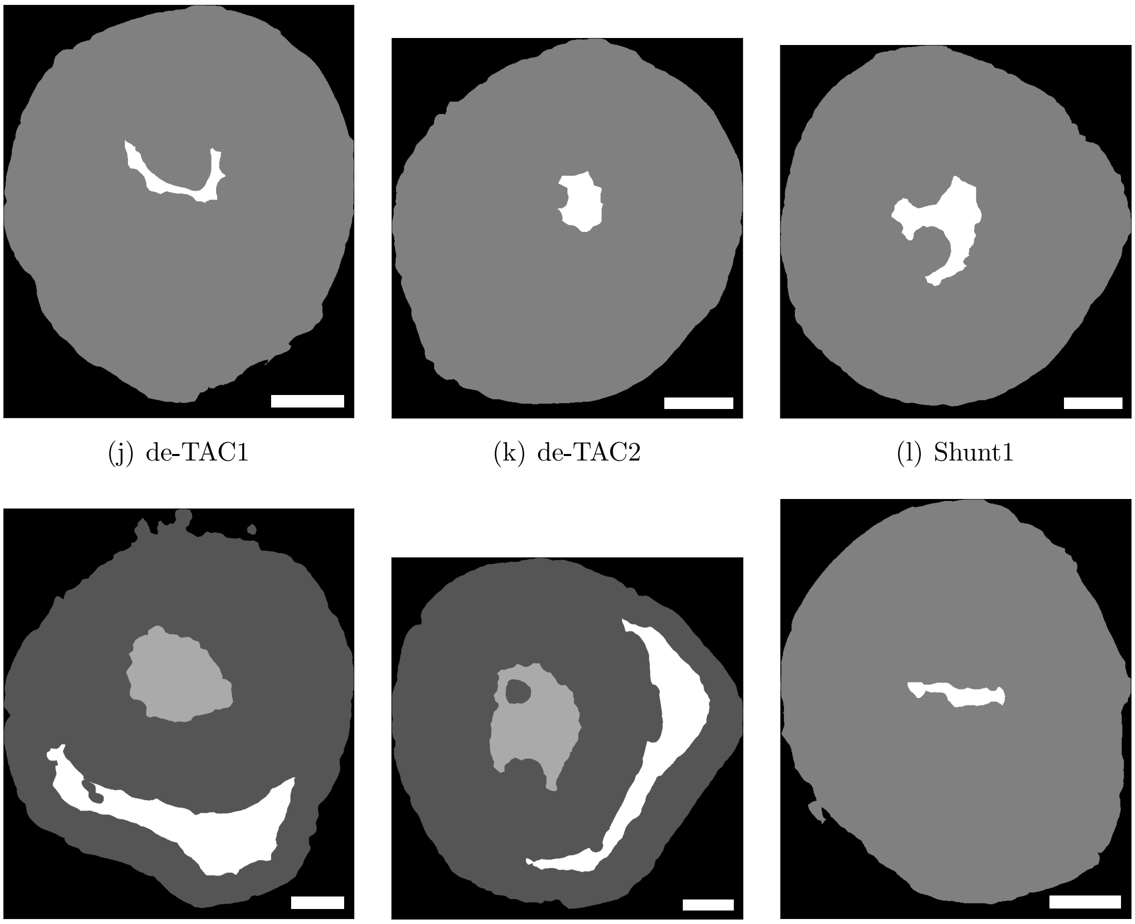

(j) de-TAC1 (k) de-TAC2 (l) Shunt1

(m) Shunt2 (n) Shunt3 (o) Unknown1

Figure S.10.: The results of every segmentation are shown with one slice each. The masks I_{mask} are sorted by their sample names. The individual pathology is given in table S.1.

Heart alignment

Before, the 18-segment models are created, the orientation of all hearts has to be aligned. The determination of the inertia tensor and the wall thickness calculation worked out by Jan Meyer in the framework of a laboratory practical.

S.1.4. Preparation of the 18 segment model

The desired Valentine orientation is achieved in 2 steps. First, by using the inertia tensor of the heart tissue and the ventricles to get the SA orientation and second, by rotating the apex p_a of the left ventricle under the centre of mass p_{com} to get the HLA & VLA orientation.

SA orientation The inertia tensor of each segmentation is calculated and diagonalised. The inertia tensor is determined from a reduced heart mask. The mask is cut-off in z-direction to omit segmentation fragments of other tissue and blood vessels. The cut-off height is given in table S.3. From the eigenvalues, the three eigenvectors are determined which span a new inertia system with the correct orientation of the SA axis. Afterwards, the hearts are rotated into the new inertia system with the Euler angles $\epsilon_i, i \in \{z, y, x\}$, derived from the eigenvectors. The determined angles are presented in table S.3. The hearts are rotated around the z-axis first, then rotated around the y-axis and lastly around the x-axis. The SA orientation is visualised in figure S.11.

HLA & VLA orientation After the SA orientation, the HLA & VLA orientation needs to be corrected. This is done by determining the tip of the apex p_a and the centre of mass p_{com} of the ventricle. The tip of the apex is calculated by determining the farthest point in the left ventricle from p_{com} towards the apex. With these points, one can calculate two angles $\gamma_i, i \in \{y, x\}$ to rotate the ventricle's apex p_a under the centre of mass p_{com}, so that the two points are aligned in the x-y-cross section. The angles are calculated with

$$
\begin{aligned}
\gamma_y &= \arctan \frac{p_{com,x} - p_{a,x}}{p_{com,z} - p_{a,z}} \\
\gamma_x &= \arctan \frac{p_{com,y} - p_{a,y}}{\tilde{r}} \\
\tilde{r} &= \sqrt{\left(p_{com,x} - p_{a,x}\right)^2 + \left(p_{com,z} - p_{a,z}\right)^2}.
\end{aligned} \tag{S.1}
$$

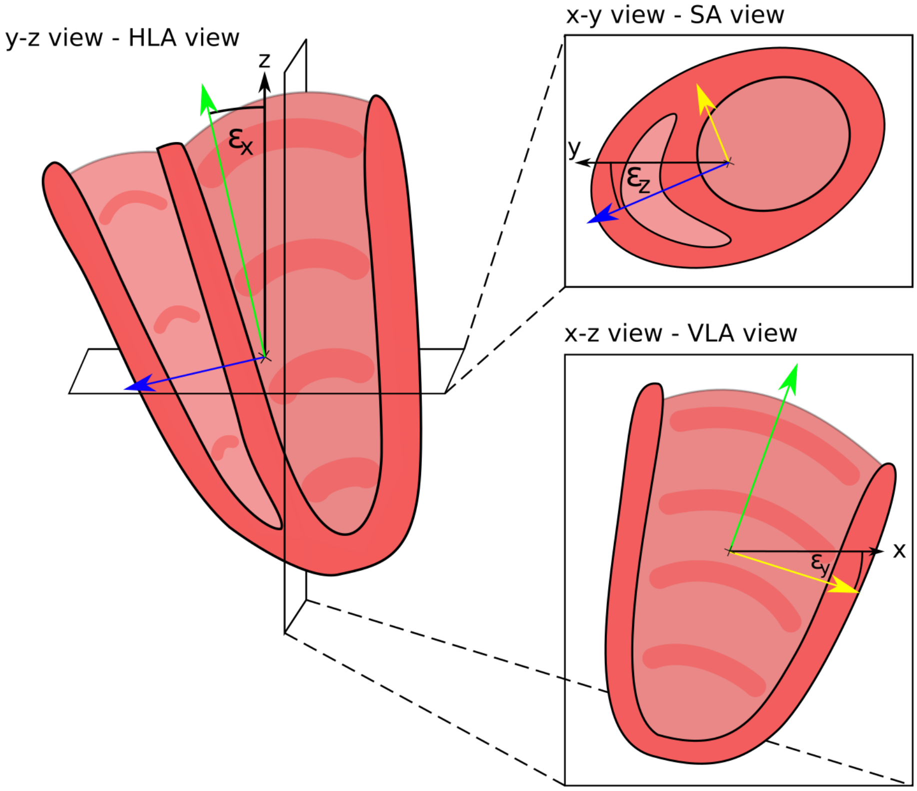

Figure S.11.: The heart with the eigenvectors of the inertia tensor (green, blue, yellow arrows) and the Euler angles ϵ_z, ϵ_y and ϵ_x are shown in the different heart views. The eigenvector of the smallest eigenvalue is depicted in green. The eigenvector of the second smallest eigenvalue is depicted in blue and the eigenvector of the largest eigenvalue is shown in yellow. The important eigenvector of the SA orientation is the eigenvector with the second smallest eigenvalue (blue).

Table S.3.: Overview of the Euler angles and the cut-off heights that were used for each sample in the course of the SA orientation.

sample	Euler Angles			cut-off height
	ϵ_z [°]	ϵ_y [°]	ϵ_x [°]	[voxel]
DMD1	9.5	10.73	-24.96	1363
TAC1	177.89	8.19	-4.13	1152
Control1	2.81	2.71	0.46	958
MI1	-0.18	-1.93	0.13	1061
MI2	178.17	6.21	9.82	954
Control2	165.38	-25.8	29.6	1181
TAC2	9.7	14.24	177.28	1333
TAC3	5.15	22.51	-11.24	1326
TAC4	-0.09	-8.0	3.55	1176
de-TAC1	-0.92	-0.8	179.79	845
de-TAC2	0.0	0.04	-0.03	1076
Shunt1	-16.2	6.59	3.55	1247
Shunt2	8.68	8.08	-179.93	1257
Shunt3	167.42	72.33	120.27	1491
Unknown1	179.97	-3.48	4.83	835

Again, there is a cut-off height to omit the fragments from the calculation of the centre of mass p_{com}. The centre of mass p_{com}, the apex's tip p_α and the resulting angles γ_y and γ_x are shown in figure S.12. The resulting angles and the cut-of height are shown in table S.4.

18 segment models The correctly oriented masks are cut-off to exclude unnecessary heart tissue. The cut-off height is given in table S.5. Then, the 18 segment model is created on the mask to label the different areas. On the basis of the oriented mask, the heart section volumes and the wall thickness are determined and the results are averaged over the individual segments. To calculate the averages of the shape measures c_l, c_p and c_s as well as the nematic order parameters S and η, the correctly oriented 18 segment model is rotated back into the initial position and the back rotated mask is multiplied with the data.

Statistical analysis

In the following, the 18 segment models for the wall thickness, the heart volumes, the shape measures and the nematic order parameters are presented as bullseye plots

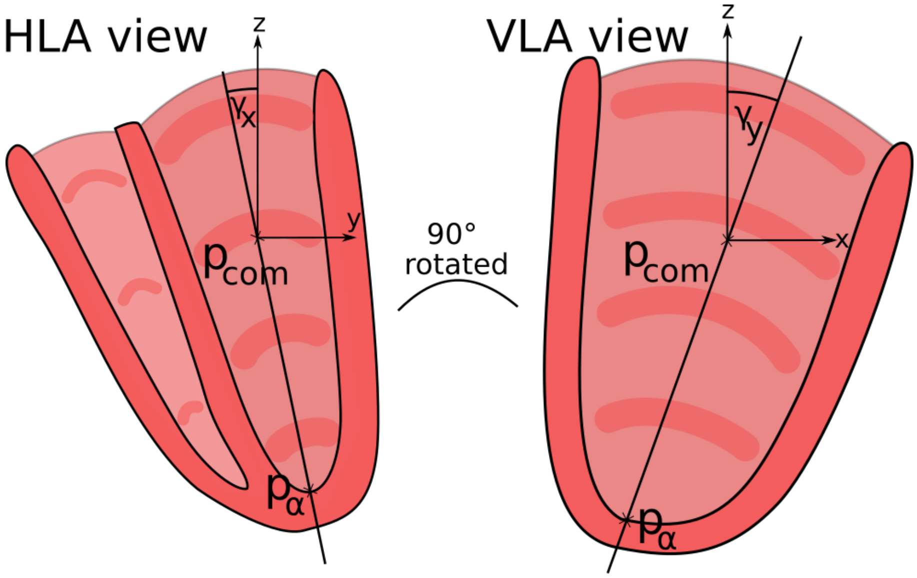

Figure S.12.: The HLA and VLA views of the heart are shown with the centre of mass p_{com} and the apex's tip p_{α}. A line is drawn through the two points and the rotation angles are determined in relation to the z-axis.

Table S.4.: Overview of the angles and the cut-off heights that were used for each sample in the course of the VLA & HLA orientation.

sample	angle γ_y [°]	angle γ_x [°]	cut-off height [voxel]
DMD1	59.88	27.07	1363
TAC1	17.53	18.66	1207
Control1	13.0	11.24	861
MI1	18.64	14.1	1061
MI2	63.99	19.8	1216
Control2	55.38	-24.97	1209
TAC2	34.0	0.14	1602
TAC3	-35.19	8.32	1326
TAC4	20.34	-16.6	1357
de-TAC1	-5.69	-8.74	1018
de-TAC2	-1.83	-2.35	1140
Shunt1	-14.88	-19.08	1270
Shunt2	18.32	-2.18	1539
Shunt3	-9.02	-10.21	1499
Unknown1	16.56	7.64	1210

Table S.5.: Overview of the cut-off heights that were used for each sample for the creation of the 18 segment models.

sample	cut-off height [voxel]
DMD1	665
TAC1	749
Control1	894
MI1	739
MI2	506
Control2	767
TAC2	967
TAC3	818
TAC4	808
de-TAC1	769
de-TAC2	832
Shunt1	902
Shunt2	1087
Shunt3	1172
Unknown1	888

for each heart. They are grouped by their pathologies. The 18-segment models of pathogenic hearts are compared to the two control hearts. As there are only few samples per pathology, descriptions of differences are limited to tendencies and no statement concerning their statistical significance can be made. The tendencies of differences will be described and underlined by average segment values outside the 1-σ-intervals of the control hearts. The heart with unknown pathology is kept and regarded concerning the different heart parameters to find similarities to the known pathologies.

S.1.5. Wall thickness

The mean wall thickness values are displayed in the bullseye plots. For each sample, one bullseye plot is shown and they are grouped with regard to the different pathologies. The wall thickness is displayed in mm. The colour map ranges from the minimum wall thickness to the maximum wall thickness over all samples. The wall thickness was calculated on $8 \times 8 \times 8$-binned heart masks. Consequently, in order to omit the effect of the binning, the values received for the wall thickness were multiplied by 8. Tests with different binning sizes showed that the binning size has only a minor effect on the precision of the wall thickness. The calculation of the

wall thickness is shown in figure S.13.

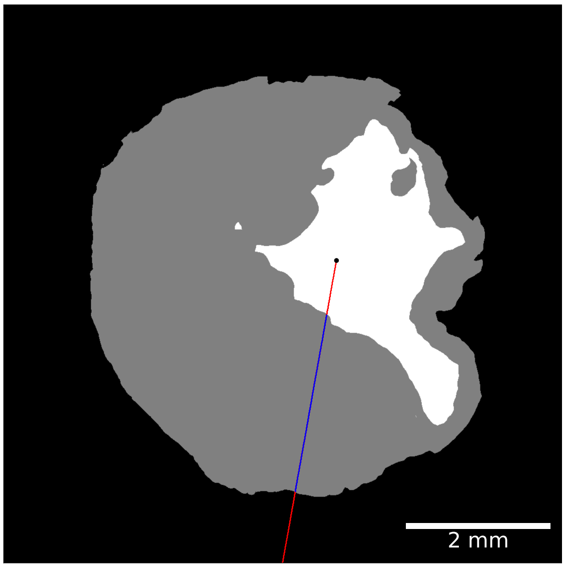

Figure S.13.: The wall thickness is measured by drawing a line (red) from the centre of the left ventricle and the line is multiplied with the heart tissue to determine the line crossing the heart tissue. The crossing line is shown in blue. Then, the line is measured from the start point to the end point. The here shown wall thickness r is 2.47 mm.

Control hearts The two control hearts show an evenly spread wall thickness across all angles and the heart wall thickness decreases from the apical volume towards the apex. The sample Control1 has an apical wall thickness between 1.47 and 1.99. The mid wall thickness ranges from 2.21 to 2.66 and the basal wall thickness ranges from 2.11 to 2.43. The maximal wall thickness is located in the mid antero- and inferolateral segments. The apical volume has a decreased wall thickness with the minimum located in the apical anterior segment. The sample Control2 is not as uniformly shaped along all angles. The sample has an apical wall thickness between 1.44 and 1.89. The mid wall thickness ranges from 1.81 to 2.56 and the basal wall ranges from 2.22 to 2.68.

MI hearts The 18 segment models of the MI hearts are shown in figure S.15. The sample MI1 has a reduced wall thickness at the mid and apical anteroseptal and inferoseptal segments as well as a reduced wall thickness in the anterior and antero-lateral segments. The average wall thickness of the mid anterior, anteroseptal and the anterolateral segments and the respective segments' values of the control hearts

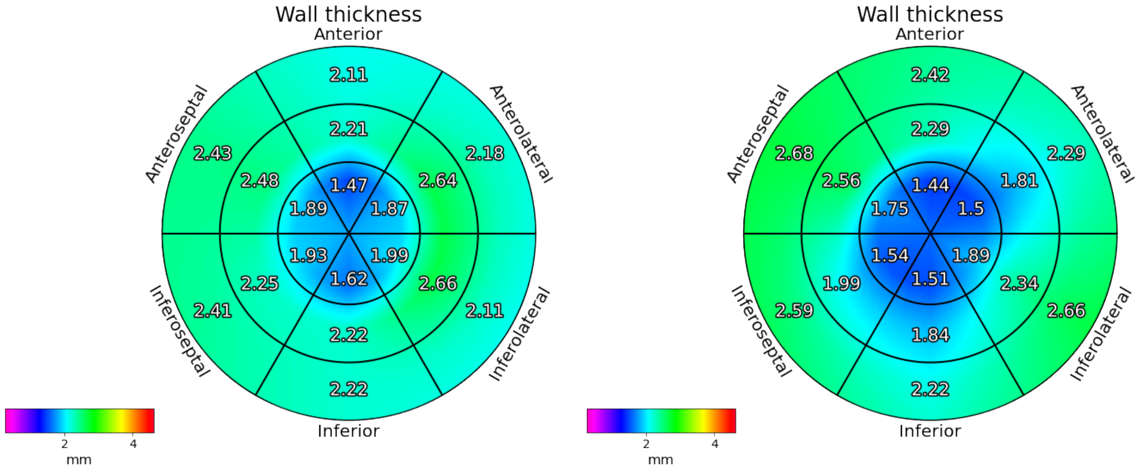

(a) Control1 - wall thickness

(b) Control2 - wall thickness

Figure S.14.: The bullseye plots of the two control hearts' cardiac wall thickness are shown. The colour map ranges from the minimum wall thickness to the maximum wall thickness over all samples.

are outside each's 1-σ-intervals. An increased wall thickness is present in the anteroseptal segments of sample MI2 and it stretches into the inferoseptal and anterior segments. Also a decrease in wall thickness in the antero- and inferolateral volume as well as in the inferior segments is seen, especial in apical segment, which stretches into the inferoseptal segment. The average wall thickness of the inferolateral and anterolateral segments as well as the apical inferior segment and the corresponding values of the control hearts are outside of their respective 1-σ-intervals.
The strongly reduced wall thickness might be used to locate the infarct.

TAC The 18 segment models of the TAC hearts are shown in figure S.16. In figure S.16(a) with sample TAC1, the wall thickness is increased, especially in the anterolateral and inferolateral sections, compared to the control hearts. The values of inferolateral and the anterolateral segments are outside the 1-σ-intervals of the respective segments of the control hearts. The sample TAC2 appears to have an overall increased wall thickness, which is shown in figure S.16(b), especially the basal anterolateral segment's value is outside the 1-σ-intervals of the control hearts. A general wall thickness increase can be seen in sample TAC3, shown in figure S.16(c), except for the mid anterior segment. The basal anterolateral segment's wall thickness is outside the 1-σ-interval of the control hearts. The sample TAC4 has a reduced wall thickness in the anterior volumes. Otherwise, the wall thickness is

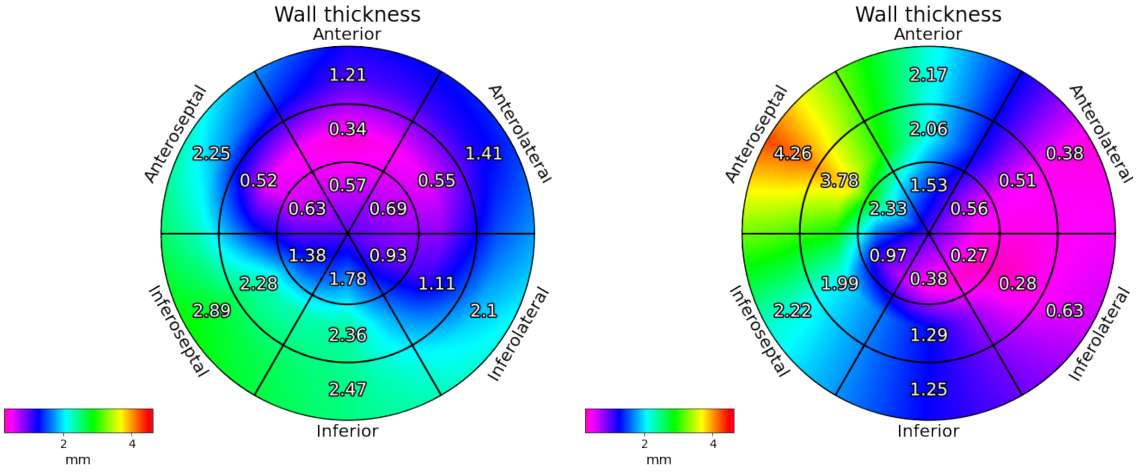

(a) MI1 - wall thickness

(b) MI2 - wall thickness

Figure S.15.: The bullseye plots of the two MI hearts' cardiac wall thickness are shown.

increased, especially in the apical volume, compared to the control hearts.

Shunt The 18 segment models of the shunt hearts are shown in figure S.17. An increased wall thickness occurs in the apical and mid sections in the inferoseptal, inferior, inferolateral and anterolateral segments for the sample Shunt1 compared to the control hearts. The apical inferior segment's average wall thickness lies outside the 1-σ-interval of the control hearts. The sample Shunt2 has an equal to slightly reduced wall thickness compared to the control hearts and the sample Shunt3 has a wall thickness comparable to the control hearts as shown in figure S.17(c).

de-TAC The 18 segment models of the de-TAC hearts are shown in figure S.18. Shown in figure S.18(a), the sample de-TAC1 shows a slight increase in wall thickness in the infero- and anteroseptal segments compared to the control hearts. A comparably decreased wall thickness is present in the antero- and inferoseptal segments as well as in the inferolateral segments of sample de-TAC2, shown in figure S.18(b).

DMD The 18 segment models of the DMD hearts are shown in figure S.19(a). The sample DMD1 shows a slight decrease in wall thickness in the mid and apical segments and high increase in wall thickness in the anterolateral segments compared to the control hearts. The average values of the anterolateral segments lie outside

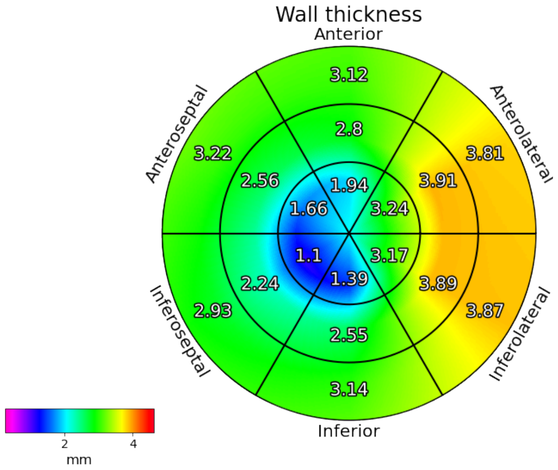

(a) TAC1 - wall thickness

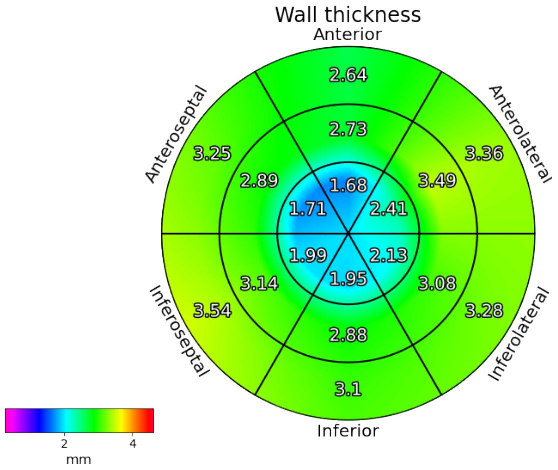

(b) TAC2 - wall thickness

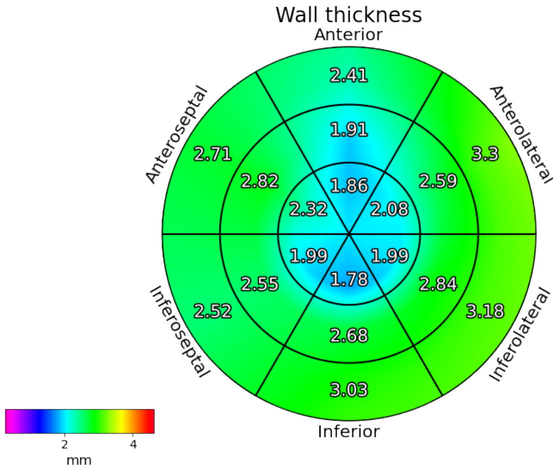

(c) TAC3 - wall thickness

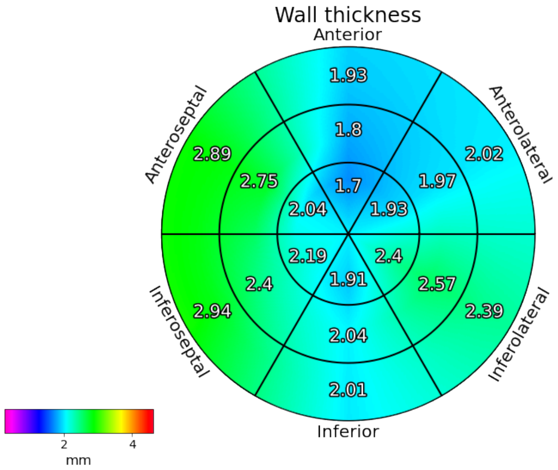

(d) TAC4 - wall thickness

Figure S.16.: The bullseye plots of the four TAC hearts' cardiac wall thickness are shown.

the respective 1-σ-intervals of the control hearts. The increase in wall thickness is also present in the inferoseptal and -lateral segments.

Unknown The wall thickness of sample Unknown1 in the mid inferoseptal segment is increased and the average value lies outside the 1-σ-interval of the compared control hearts. The bullseye plot is shown in figure S.19(b).

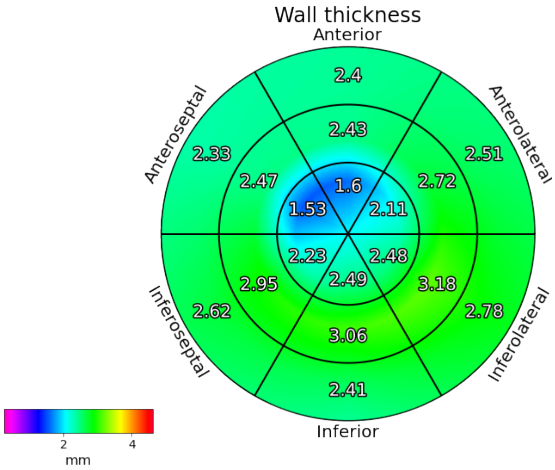

(a) Shunt1 - wall thickness

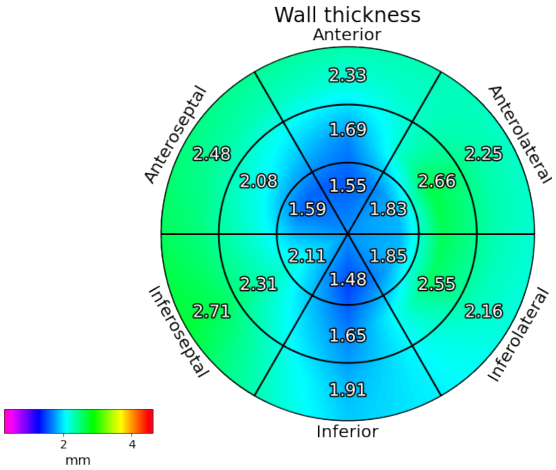

(b) Shunt2 - wall thickness

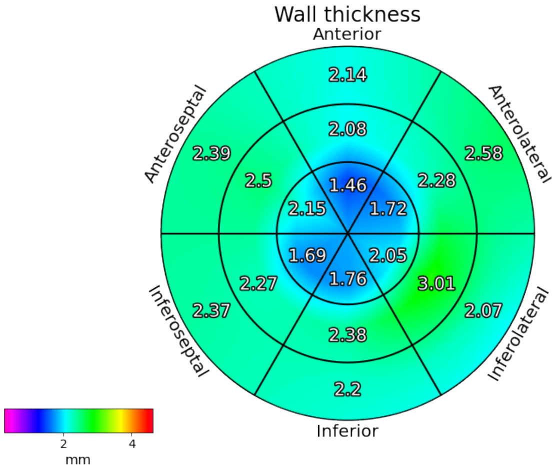

(c) Shunt3 - wall thickness

Figure S.17.: The bullseye plots of the three shunt hearts' cardiac wall thickness are shown.

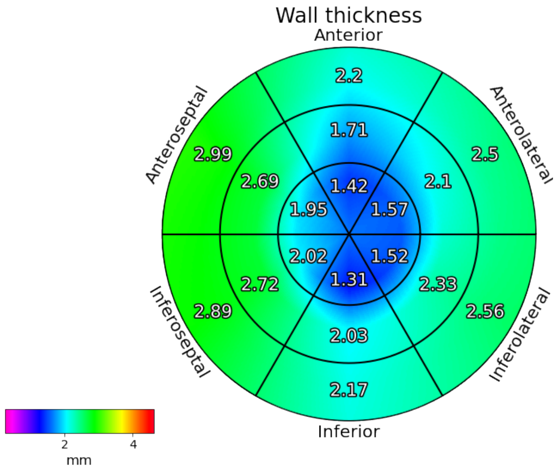

(a) de-TAC1 - wall thickness

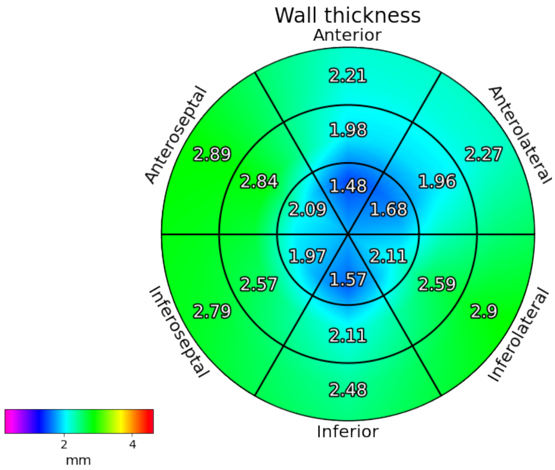

(b) de-TAC2 - wall thickness

Figure S.18.: The bullseye plots of the three de-TAC hearts' cardiac wall thickness are shown.

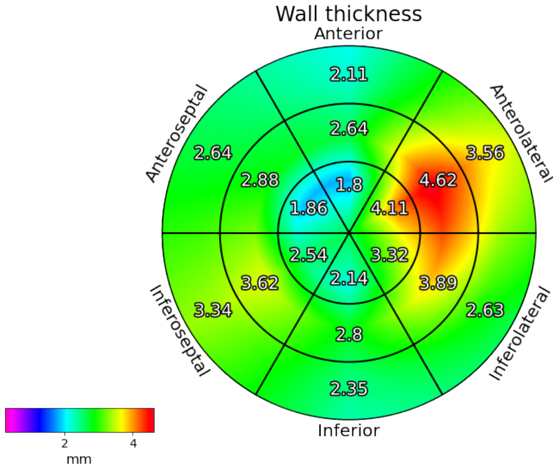

(a) DMD1 - wall thickness

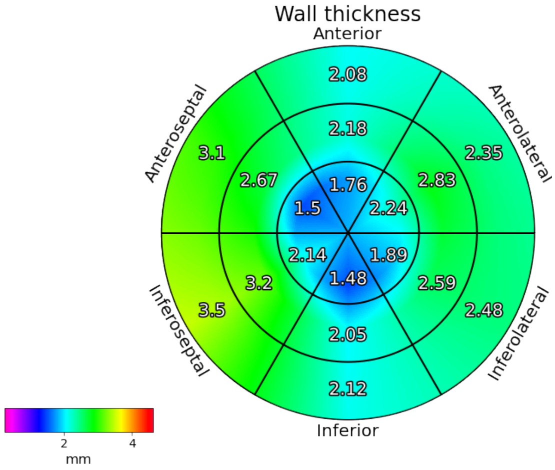

(b) Unknown1 - wall thickness

Figure S.19.: The bullseye plots of the DMD heart's cardiac wall thickness and the unknown heart model's wall thickness are shown.

S.1.6. Volumes

The segments' volumes are displayed in the bullseye plots. For each sample, one bullseye plot is shown and they are grouped according to the different pathologies. The segments' volumes are displayed in mm^3.

Control hearts The control hearts' bullseye plots are shown in figure S.20. The control hearts have a smaller apical volume compared to the mid and basal heart volumes. The basal volumes are the largest heart volumes, but the mid volume are in close vicinity. The two control hearts' basal volumes range from 4.24 to 6.81, the mid volumes range from 3.14 to 5.56 and the apical volumes range from 1.38 to 2.64.

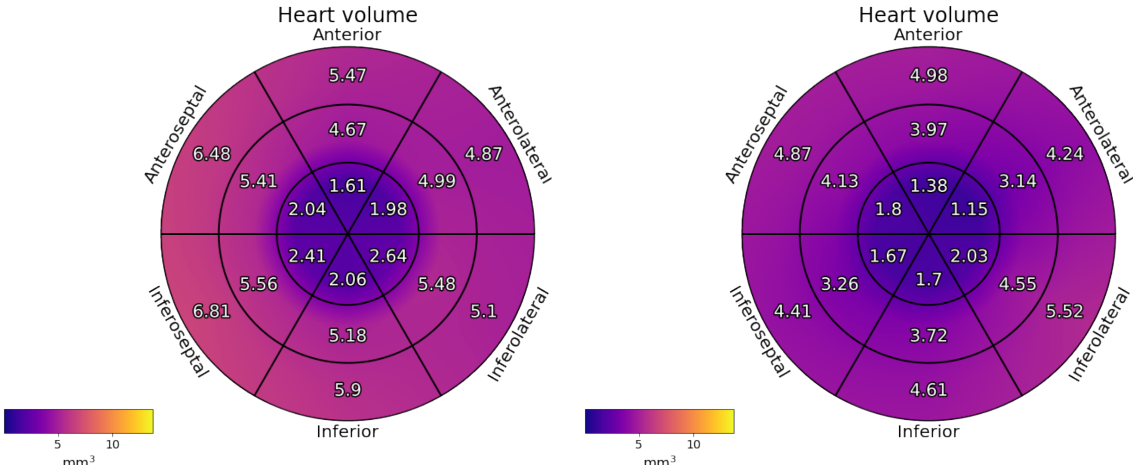

(a) Control1 - volume (b) Control2 - volume

Figure S.20.: The bullseye plots of the two control hearts' cardiac volumes of the different segments are shown.

MI The MI hearts' bullseye plots are shown in figure S.21. The sample MI1 has a distinct reduced heart volume in the anterior and anterolateral volumes, but an increased heart volume in the inferoseptal and inferior segments compared to the control hearts. The sample MI2 has an increased heart volume in the anteroseptal segments and a distinct reduction of the heart volume in the inferior, inferolateral and anterolateral segments is seen.

TAC The TAC hearts' bullseye plots are shown in figure S.22. The sample TAC1 shows a distinct increase of the anterolateral and inferolateral heart volumes, whereas

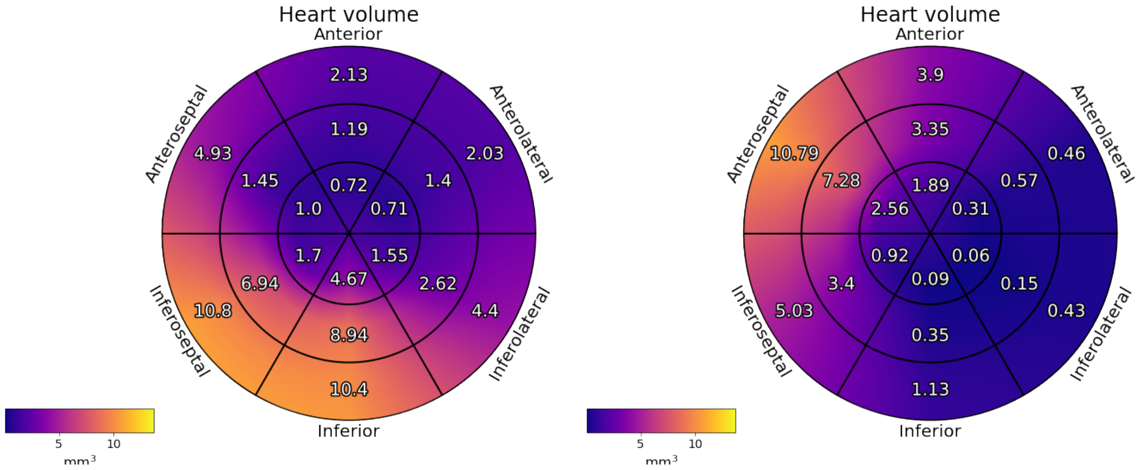

(a) MI1 - volume (b) MI2 - volume

Figure S.21.: The bullseye plots of the two MI hearts' cardiac volumes of the different segments are shown.

the anterior and inferior heart volumes are only slightly increased. A general increased heart volume is present in the sample TAC2. The samples TAC3 and TAC4 also have an increased heart volume.

shunt The shunt hearts' bullseye plots are shown in figure S.23. The sample Shunt1 has an overall increased heart volume and the other samples Shunt2 and Shunt3 have a distinctly increased heart volume.

de-TAC The control hearts' bullseye plots are shown in figure S.24. The sample de-TAC1 has a similar heart volume compared to the control hearts. An increased heart volume is seen in the antero- and inferoseptal, inferior and inferolateral segments of the sample de-TAC2.

DMD The DMD hearts' bullseye plot is shown in figure S.25(a). The anterolateral and inferoseptal volumes are enlarged in sample DMD1 and the inferolateral volume is slightly increased as well.

Unknown The DMD hearts' bullseye plot is shown in figure S.25(b). Similar to the wall thickness, the volume in the mid inferoseptal segment of the sample Unknown1 is increased compared to the control hearts.

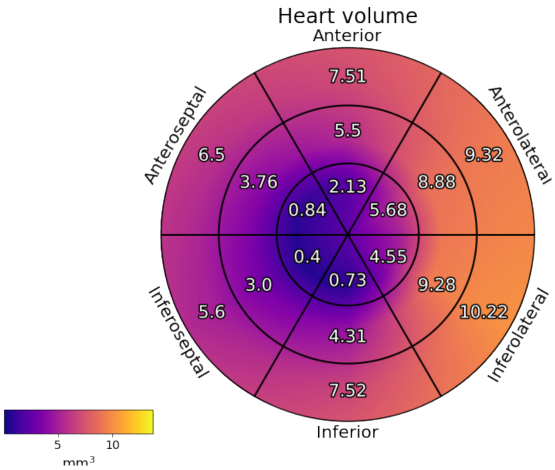

(a) TAC1 - volume

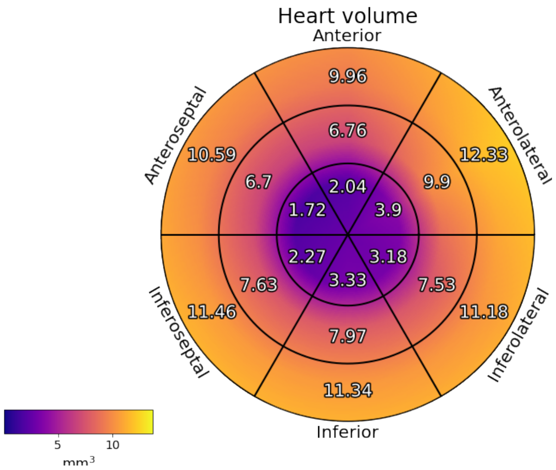

(b) TAC2 - volume

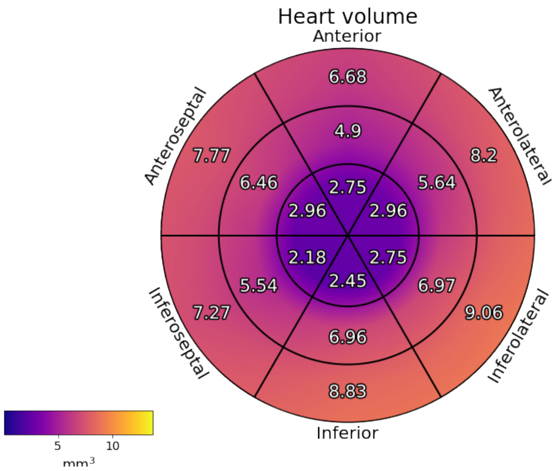

(c) TAC3 - volume

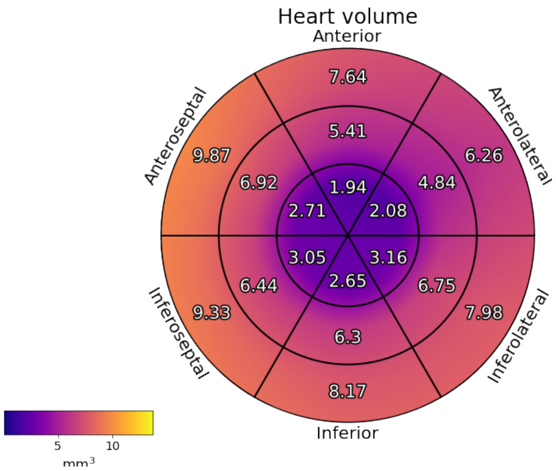

(d) TAC4 - volume

Figure S.22.: The bullseye plots of the four TAC hearts' cardiac volumes of the different segments are shown.

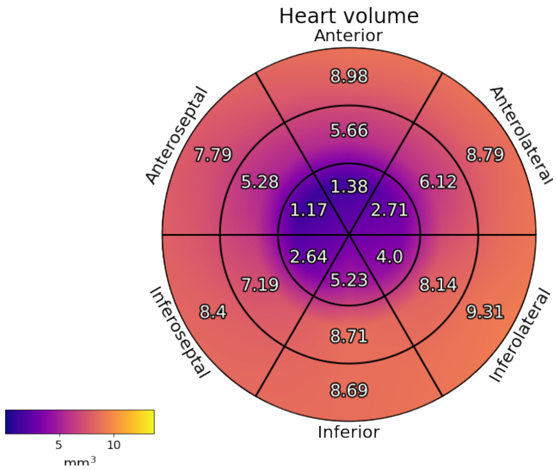

(a) Shunt1 - volume

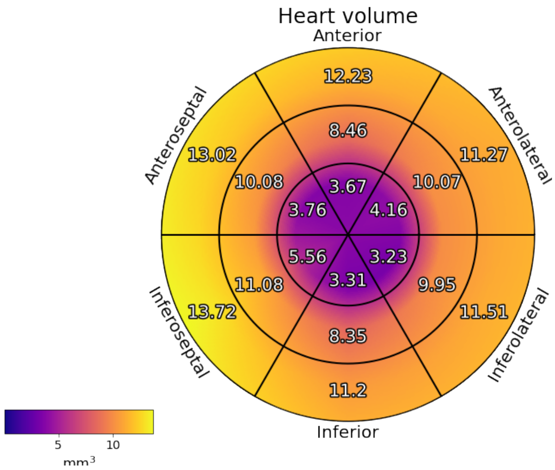

(b) Shunt2 - volume

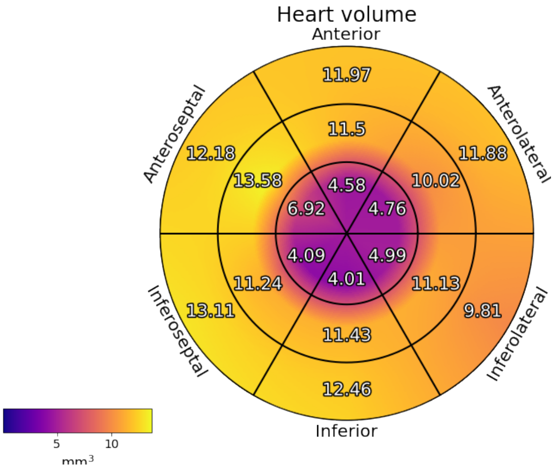

(c) Shunt3 - volume

Figure S.23.: The bullseye plots of the three shunt hearts' cardiac volumes of the different segments are shown.

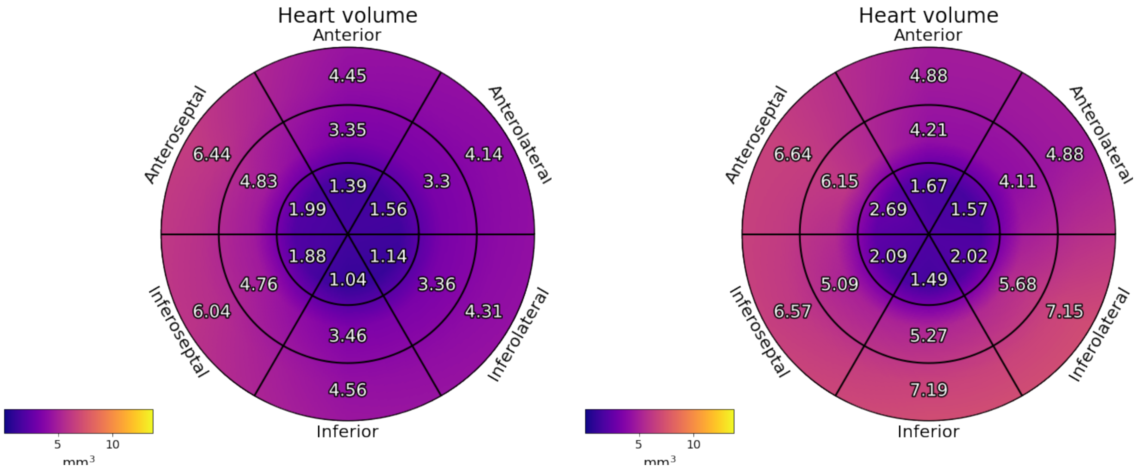

(a) de-TAC1 - volume

(b) de-TAC2 - volume

Figure S.24.: The bullseye plots of the two de-TAC hearts' cardiac volumes of the different segments are shown.

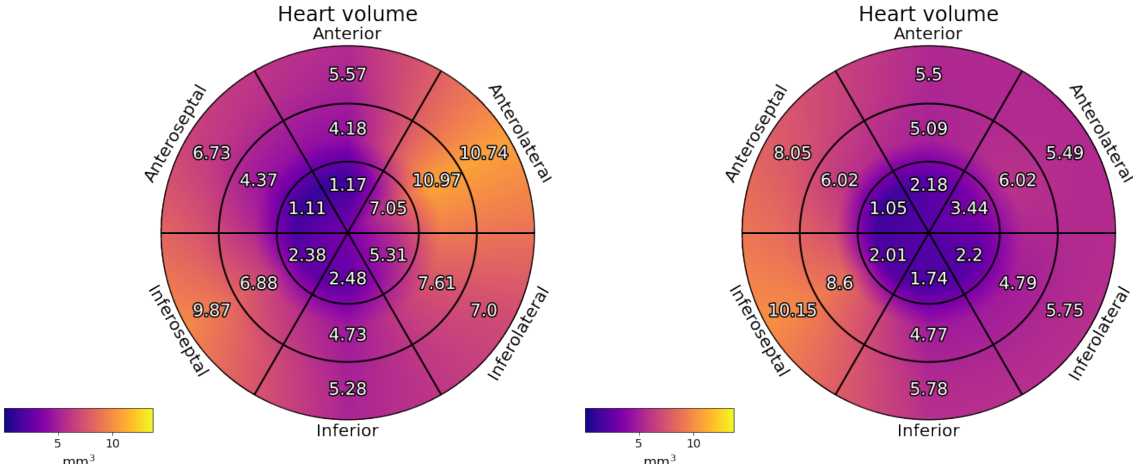

(a) DMD1 - volume

(b) Unknown1 - volume

Figure S.25.: The bullseye plots of the DMD heart's cardiac volumes and the unknown heart model's volumes are shown.

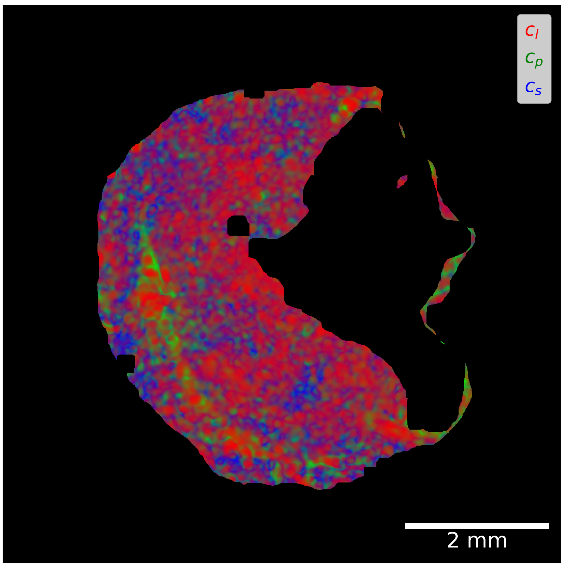

Figure S.26.: A slice of the MI2 heart's shape measures c_l (red), c_p (green) and c_s (blue) are displayed.

S.1.7. Shape measures

The mean shape measures c_l, c_p and c_s are displayed in the bullseye plots. For each sample, one bullseye plot is shown and they are grouped with respect to the different pathologies.
The structure tensor were calculated with the standard derivations $\sigma = 1$ voxel, to reduce the local noise, and $\rho = 4$ voxels, to average over the ROI of the shape measures. The shape measures are shown for one single slice in figure S.26.

Control hearts The bullseye plots of the control hearts' shape measures c_l, c_p and c_s are shown in figure S.27. The control hearts have similar shape measures. The shape measure c_l is equally distributed over all segments, with c_l varying between $0.4 - 0.5$. Also, the shape measure c_p is equally distributed with values ranging from $0.25 - 0.45$. In the case of the sample Control1, the values of the shape measure c_s are also equally distributed among the segments, but, in the case of sample Control2, the c_s values of the mid and basal segments are higher than the values of the apical segments and higher than the values of the segments of sample Control1.

MI The bullseye plots of the MI hearts' shape measure c_l are shown in figures S.28(a) and S.28(b). The shape measure c_l has reduced values in the anterior as well as the anteroseptal and -lateral segments of sample MI1. The apical inferior

c_l value lies below the 1-σ-interval of the control hearts. Furthermore, the apical inferoseptal and the apical and mid inferolateral c_l values are reduced compared to the control hearts. The sample MI2 has increased c_l values in the apical anterolateral segment compared to the control hearts.
The bullseye plots of the MI hearts' shape measure c_l are shown in figures S.28(c) and S.28(d). The shape measure c_p in the sample MI1 does not appear to be different from the c_p values of the control hearts. The sample MI2 has reduced c_p values in the apical inferolateral segment, lying outside the 1-σ-interval of the control hearts. Further there are reduced values in the basal infero- and anterolateral segment.
The bullseye plots of the MI hearts' shape measure c_l are shown in figures S.28(e) and S.28(f). In general, the shape measure c_s values are increased for the sample MI1 compared to the control hearts. This trend is especially seen for the mid and apical segments. The values of the mid and apical anterior and anterolateral as well as the apical inferoseptal and the apical inferior segments are above the 1-σ-intervals of the control hearts. For the sample MI2, the c_s values are increased in the inferior and inferolateral segments especially the apical inferolateral segment. The c_s values of the mid and apical inferior segments as well as the apical inferolateral segment are outside the 1-σ-interval of the control hearts.

TAC The bullseye plots of the TAC hearts' shape measures c_l, c_p and c_s are shown in figures S.29 and S.30. The shape measure c_l does not change in the samples TAC1 and TAC3 compared to the control hearts. The sample TAC2 shows increased c_l values compared to the control hearts and the sample TAC4 has increased c_l values in the apical segments.
The shape measure c_p shows no distinct change for the samples TAC1, TAC2, TAC3 compared to the control heart. The sample TAC4 has reduced c_p values in the apical segments.
The shape measure c_s shows reduced values in the apical segments compared to the control hearts. The sample TAC2 has reduced c_s values in the basal segments, while the sample TAC3 and TAC4 do not show any distinct variation from the control hearts.

Shunt The bullseye plots of the shunt hearts' shape measure c_l, c_p and c_s are shown in figures S.31 and S.32. The shape measures c_l, c_p and c_s do not show any differences for the samples Shunt1, Shunt2 and Shunt3 compared to the control

hearts.

de-TAC The bullseye plots of the de-TAC hearts' shape measure c_l, c_p and c_s are shown in figure S.33. The shape measures c_l, c_p and c_s do not show any differences for the samples de-TAC1 and de-TAC2 compared to the control hearts and the bullseye plots are displayed in figure S.33.

DMD The bullseye plots of the DMD heart's shape measure c_l, c_p and c_s are shown in figures S.34(a), S.34(c) and S.34(e). The shape measure c_l shows increased values in the anterior, anterolateral, inferolateral and inferior segments as well as in the apical anteroseptal segment.
Contrary to shape measure c_l, the shape measure c_p is reduced in the anterior, anterolateral, inferolateral and inferior volumes as well as in the apical anteroseptal segment compared to the control hearts. The apical inferior segment's value lies below the 1-σ-interval.
The sample DMD1 shows no distinct variation of the shape measure c_s values within the 18 segment model.

Unknown The bullseye plots of the unknown heart model's shape measure c_l, c_p and c_s are shown in figures S.34(b), S.34(d) and S.34(f). The shape measures c_l, c_p do not show any distinct change in the sample Unknown1 compared to the control hearts.
The sample Unknown1 has increased c_s values in the apical segments as well as in the mid inferior, inferoseptal and anteroseptal segments compared to the control hearts.

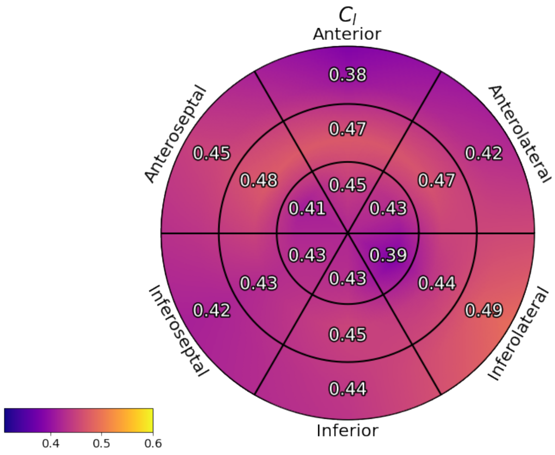

(a) Control1 - c_l

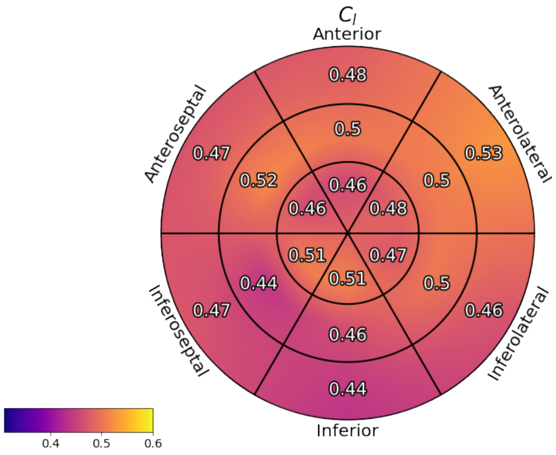

(b) Control2 - c_l

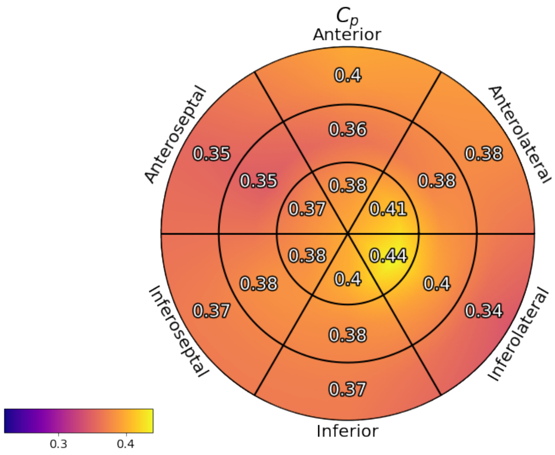

(c) Control1 - c_p

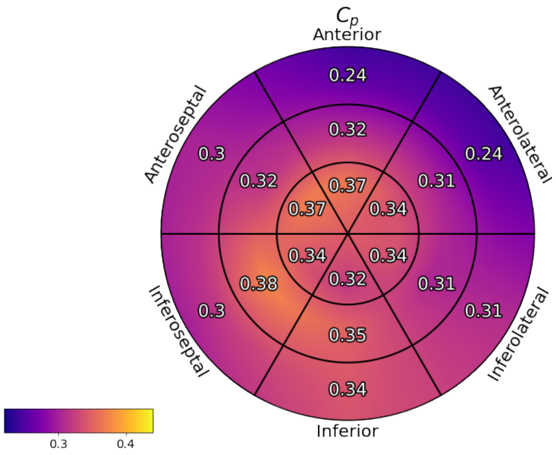

(d) Control2 - c_p

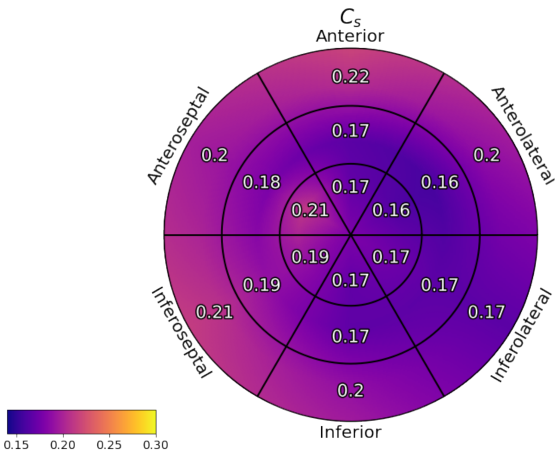

(e) Control1 - c_s

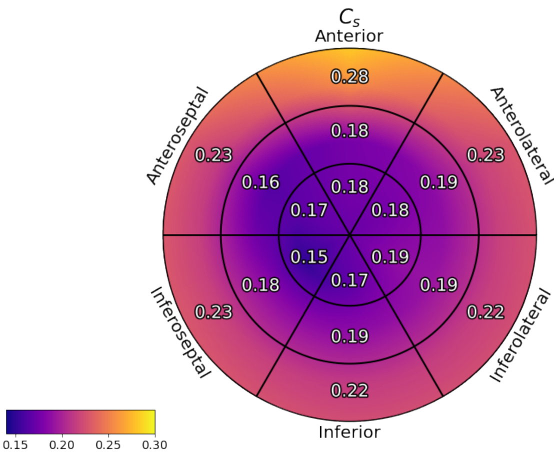

(f) Control2 - c_s

Figure S.27.: The bullseye plots of the two control hearts' shape measures c_l, c_p and c_s are shown.

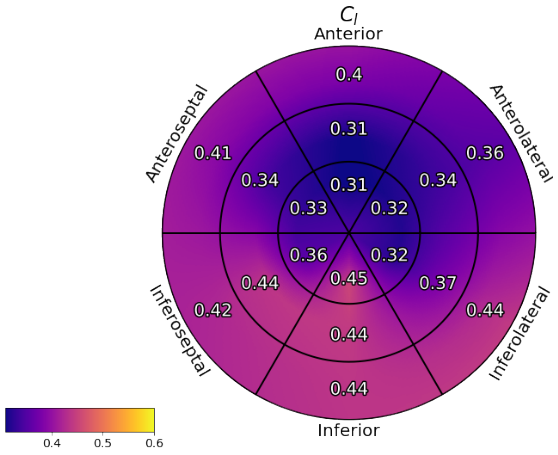

(a) MI1 - c_l

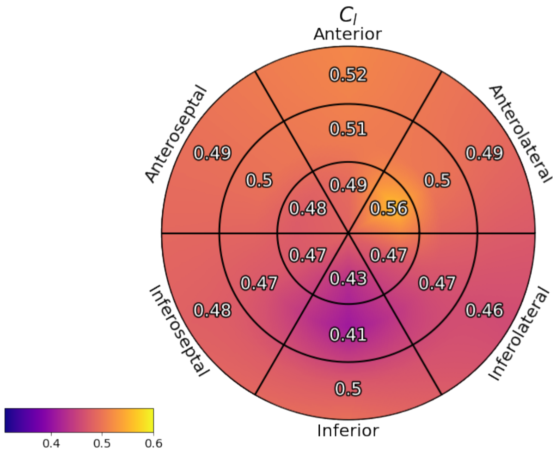

(b) MI2 - c_l

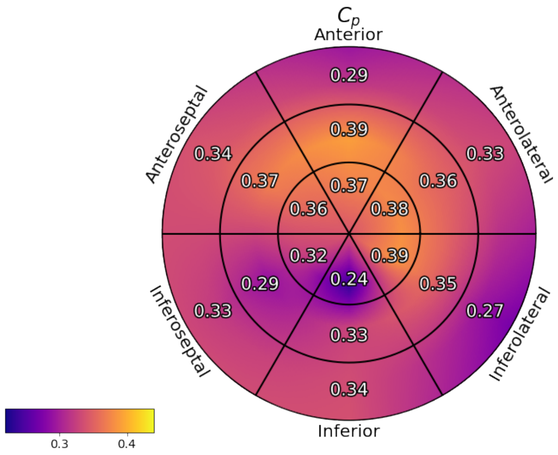

(c) MI1 - c_p

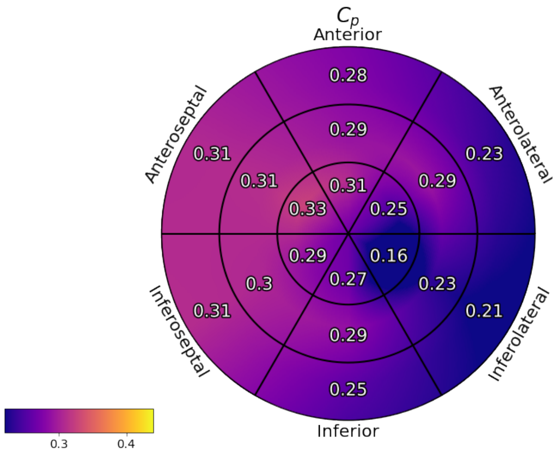

(d) MI2 - c_p

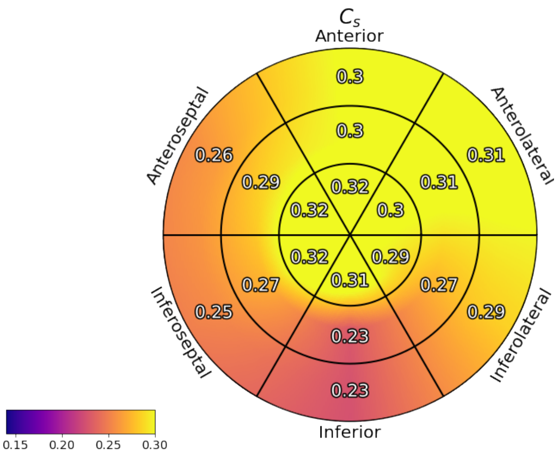

(e) MI1 - c_s

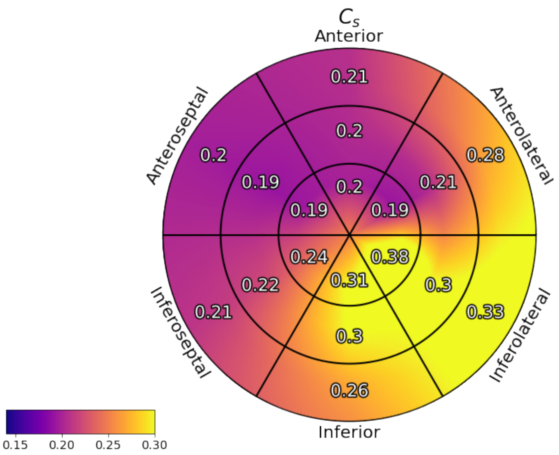

(f) MI2 - c_s

Figure S.28.: The bullseye plots of the two MI hearts' shape measures c_l, c_p and c_s are shown.

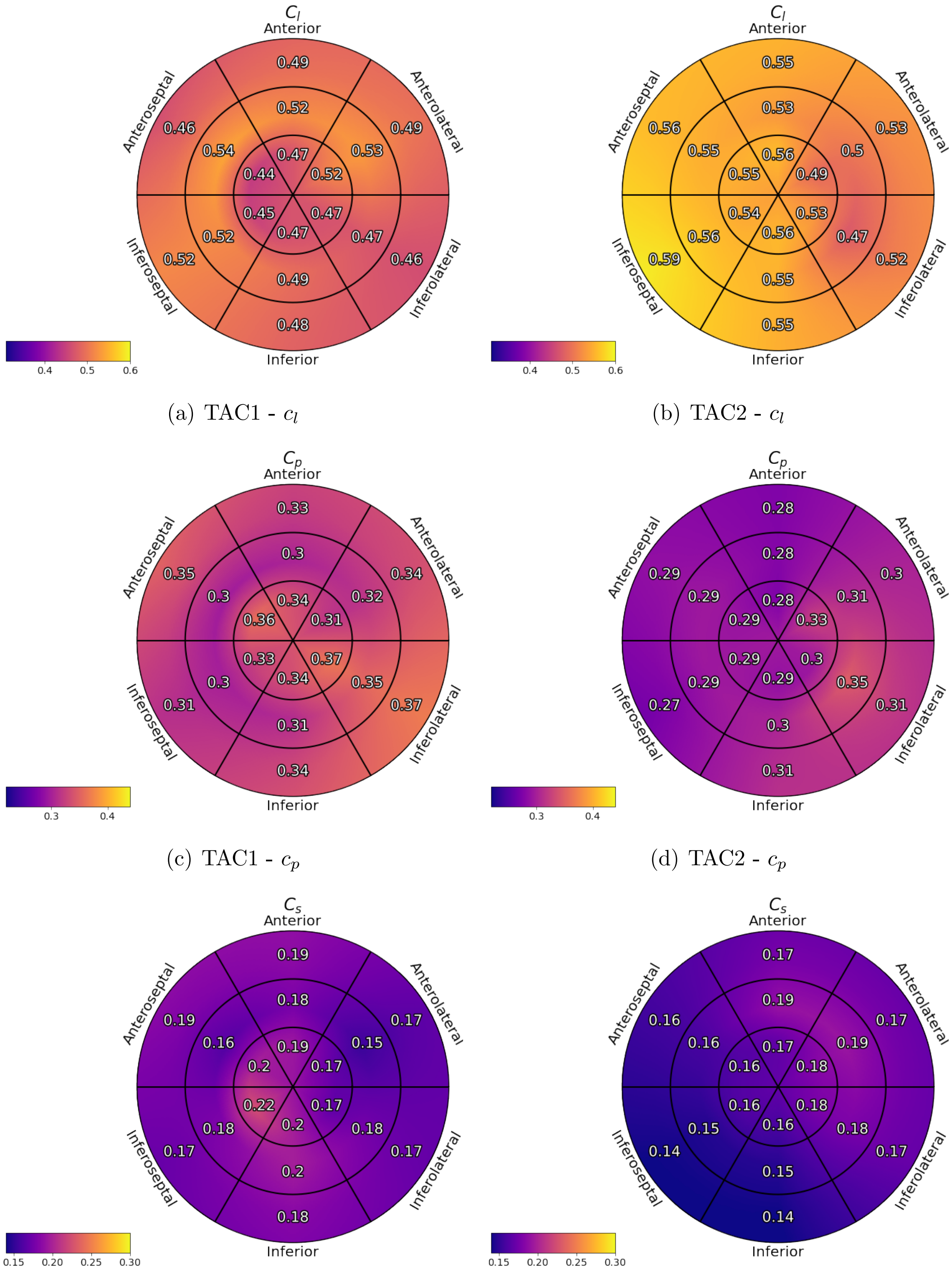

(a) TAC1 - c_l

(b) TAC2 - c_l

(c) TAC1 - c_p

(d) TAC2 - c_p

(e) TAC1 - c_s

(f) TAC2 - c_s

Figure S.29.: The bullseye plots of two TAC hearts' shape measures c_l, c_p and c_s are shown.

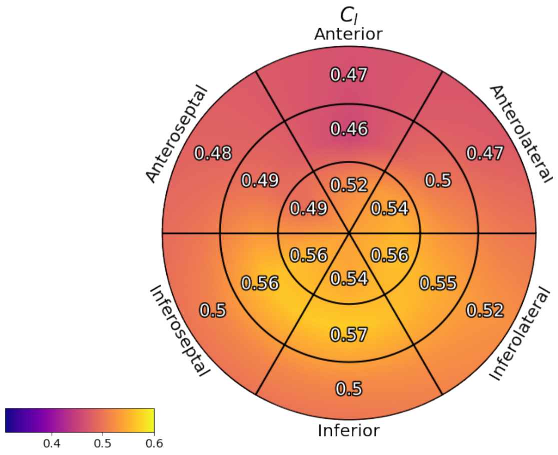

(a) TAC3 - c_l

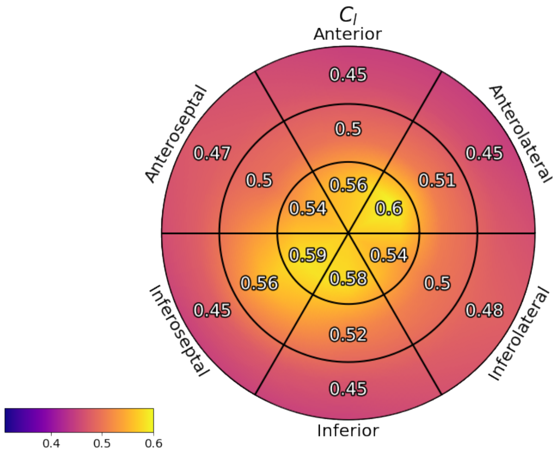

(b) TAC4 - c_l

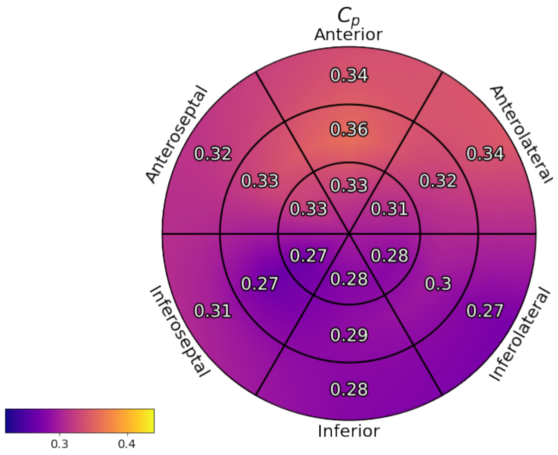

(c) TAC3 - c_p

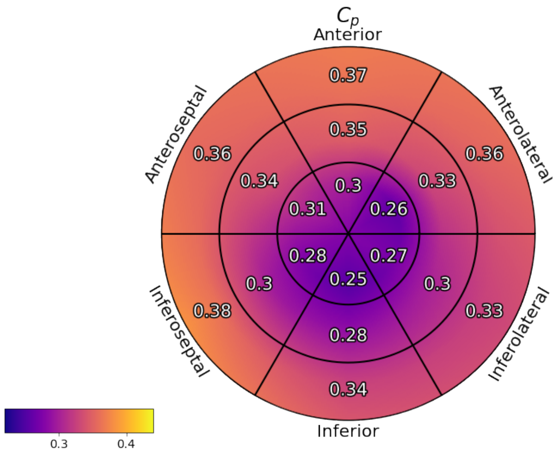

(d) TAC4 - c_p

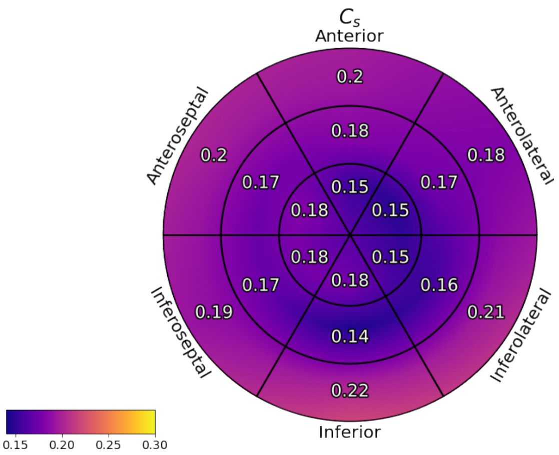

(e) TAC3 - c_s

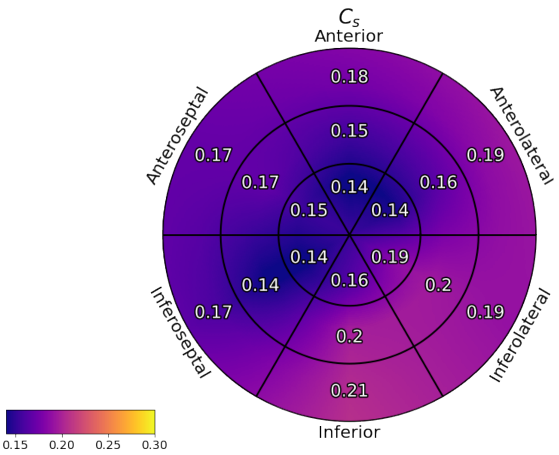

(f) TAC4 - c_s

Figure S.30.: The bullseye plots of the other two TAC hearts' shape measures c_l, c_p and c_s are shown.

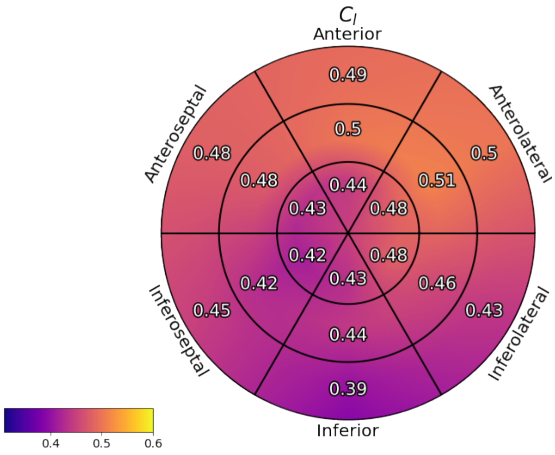

(a) Shunt1 - c_l

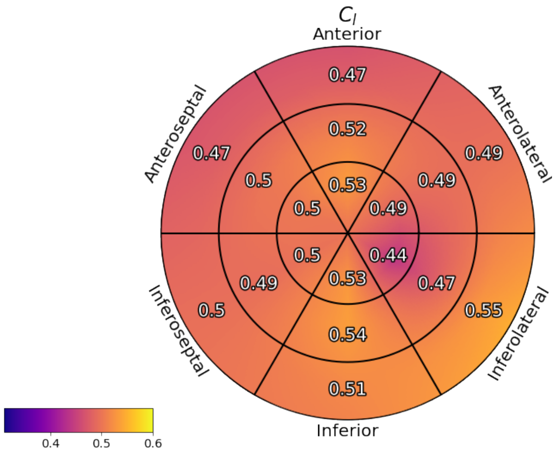

(b) Shunt2 - c_l

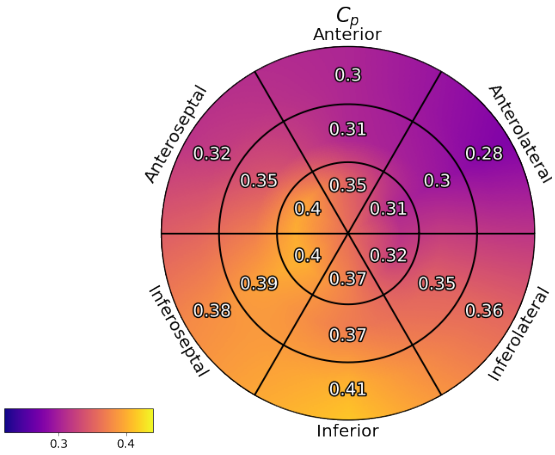

(c) Shunt1 - c_p

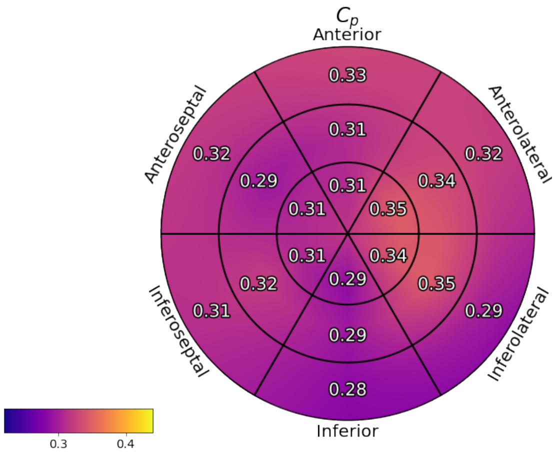

(d) Shunt2 - c_p

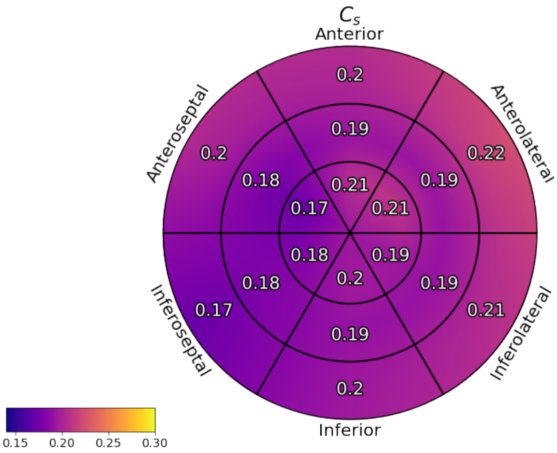

(e) Shunt1 - c_s

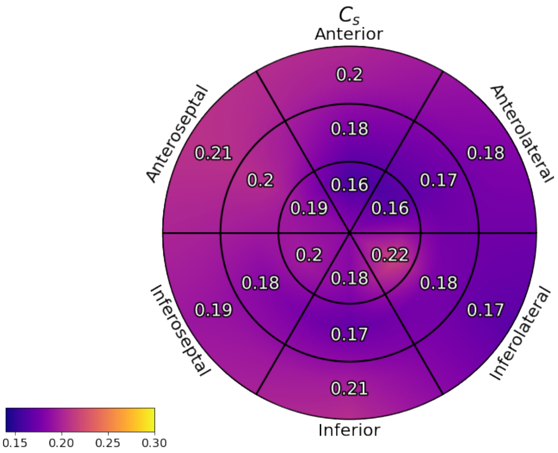

(f) Shunt2 - c_s

Figure S.31.: The bullseye plots of two shunt hearts' shape measures c_l, c_p and c_s are shown.

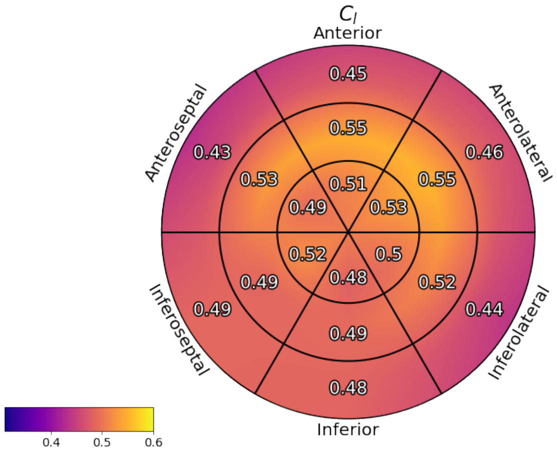

(a) Shunt3 - c_l

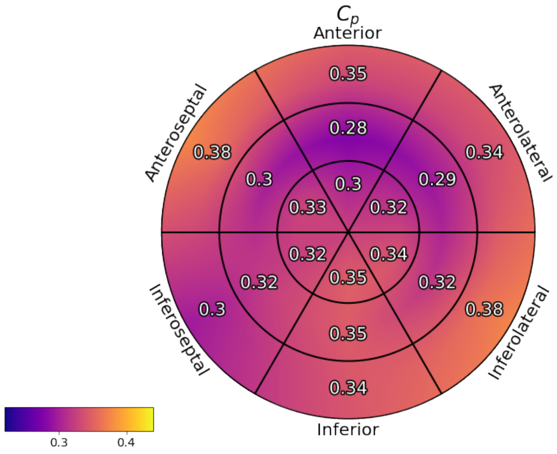

(b) Shunt3 - c_p

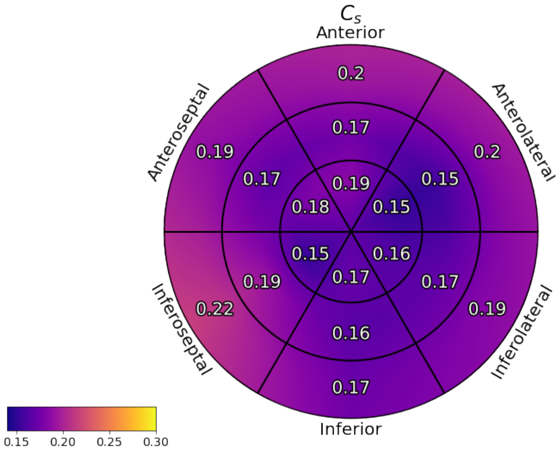

(c) Shunt3 - c_s

Figure S.32.: The bullseye plots of the last shunt heart's shape measures c_l, c_p and c_s are shown.

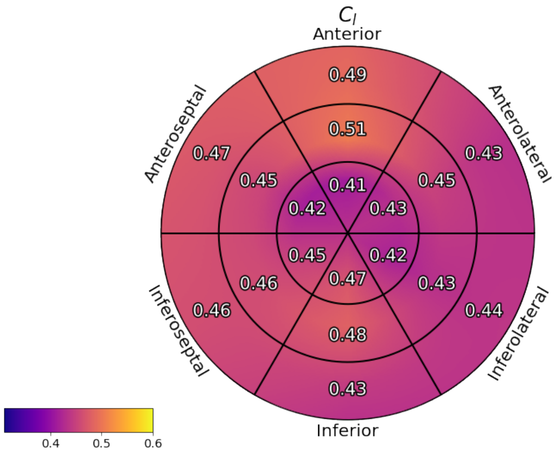

(a) de-TAC1 - c_l

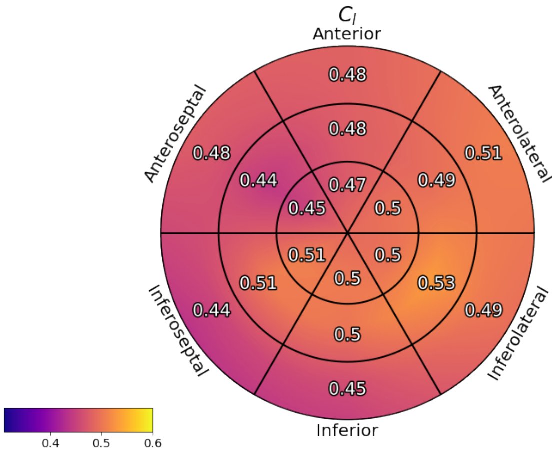

(b) de-TAC2 - c_l

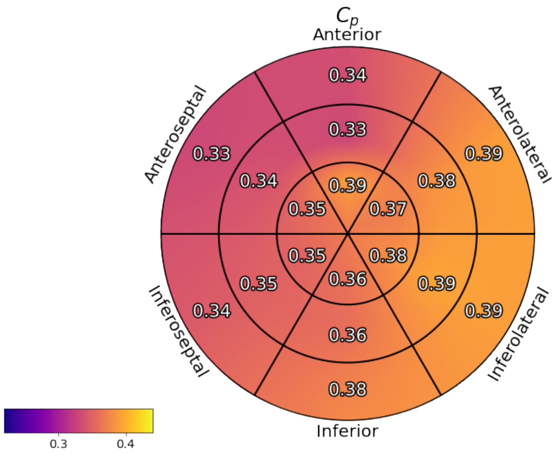

(c) de-TAC1 - c_p

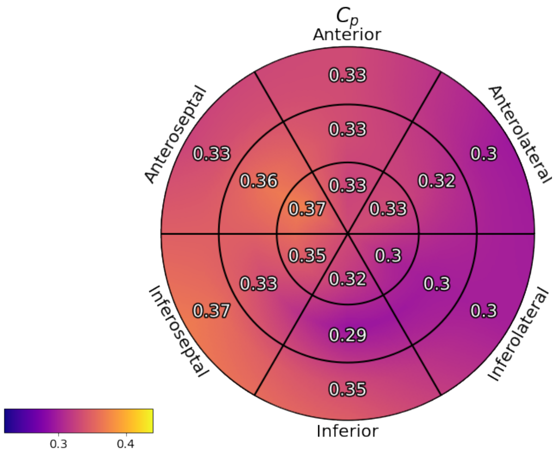

(d) de-TAC2 - c_p

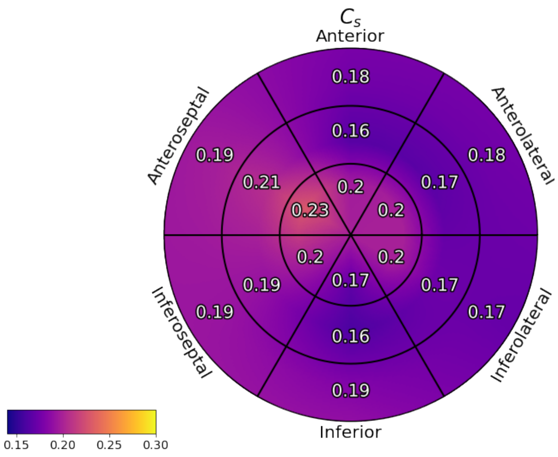

(e) de-TAC1 - c_s

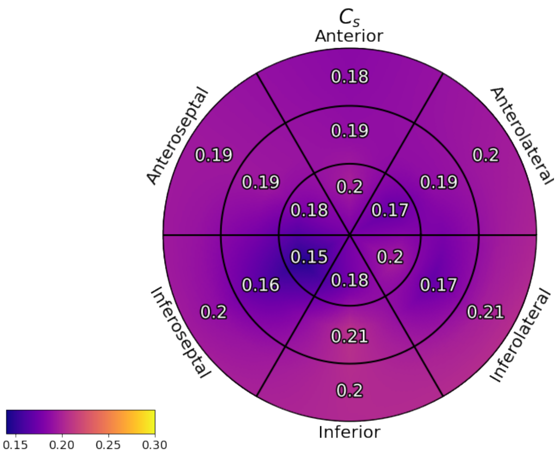

(f) de-TAC2 - c_s

Figure S.33.: The bullseye plots of the two de-TAC hearts' shape measures c_l, c_p and c_s are shown.

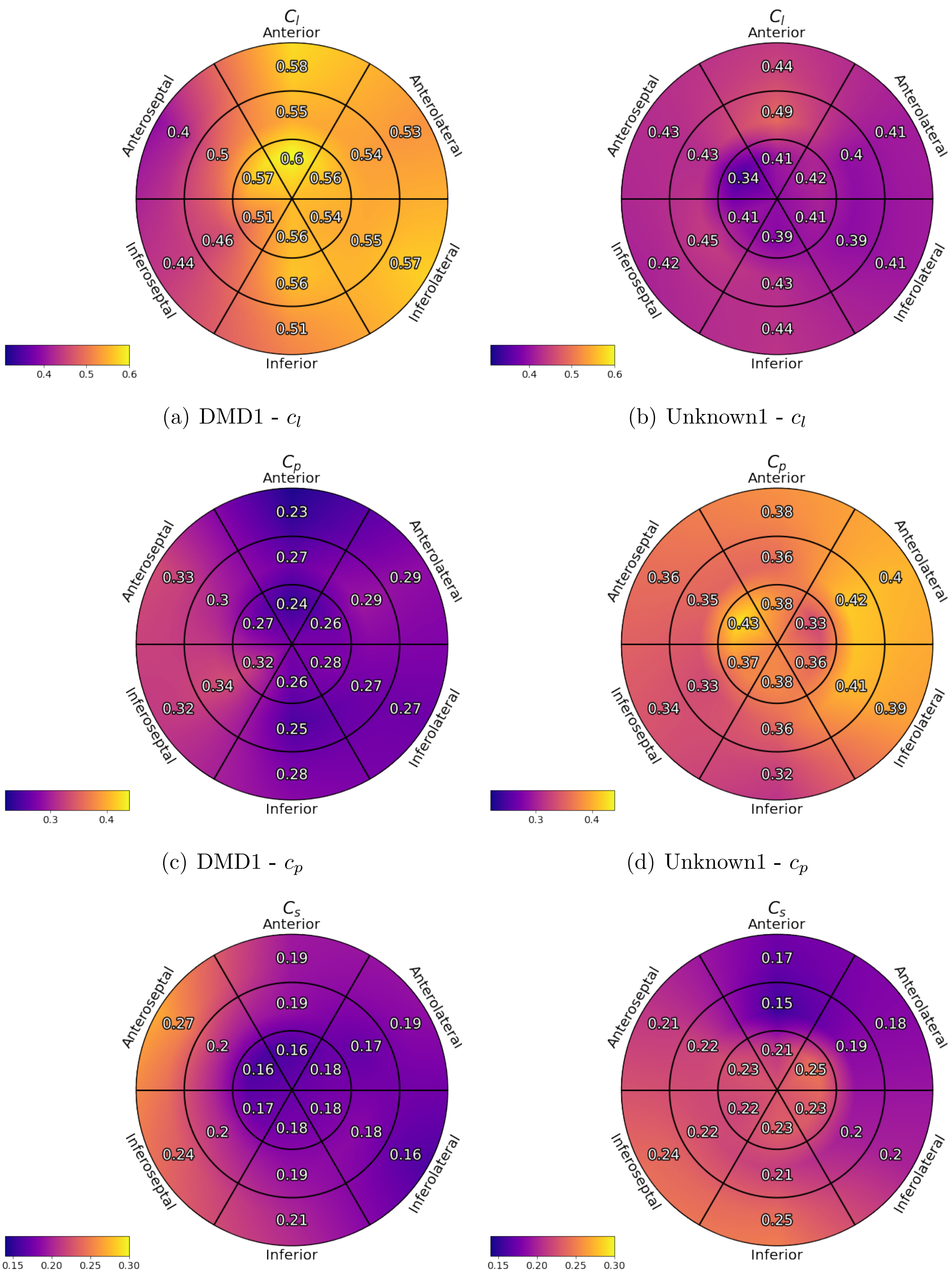

(a) DMD1 - c_l (b) Unknown1 - c_l

(c) DMD1 - c_p (d) Unknown1 - c_p

(e) DMD1 - c_s (f) Unknown1 - c_s

Figure S.34.: The bullseye plots of the DMD heart's shape measures c_l, c_p and c_s as well as the unknown heart model's shape measures c_l, c_p and c_s are shown.

S.1.8. Nematic order parameter

The average nematic order parameters S and η are displayed in the bullseye plots. For each sample, one bullseye plot is shown and they are grouped with respect to the different pathologies.
The ROI of the nematic order parameters has size 12 voxel in every dimension. Each ROI is used to calculated one nematic order parameter value pair (S, η). One heart slice with the nematic order parameter S is displayed in figure S.35.

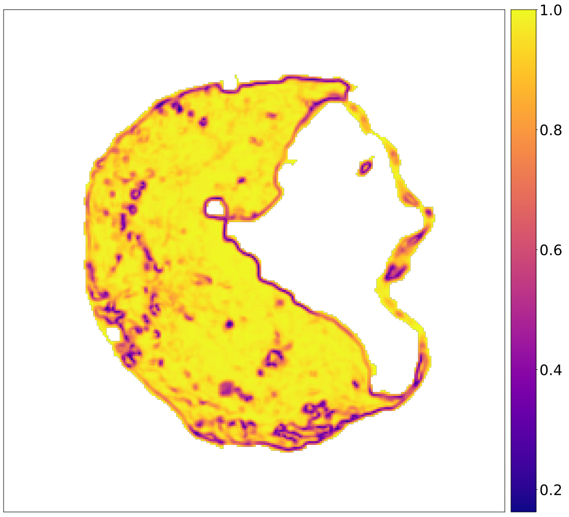

Figure S.35.: One slice of the MI2 heart's nematic order parameter S is shown.

Control hearts The bullseye plots of the control hearts' nematic order parameter S and η are shown in figure S.36. The samples Control1 and Control2 have average values of the nematic order parameter S ranging from 0.8 to 0.9 and the values show the tendency to increase from the basal segments to the apical segments.
The nematic order parameter η of sample Control1 ranges from 0.03 to 0.06, whereas the sample Control2's η values range from 0.02 to 0.07. Both sample show the decrease of the values from basal segments down to the apical segments. This behaviour is inversely proportional to the S values of the control samples.

MI The bullseye plots of the MI hearts' nematic order parameter S and η are shown in figure S.37. The nematic order parameter S has reduced values in the anterolateral segments as well as in the mid and apical anterior and anterospetal segments of the sample MI1. The sample MI2 has reduced S values in the inferior

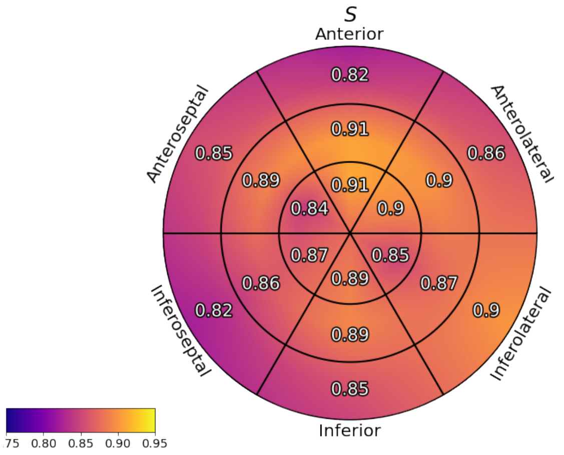

(a) Control1 - S

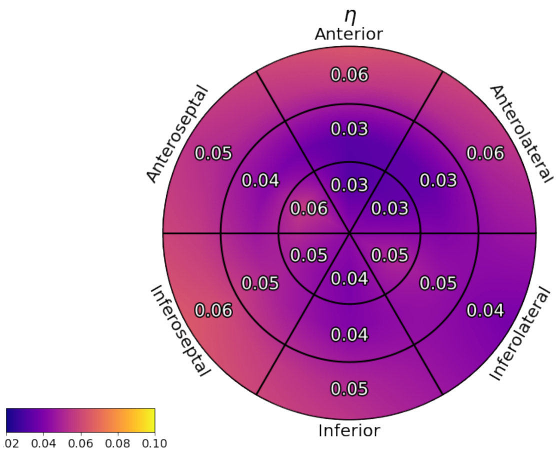

(b) Control1 - η

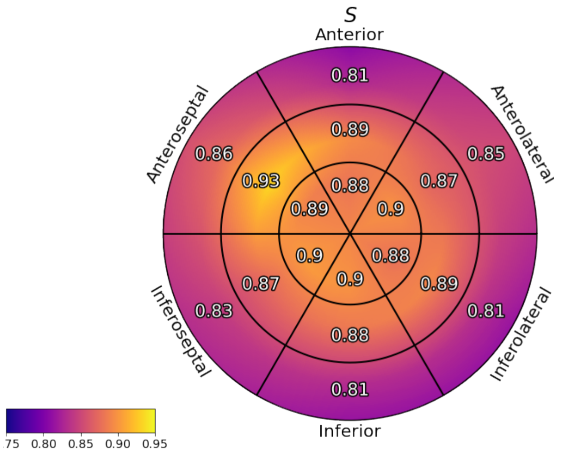

(c) Control2 - S

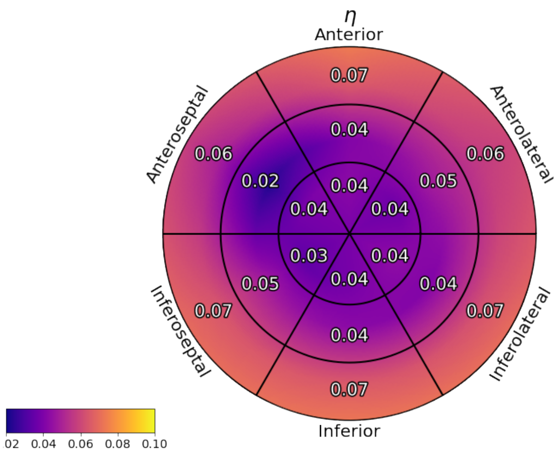

(d) Control2 - η

Figure S.36.: The bullseye plots of the two control hearts' nematic order parameters S and η are shown.

volume.

The nematic order parameter η has increased values in the anterolateral segments as well as in the mid and apical anterosetal and anterior segments and in the apical inferolateral segment compared to the control hearts. The apical anterolateral segment's η value lies above the 1-σ-interval of the control hearts. The sample MI2 has increased η values in the inferior segments.

TAC The bullseye plots of the TAC hearts' nematic order parameters S and η are shown in figures S.38 and S.39. The nematic order parameter S shows increased values for the sample TAC1, especially in the basal segments. The sample TAC2

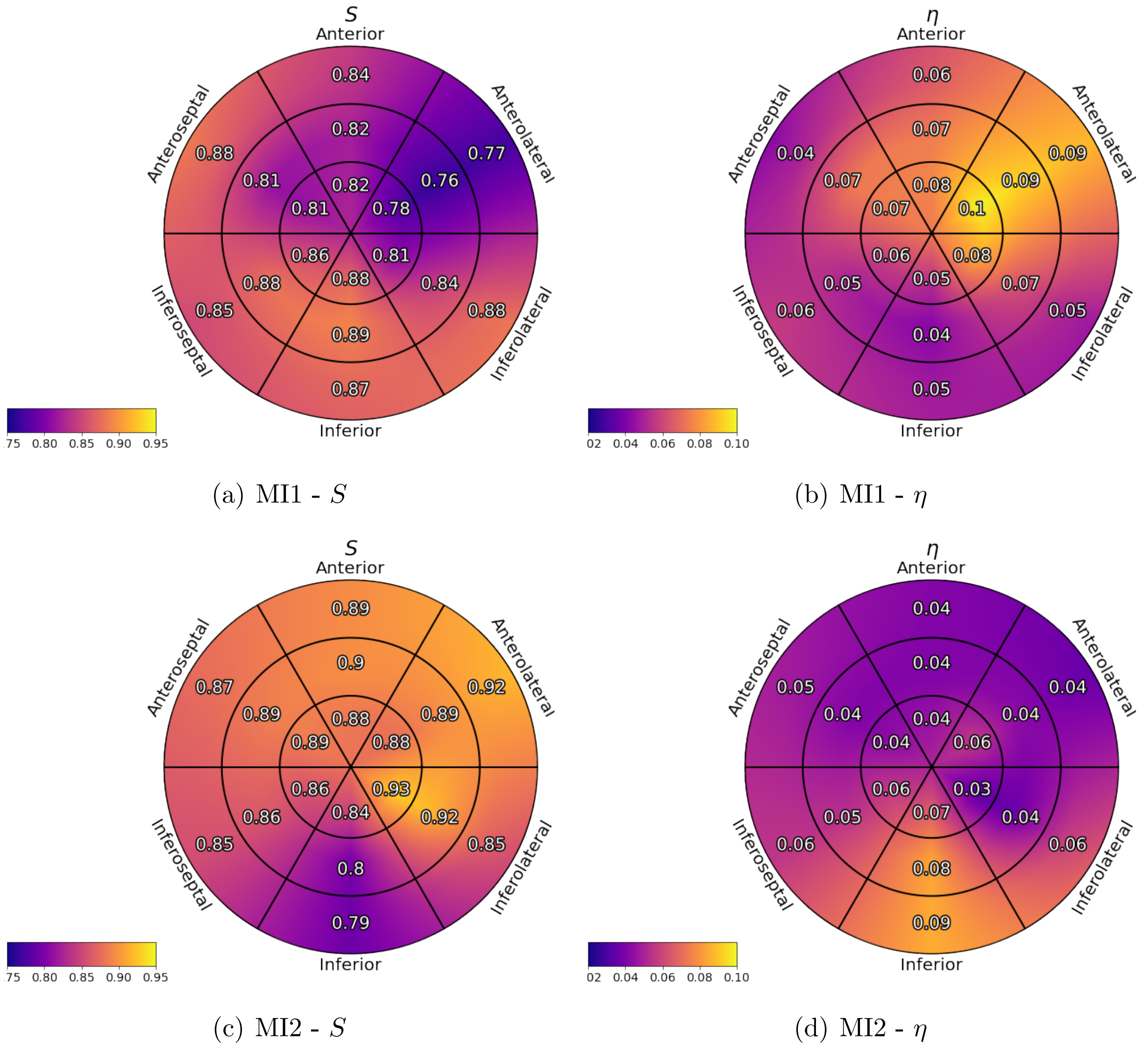

(a) MI1 - S

(b) MI1 - η

(c) MI2 - S

(d) MI2 - η

Figure S.37.: The bullseye plots of the two MI hearts' nematic order parameters S and η are shown.

shows increased S values in general but especially in the basal segments. The samples TAC3 and TAC4 have increased S values, in general.

The sample TAC1 has distinctly reduced nematic order parameter η values in the basal segments and the sample has slightly reduced η values in the mid segments. The η values are reduced in general in the sample TAC2, but especially in the basal segments. In general, the η values are reduced in sample TAC3, especially in the apical and basal segments. The sample TAC4 has reduced η values.

Shunt The bullseye plots of the shunt hearts' nematic order parameter S and η are shown in figure S.40. The nematic order parameter S is slightly reduced in the

sample Shunt1 in the apical and mid segments. The sample Shunt2 shows increased S values in general, but only slightly increased values in the antero- and inferoseptal segments. Also, increased S values are present in sample Shunt3.

The nematic order parameter η behaves inversely proportional to the nematic order parameter S in the case of the samples with a shunt. Therefore, the sample Shunt1's nematic order parameter η is slightly increased in the apical and mid segments. Furthermore, the sample Shunt2 shows reduced η values in general, but only slightly reduced values in the antero- and inferoseptal segments. Reduced η values are present in sample Shunt3.

de-TAC The bullseye plots of the de-TAC hearts' nematic order parameter S and η are shown in figure S.41. The nematic order parameter S is increased in sample de-TAC1 in the basal segments, especially in the anterior and inferior segments. Additionally, the sample de-TAC2 has increased S values in general and especially in the basal segments.

As in the case of the shunt model, the nematic order parameter η is inversely proportional to the S values. Thus, the η values are reduced in sample de-TAC1 in the basal segments, especially in the anterior and inferior segments. The sample de-TAC2 has reduced η values, in general, and especially in the basal segments.

DMD The bullseye plots of the DMD heart's nematic order parameter S and η are shown in figures S.42(a) and S.42(b). The sample DMD1 has increased nematic order parameter S values in the anterior, anterolateral, inerolateral and inferior segments as well as in the apical anteroseptal segment, but distinctly reduced S values in the basal anteroseptal segment.

As before, the nematic order parameter η is inversely proportional to the nematic order parameter S.

Unknown The bullseye plots of the unknown heart model's nematic order parameter S and η are shown in figures S.42(c) and S.42(d). The nematic order parameter S is reduced in the apical segment of sample Unknown1 and the sample has increased η values in the apical segments.

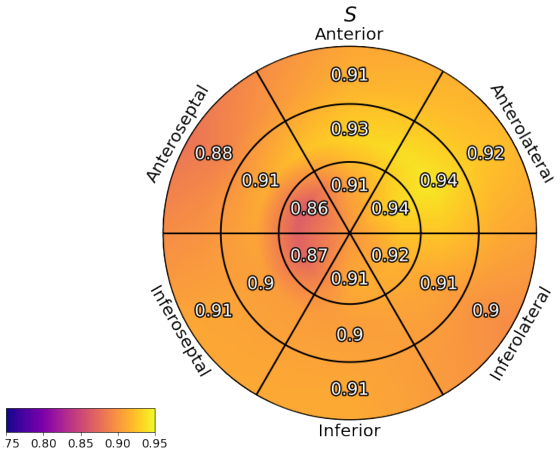

(a) TAC1 - S

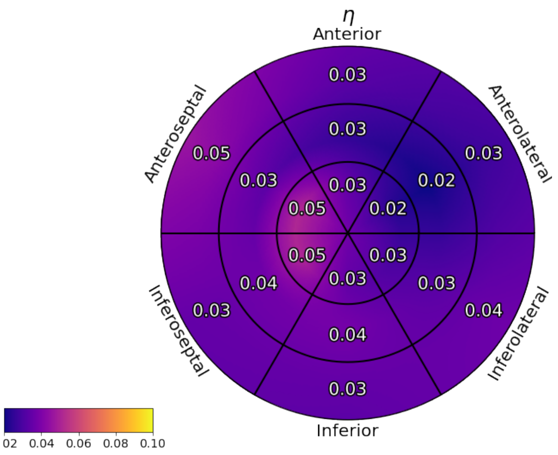

(b) TAC1 - η

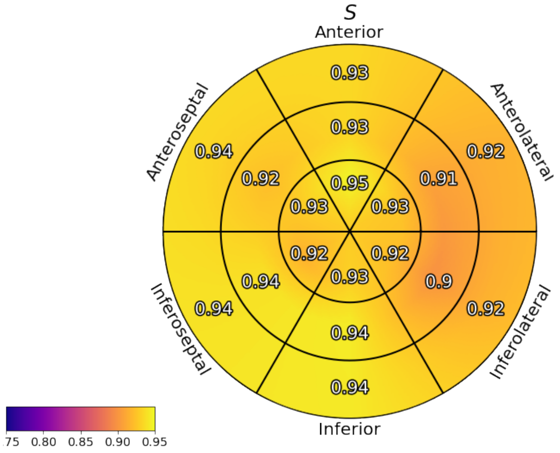

(c) TAC2 - S

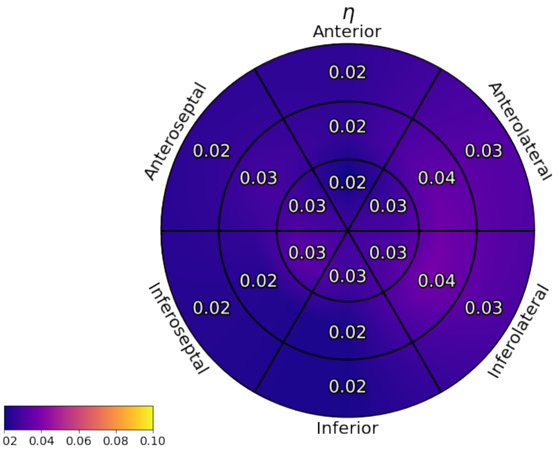

(d) TAC2 - η

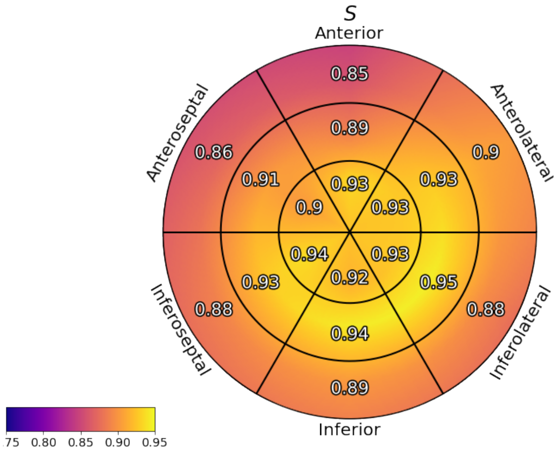

(e) TAC3 - S

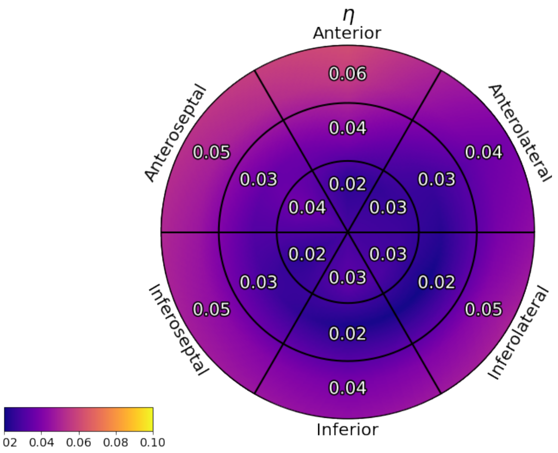

(f) TAC3 - η

Figure S.38.: The bullseye plots of three TAC hearts' nematic order parameters S and η are shown.

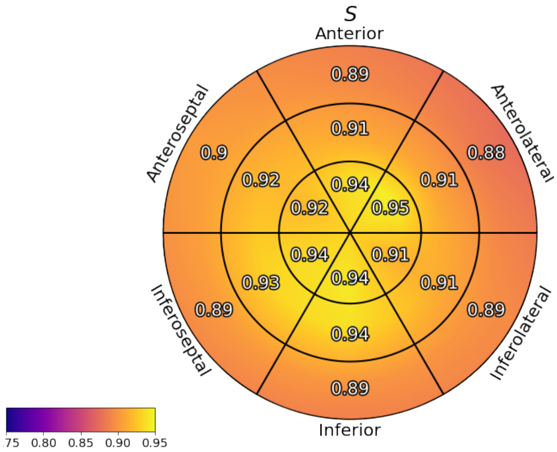

(a) TAC4 - S

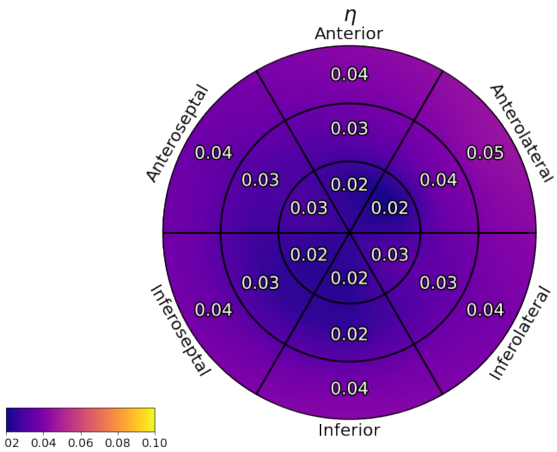

(b) TAC4 - η

Figure S.39.: The bullseye plots of the last TAC heart's nematic order parameters S and η are shown.

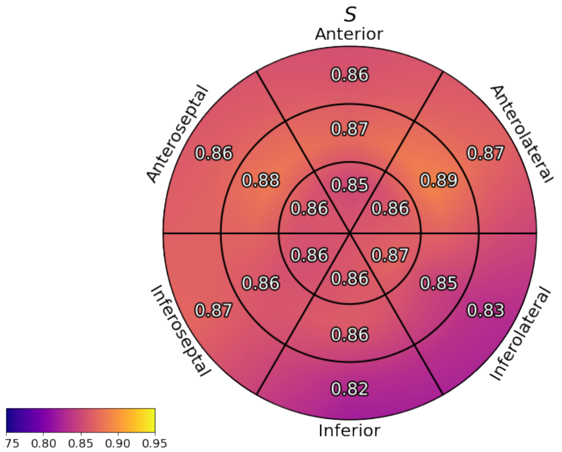

(a) Shunt1 - S

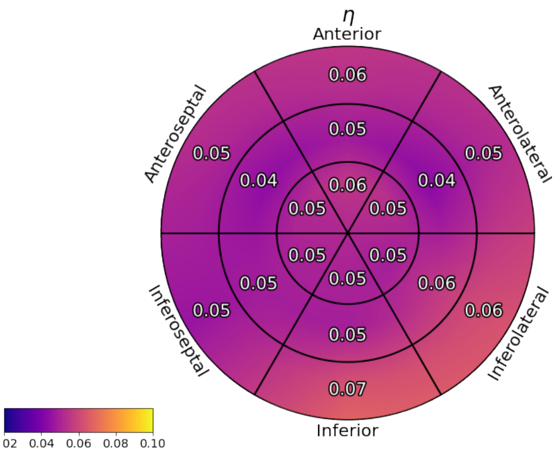

(b) Shunt1 - η

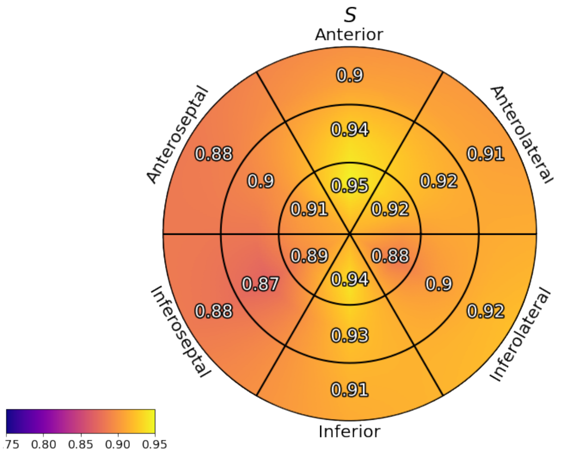

(c) Shunt2 - S

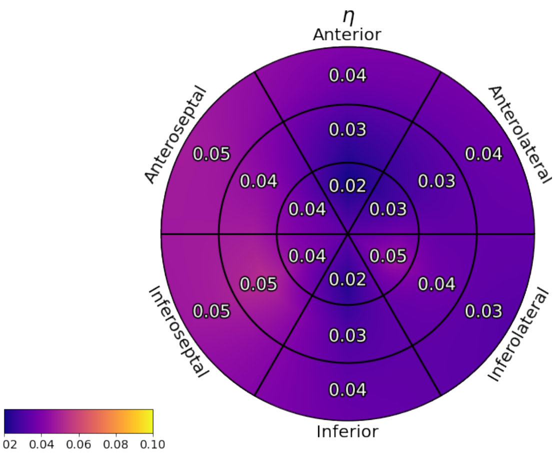

(d) Shunt2 - η

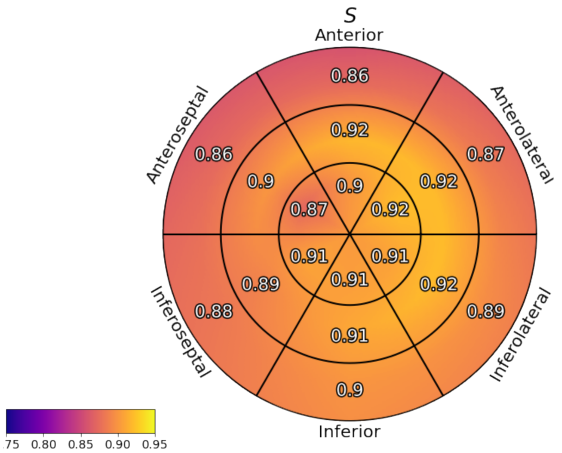

(e) Shunt3 - S

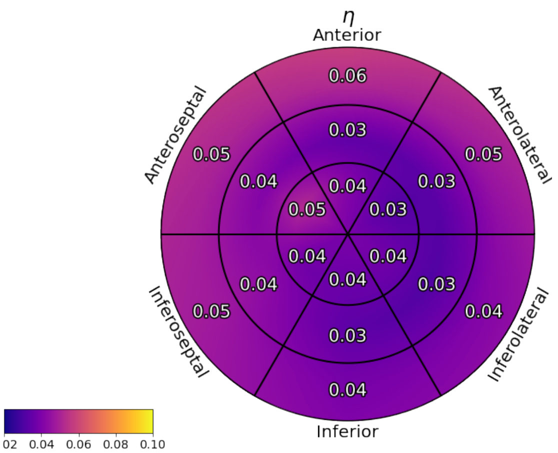

(f) Shunt3 - η

Figure S.40.: The bullseye plots of the three shunt hearts' nematic order parameters S and η are shown.

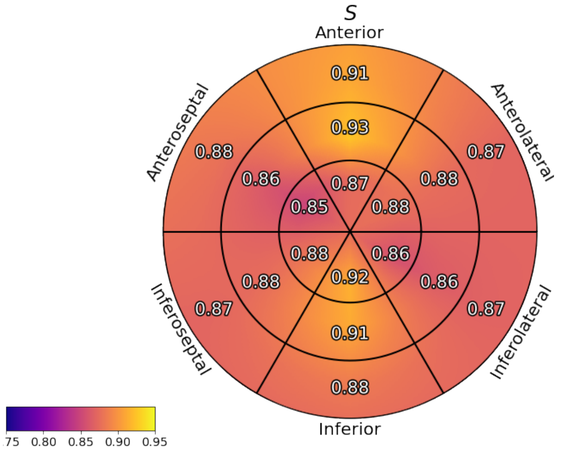

(a) de-TAC1 - S

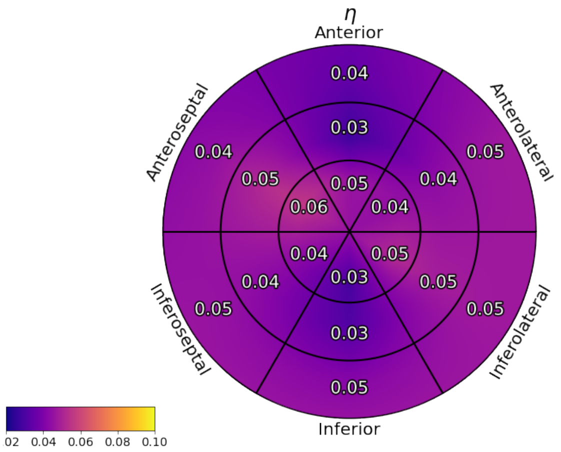

(b) de-TAC1 - η

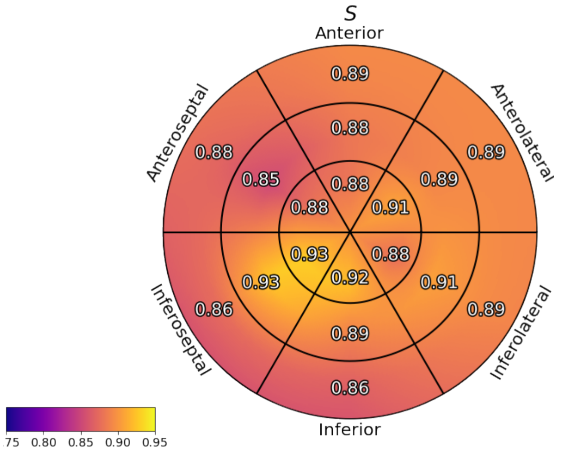

(c) de-TAC2 - S

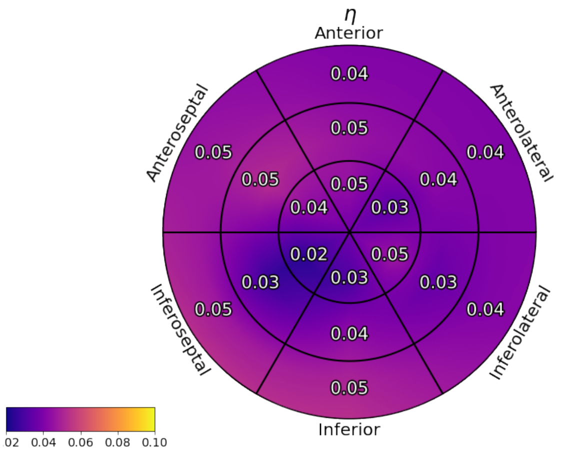

(d) de-TAC2 - η

Figure S.41.: The bullseye plots of the two de-TAC hearts' nematic order parameters S and η are shown.

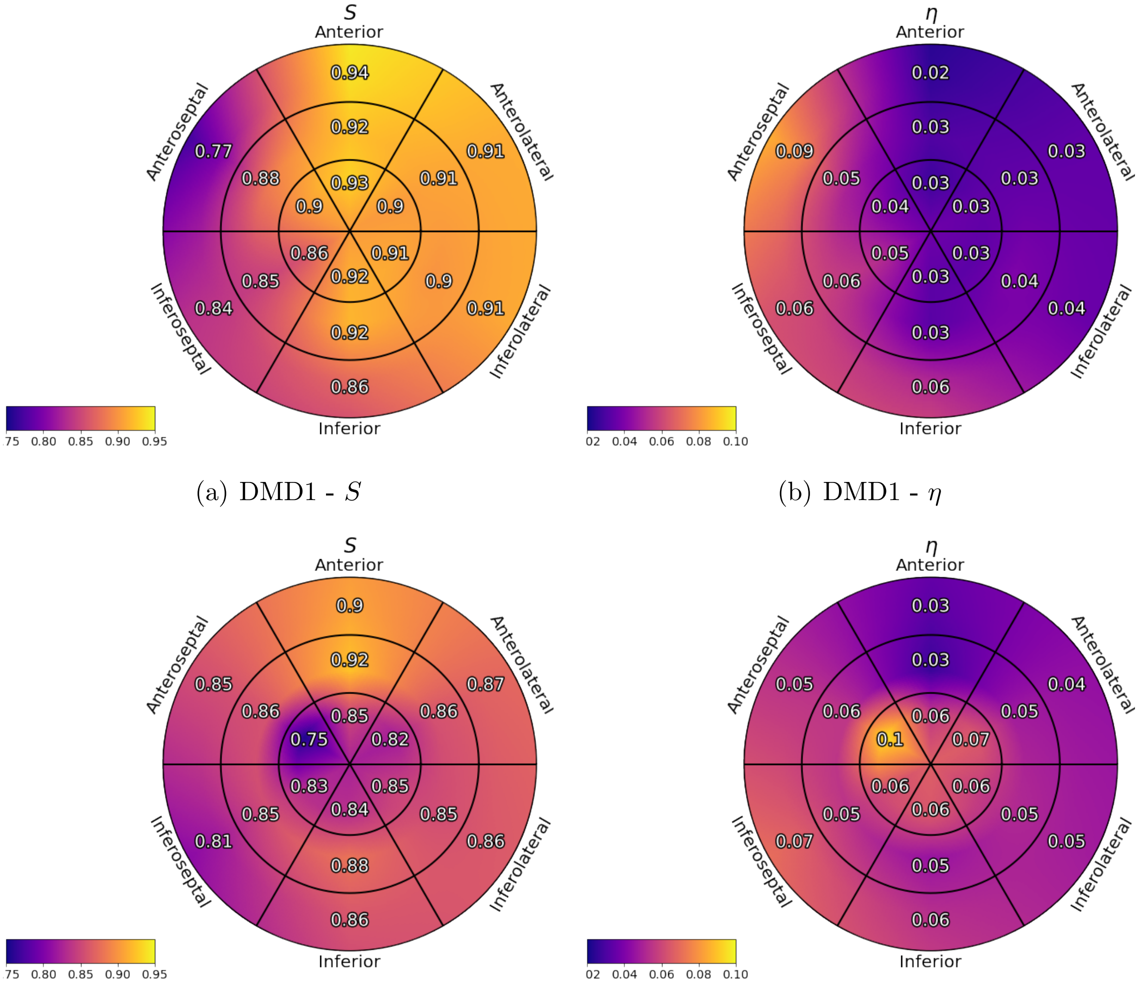

(a) DMD1 - S

(b) DMD1 - η

(c) Unknown1 - S

(d) Unknown1 - η

Figure S.42.: The bullseye plots of the DMD heart's nematic order parameters S and η as well as the unknown heart model's nematic order parameters S and η are shown.
